# Facilitating crRNA Design by Integrating DNA Interaction Features of CRISPR‐Cas12a System

**DOI:** 10.1002/advs.202501269

**Published:** 2025-05-08

**Authors:** Zhihao Yao, Wanglu Li, Kaiyu He, Hongmei Wang, Yan Xu, Qun Wu, Liu Wang, Yao Nie

**Affiliations:** ^1^ The Key Laboratory of Industrial Biotechnology Ministry of Education State Key Laboratory of Food Science and Resources School of Biotechnology Jiangnan University Wuxi Jiangsu 214122 China; ^2^ State Key Laboratory for Managing Biotic and Chemical Threats to the Quality and Safety of Agro‐products Institute of Agro‐product Safety and Nutrition Zhejiang Academy of Agricultural Sciences Hangzhou 310021 China; ^3^ Key Laboratory of Information Traceability for Agricultural Products Ministry of Agriculture and Rural Affairs Hangzhou 310021 China

**Keywords:** activity prediction, CRISPR‐Cas12a, deep learning, molecular dynamics simulation, trans‐cleavage

## Abstract

The CRISPR‐Cas12a system has gained significant attention as a rapid nucleic acid diagnostic tool due to its crRNA‐guided *trans*‐cleavage activity. Accurately predicting the activity of different targets is significant to facilitate the crRNA availability but remains challenging. In this study, a novel approach is presented that combines molecular dynamics simulations and neural network modeling to predict the *trans*‐cleavage activity. Unlike conventional tools that rely solely on the base sequences, our method integrated sequence features and molecular interaction features of DNA in the CRISPR‐Cas12a system, significantly improving prediction accuracy. Through feature importance analysis, key sequence features that influence Cas12a *trans*‐cleavage activity are identified. Additionally, a crRNA‐DNA library with over 23 456 feature sequences from representative viruses and bacteria is established, and validated the high predictive accuracy of the model (Pearson's *r* = 0.9328) by screening crRNAs from reference targets. This study offers new insights into the molecular interactions of Cas12a/crRNA‐DNA and provides a reliable framework for optimizing crRNA design, facilitating the application of the CRISPR‐Cas12a in rapid nucleic acid diagnostics.

## Graphical abstract

This study introduces a novel approach combining molecular dynamics simulations and neural network modeling to predict the Cas12a *trans*‐cleavage activity. By integrating sequence and molecular interaction features, prediction accuracy is enhanced, and identify key features affecting Cas12a *trans*‐cleavage activity. A comprehensive crRNA‐DNA library is established, demonstrating the model's high accuracy in facilitating crRNA design for nucleic acid diagnostics.

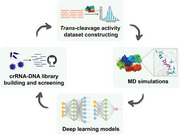

## 1 Introduction

Recently, the CRISPR‐Cas12a system has been widely applied into in vitro nucleic acid detection in medical diagnosis, food processing and safety, and environmental monitoring due to its strong ability to recognize specific nucleic acid targets and activate CRISPR RNA (crRNA)‐guided *trans*‐cleaving activity to enhance signals.^[^ ^1^, ^2^ ^]^ However, different crRNAs show greatly different effects on Cas12a enzyme activities due to its sequence dependence.^[^ ^3^, ^4^, ^5^ ^]^ It is thereof important to screen optimal crRNA to facilitate the applications of CRISPR‐based technologies, such as improving quantitative abilities of one‐pot Cas12a‐amplifcation detection systems,^[^ ^6^ ^]^ and enhancing the ability of gene editing, multi‐target detection, and mutant identification.^[^ ^7^ ^]^ Conventional crRNA screening procedures often involve multiple comparative assays in laboratory experiments, that is time‐consuming and resource‐intensive.^[^ ^8^, ^9^ ^]^ By comparison, computer‐based methods allow researchers to conduct tests in a virtual environment, reducing the cost of experiments.^[^ ^10^ ^]^ Meanwhile, they can quickly run multiple trials and iterations, saving a lot of time, especially when dealing with long time series or multiple scenarios.^[^ ^11^ ^]^ Recently, several in silico deep learning‐based tools such as ADAPT^[^ ^12^ ^]^ and EasyDesign^[^ ^13^ ^]^ have been developed to screen crRNA and predict enzyme activity of CRISPR‐Cas systems. However, these tools only established a link between base sequences and enzyme activity, showing limited prediction performance without considering the CRISPR‐Cas molecular interaction mechanism strongly related with *trans*‐cleavage activity.

As a RNA‐guided nuclease, the CRISPR‐Cas12a molecular interactions mainly include four steps (Figure S1, Supporting Information): 1) protospacer adjacent motif (PAM) recognition by the WED and PI domains of Cas12a to promote double‐strand DNA (dsDNA) target unwinding; 2) the hybridization of crRNA and DNA target to induce conformational changes in REC lobe of Cas12a, leading to activate RuvC domain of Cas12a; 3) the *cis*‐cleavage of DNA target and release of PAM‐distal dsDNA; 4) the *trans*‐cleavage of non‐specific single‐stranded DNA (ssDNA) at RuvC domain.^[^ ^14^, ^15^, ^16^ ^]^ Therefore, we infer that the intermolecular and intramolecular interactions of the preceding Cas12a/crRNA‐DNA complex (at step 1–3) can directly affect the binding and cleaving efficiency of the subsequent non‐specific ssDNA substrates (at step 4).^[^ ^17^, ^18^ ^]^ For example, PAM in the DNA target could interact with PI domain of Cas12a (at step 1), different PAM sequences showed different effects on both the Cas12a *cis*‐cleavage and Cas12a *trans*‐cleavage activity.^[^ ^16^, ^19^ ^]^ Additionally, the free energy change of crRNA‐DNA targets from the initial state to approximate hybridization state (at step 2) was found as a key factor controlling *trans*‐cleavage activity of Cas12a.^[^ ^20^ ^]^ However, it has not been investigated about deeper molecular interaction features between Cas12a/crRNA and DNA targets (from step 3 to step 4, after the release of PAM‐distal dsDNA). Thereby, we believe that further analyzing these molecular interaction features from step 3 to step 4 would effectively explain the reason of the differences in Cas12a *trans*‐cleavage activity caused by different sequences.

Currently, the emerging molecular dynamics (MD) simulation technology has been increasingly used to investigate CRISPR molecular interaction mechanisms. For example, by measuring the binding free energy of Cas9/gRNA‐DNA complex with MD simulations, a tool named CRISOT was developed to predict CRISPR‐Cas9 off‐target efficiencies with higher prediction performances than other off‐target scoring methods (*p* < 0.05).^[^ ^21^ ^]^ Additionally, the MD simulation technology was used to reveal that DNA binding could induce a switch to change conformation and activate Cas12a (as described above, at step 2) by measuring the root mean square deviation (RMSF) and trajectory.^[^ ^22^ ^]^ Moreover, MD simulations also revealed that an alpha‐helical lid located in the RuvC domain of Cas12a could guide the target strand (TS) of DNA toward catalysis by calculating the binding free energy change profiles (at step 3).^[^ ^23^ ^]^ Based on these, we speculated that using the MD simulation technology to calculate molecular interaction features such as RMSF and free energy change (from step 3 to step 4) could promote the understanding of the differences in Cas12a *trans*‐cleavage activity caused by base‐pair types and positions.

To reveal deeper molecular interaction mechanisms, in this study, we conducted MD simulations of various Cas12a/crRNA‐DNA complexes (at step 3–4, without PAM‐distal dsDNA) to obtain a broad molecular interaction feature map of DNA. By incorporating these interaction features, we developed a neural network model (ActivityNN) to accurately predict the Cas12a *trans*‐cleavage activity. Notably, to reduce MD computational resources and time, we also proposed an efficient model (IntermediateNN) to compute the interaction features with less than 3 min. More importantly, by analyzing the feature importance of ActivityNN, we identified key sequence features influencing Cas12a *trans*‐cleavage activity. By using the developed models, we created a crRNA‐DNA library targeting more than 23 456 feature sequences from representative dsDNA viruses and bacteria, and screened crRNAs from reference microorganisms to perform experimental tests, demonstrating the great performance of developed models in predicting the Cas12a *trans*‐cleavage activity and enhancing the crRNA availability.

## 2 Results

### 2.1 Design Principle and Workflow

**Figure 1:**
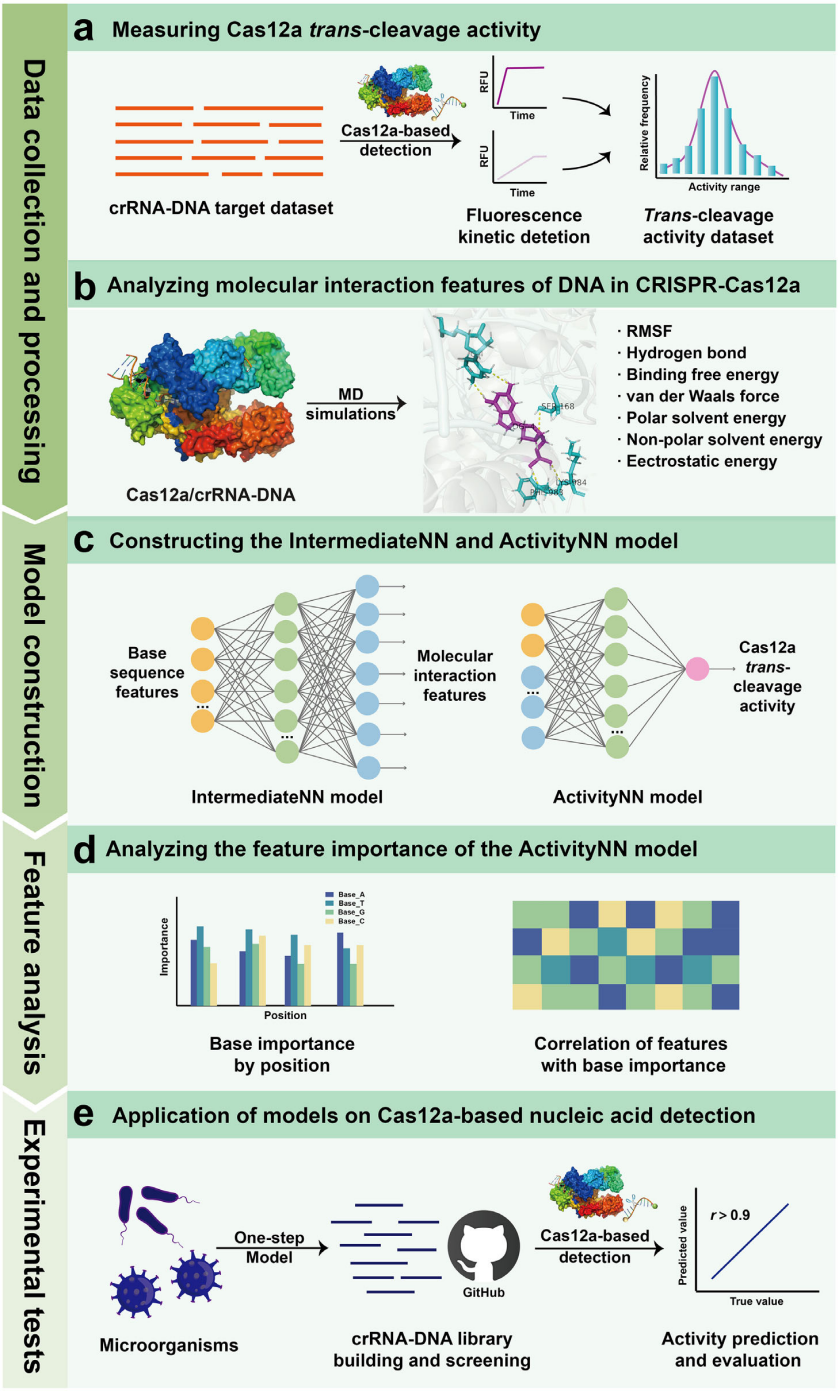
Schematic illustration of this study. a) Measuring the Cas12a *trans*‐cleavage activity from the broad crRNA‐DNA datasets by conducting real‐time fluorescence kinetic detection. b) Analyzing molecular interaction feature of DNA in the CRISPR‐Cas12a system by MD simulations, including RMSF, hydrogen bond cunts, total binding free energies, van der Waals forces, polar solvent energies, non‐polar solvent energies, and electrostatic energies. c) Constructing the neural network models (IntermediateNN and ActivityNN) for predicting molecular interaction features and Cas12a *trans*‐cleavage activity. d) Feature importance analysis of the ActivityNN model to identify key sequence features. e) Application of models on Cas12a‐based nucleic acid detection by building a crRNA‐DNA library (available on the github website) to screen crRNAs and predict their Cas12a *trans*‐cleavage activity.

The main goal of this study is to use MD simulations to comprehend the molecular interaction features (such as binding energies, hydrogen bonds, and RMSF) of different DNA targets in the CRISPR‐Cas12a system, and then apply this knowledge to predict the Cas12a *trans*‐cleavage activity and facilitate the crRNA availability. The schematic overview of this study is shown in **Figure** **1**, including data collection and processing, model construction, feature importance analysis, and experimental tests.

Specifically, we first designed crRNAs of various targets, including PAM‐included (5’‐TTTN‐3’, N = A/T/C/G) and PAM‐free crRNAs. Then, we determined the Cas12a *trans*‐cleavage activity of the prepared crRNA‐DNA dataset by using real‐time fluorescence detection (Figure 1a). Meanwhile, we used MD simulations to calculate various molecular interaction features of DNA as ligands in the CRISPR‐Cas12a system, including hydrogen bond counts, RMSF, total binding free energies, and free energy decompositions (van der Waals forces, polar solvent energies, non‐polar solvent energies, and electrostatic energies), because these features may impact the Cas12a *trans*‐cleavage activity (Figure 1b). We then established two neural network models named IntermediateNN and ActivityNN. The IntermediateNN model could predict the molecular interaction features of DNA in the CRISPR‐Cas12a system. For the ActivityNN model, it was developed to predict the Cas12a *trans*‐cleavage activity by training a dataset including target sequence and molecular interaction features (Figure 1c). Subsequently, we performed the feature importance analysis of the ActivityNN model, identifying the key sequence features of the CRISPR‐Cas12a system (Figure 1d). Finally, the developed models were used to build a large crRNA‐DNA library and screen crRNAs of reference targets and predict the corresponding Cas12a *trans*‐cleavage activity (Figure 1e).

### 2.2 Measuring Cas12a Trans‐Cleavage Activity of Broad crRNA‐DNA Dataset

To create a dataset that represents actual Cas12a‐based in vitro detection procedures, we constructed a broad dataset including 1448 crRNA‐DNA complexes (Group 1: 60 targets, Group 2: 120 targets, and Group 3: 1268 targets). These complexes were targeted several species‐specific genes of various microorganisms such as *Lactobacillus acetotolerans*, *Pichia kudriazevii*, *Spiroplasma eriocheiris*, *Salmonella typhimurium*, white spot syndrome virus, SARS‐CoV‐2, and human papillomavirus type 18 (HPV18) from food processing, food safety, and medical diagnostics.^[^ ^6^, ^13^, ^24^, ^25^, ^26^ ^]^ Detailed descriptions on the construction of crRNA‐DNA dataset can be found in the Methods section. The analyzed base sequences included 20 nucleotide (20‐nt) specific sequences (defined as position 1–20 in this study) located on the target strand (TS, 5’–3’) of DNA, 4‐nt PAM sequence (position 21–24) located on the non‐target strand (NTS, 5’–3’), and 4‐nt PAM complementary sequence (position 25–28) located on the target strand (TS, 5’–3’) (Figure S2, Supporting Information). The base distribution of all prepared DNA targets is shown in Figure S3 (Supporting Information). The four kinds of bases (A, T, G, C) widely distributed at all positions, and the average frequency was 24.55%, 34.61%, 20.60%, and 20.24%.

We then measured the Cas12a *trans*‐cleavage activity of the broad crRNA‐DNA dataset by real‐time fluorescence kinetics detection (see Experimental Section). We collected the increased fluorescence signals from the initial 10 min and obtained the Cas12a *trans*‐cleavage activity by calculating the first derivative of fluorescence growth with respect to time (Figures S4–S8, Supporting Information). Consequently, a map of the Cas12a *trans*‐cleavage activity was generated by normalizing the increased fluorescence signals under the same reaction conditions (see Experimental Section) (Figures S9–S11, Supporting Information). Results showed that the overall Cas12a *trans*‐cleavage activity was normally distributed in the range of 0–0.12 relative fluorescence units (RFU)/min, and the most targets were distributed in the range of 0.01–0.02 RFU/min (28.591%), followed by 0–0.01 RFU/min (20.235%) and 0.02–0.03 RFU/min (17.334%), further supporting the generalizability of these data.

### 2.3 Molecular Interaction Features of DNA in the CRISPR‐Cas12a System

**Figure 2:**
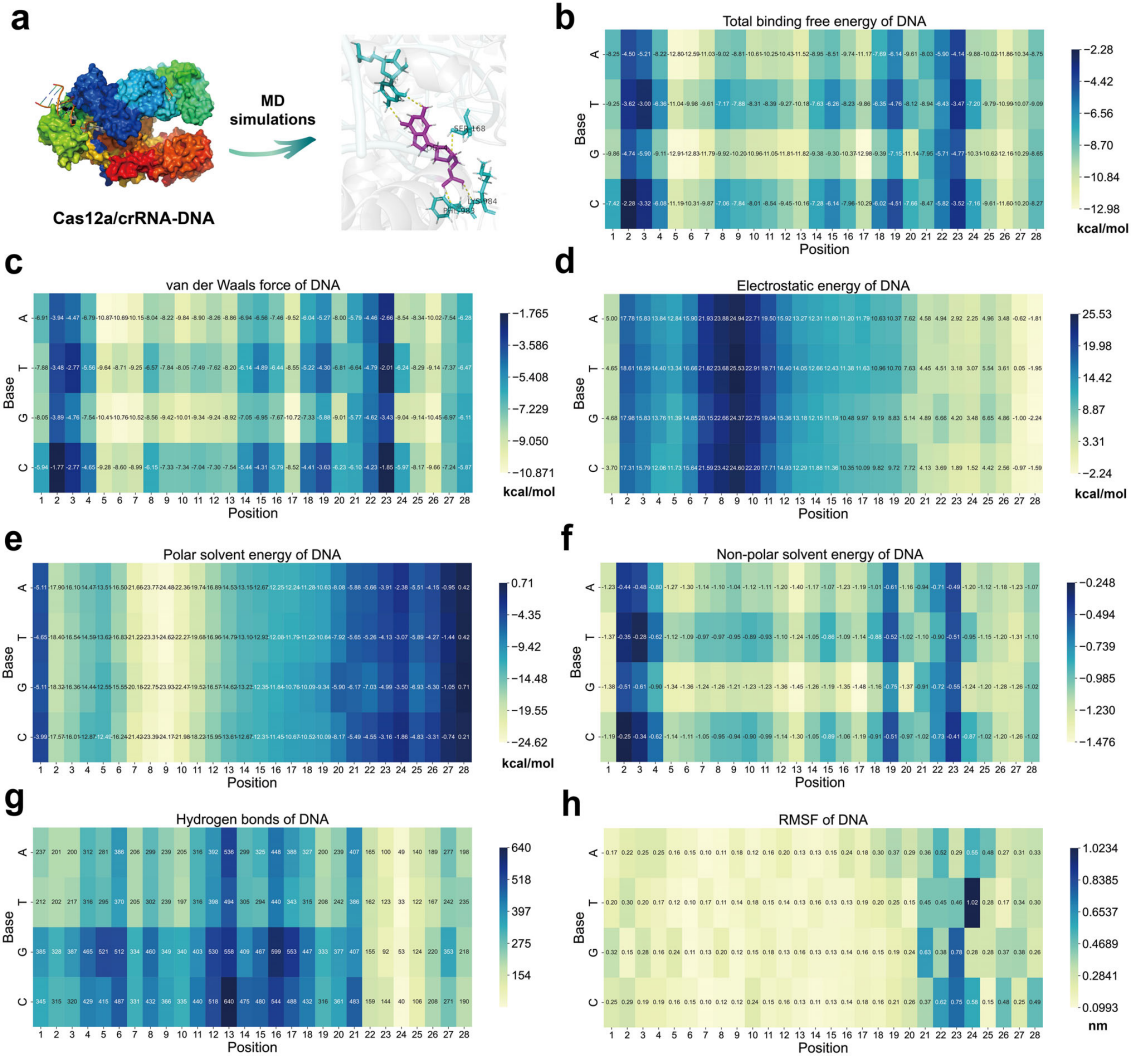
Molecular interaction features of DNA in the CRISPR‐Cas12a system across the base positions and base types. a) scheme of MD simulations of Cas12a/crRNA‐DNA complexes (PDB ID: 5XUS), exploring the dynamics and stability of molecular interactions. b–h) Heat map representation of key molecular interaction features of DNA from trajectories during 90–100 ns simulations, including average energy distributions (b) total binding free energies, c) van der Waals forces, d) non‐polar solvent energies, e) electrostatic energies, and f) polar solvent energies), g) hydrogen bond counts, and h) RMSF, across base positions in the target (1–20), PAM (21–24), PAM complementary (25–28) sequences and base types (A, T, G, C).

We constructed 180 different Cas12a/crRNA‐DNA complexes (from step 3 to step 4, without PAM‐distal dsDNA, Figure S1, Supporting Information) by mutating nucleic acid bases of crRNA and DNA in this study. Then, we performed 100 ns MD simulations in explicit solvent to estimate the similarities and differences in the dynamics across these complexes (**Figure** **2**a). To promote the understanding of the molecular interactions in the CRISPR‐Cas12a system (Cas12a/crRNA as the receptor, DNA as the ligand) and explore possible stable and flexible regions related to Cas12a *trans*‐cleavage activity, we analyzed several important features of DNA according to the position of base sequence (28‐nt), including total binding free energies, van der Waals forces, polar solvent energies, non‐polar solvent energies, electrostatic energies, hydrogen bond counts, and RMSF. Notably, to facilitate the calculation of these features, the trajectories corresponding to 90–100 ns in the MD simulations were selected for analysis, because the Root mean square deviation (RMSD) value were relatively stable with continually fluctuation of all complexes in the entire (100 ns) simulation trajectory (Figure S12, Supporting Information). The fluctuated RMSD values showed that the structures would exhibit some conformational changes within this timeframe, that could capture potential and meaningful molecular interactions. Importantly, the overall change trend of RMSD remained relatively stable across the simulations, that suggested that the initial structures were stable during the 100 ns period. To further confirm the structural relative stability of this stage (90–100 ns), we selected three test targets with high, medium, and low Cas12a *trans*‐cleavage activities to analyze the RMSD distributions for both the 100 ns and 90–100 ns intervals (Figure S13, Supporting Information). Results showed that the RMSD distributions for both 100 ns and 90–100 ns followed a normal distribution, and the mean value was close. This suggested that the 90–100 ns simulation could provide reliable and stable results, that is crucial for reducing simulation time and improving throughput. Additionally, we chose the initial Cas12a/crRNA‐DNA complex structure from step 3 to step 4 without PAM‐distal dsDNA for our MD simulations. Since this structure was closer to step 4, we believed the stage (90–100 ns) was closer to the Cas12a *trans*‐cleavage stage (step 4, Figure S1, Supporting Information).

According to each base type (A, T, G, C), we analyzed the average RMSF, hydrogen bond counts, and energy distribution of DNA across all complexes at each base position (Figure 2b–h). It can be found that several features were strongly related to base locations and base types such as total binding free energies, van der Waals forces, non‐polar solvent energies, and hydrogen bond counts (Figure 2b,c,f,g). For example, for the G base on the target sequence (position 1–20), the total binding free energies (Figure 2b), van der Waals forces (Figure 2c), and non‐polar solvent energies (Figure 2f) are lowest at most positions, indicating that the G base may play a pivotal role in maintaining strong binding interactions and overall stability, and enhancing cleaving activity.^[^ ^20^ ^]^ For instance, at position 7, the G base (Base_7_G, compared with A, T, C) showed the lowest total binding energies (−11.790 kcal mol^−1^), lowest van der Waals forces (−10.524 kcal mol^−1^), and lowest non‐polar solvent energies (−1.236 kcal mol^−1^). Moreover, at position 24, the T base (Base_24_T) showed the lowest hydrogen bond counts (33), indicating that the T base may be less desirable at this position. On the target sequence (position 1–20), the hydrogen bond counts of the G/C base were more than that of the A/T base (Figure 2g), further suggesting that G/C interactions contribute more significantly to structural stability in the target region. By comparison, the hydrogen bond counts were close for four kinds of bases (A, T, G, C) on the PAM sequence (position 21–24) and PAM complementary sequence (position 25–28), indicating that the hydrogen bond distribution in PAM sequence was relatively balanced. This distribution may contribute to the functional performance and recognition properties of PAM sequence. Therefore, in some situations, even PAM‐free sequences could also maintain the necessary enzymatic activity of Cas12a protein.^[^ ^6^, ^26^ ^]^

Additionally, it can be found that some features were related to the base position with no obvious difference of the base type, such as electrostatic energies, polar solvent energies, and RMSF (Figure 2d,e,h). Therefore, we further calculated the average RMSF, energy distribution, and hydrogen bond counts of all complexes at each base position (Figure S14, Supporting Information). The distribution of these features varied at different locations. Results showed that base position 7, 8, 9, and 10 (located on target sequences, TS, 5’‐3’) had high electrostatic energies (21.121, 23.586, 25.149, and 22.708 kcal mol^−1^), low polar solvent energies (−20.982, −23.402, −24.432, and −22.369 kcal mol^−1^), and low RMSF (0.118, 0.122, 0.136, and 0.148 nm), indicating these positions may be structurally stable regions important for Cas12a protein binding, and remain intact after releasing the PAM‐distal products.^[^ ^14^ ^]^ In contrast, position 21, 22, 23, and 24 (located on PAM sequences, NTS, 5’‐3’) showed low electrostatic energies (4.563, 4.819, 3.174, and 2.520 kcal mol^−1^), high polar solvent energies (−5.725, −5.456, −4.070, and −2.584 kcal mol^−1^), and high RMSF (0.448, 0.470, 0.501, and 0.534 nm), suggesting these positions may be more flexible regions for modulating the conformational change of Cas12a PI domain.^[^ ^16^ ^]^

Overall, we provided a comprehensive map of the differences in molecular interaction features DNA in the CRISPR‐Cas12a system (Figure 2), that was the important basis for exploring the difference of Cas12a *trans*‐cleavage activity. Based on differences of molecular interaction features, we speculated that building a mathematical model would help effectively optimize bases at different locations, thereby improving the Cas12a *trans*‐cleavage activity.

### 2.4 Building Effective Models to Predict Molecular Interaction Features and Cas12a Trans‐Cleavage Activity

**Figure 3:**
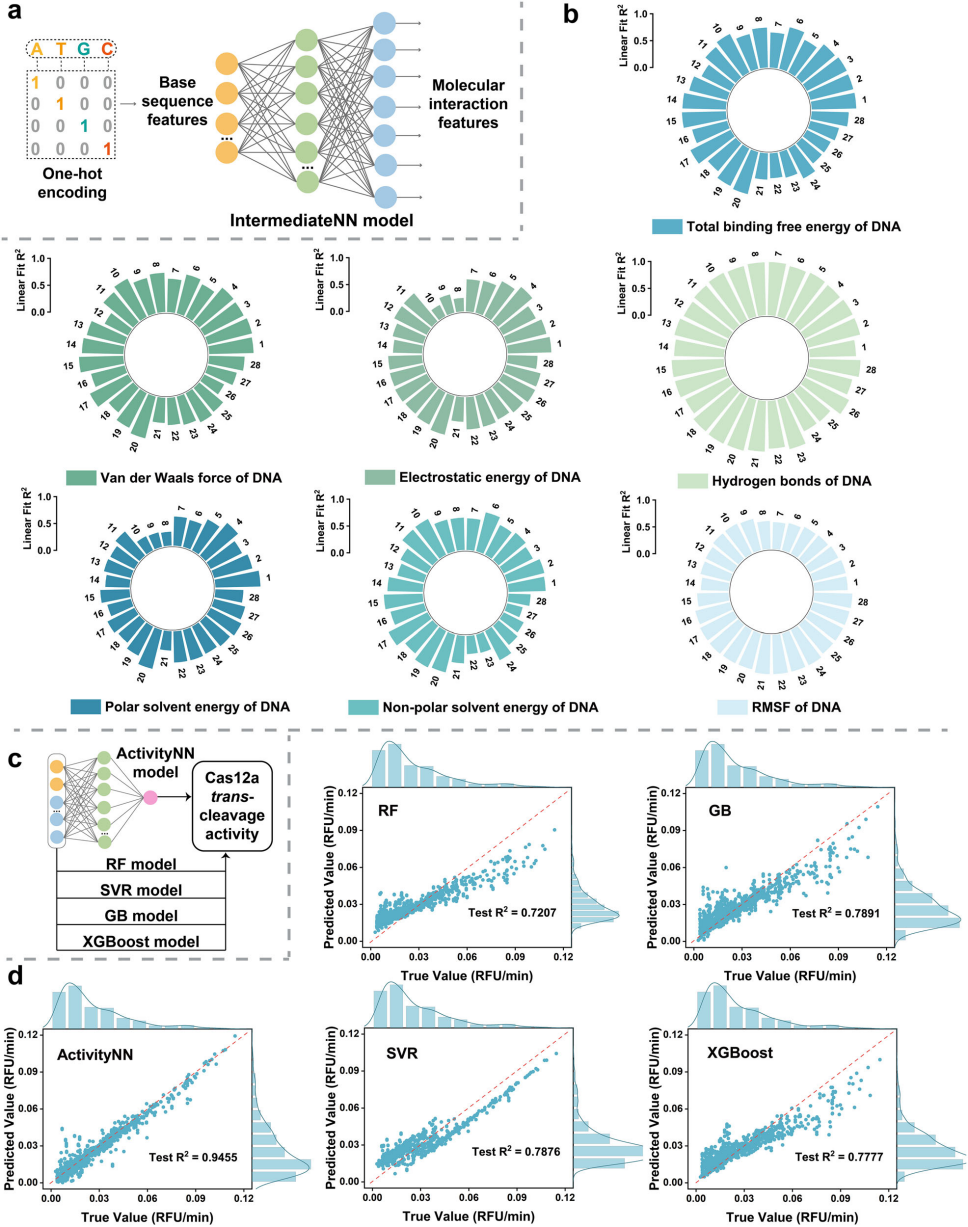
Models for predicting molecular interaction features of DNA and Cas12a *trans*‐cleavage activity. a) Diagram of the IntermediateNN model, a deep learning neural network built to predict seven molecular interaction features of DNA in the CRISPR‐Cas12a system, including total binding free energies, van der Waals forces, polar solvent energies, non‐polar solvent energies, electrostatic energies, hydrogen bond counts, and RMSF based on base sequence position 1–28. b) Prediction performance of the IntermediateNN model, shown by R^2^ values, indicating prediction accuracy for molecular interaction features across base positions. c) Comparison of the deep learning and machine learning models for predicting Cas12a *trans*‐cleavage activity, using an enriched dataset containing 28 base sequence features and 196 additional molecular interaction features at positions 1–28. d) Prediction performance of one deep learning model (ActivityNN) and four machine learning models (RF, SVR, GB, and XGBoost) by linear fitting the predicted and actual Cas12a *trans*‐cleavage activity.

Performing MD simulations typically takes significant computational resources and time (about 22 h per structure in this study, see Experimental section).^[^ ^27^ ^]^ To reduce the substantial computational resources and time of MD simulations, we first built a deep learning neural network model (IntermediateNN) for directly predicting seven molecular interaction features of DNA in the CRISPR‐Cas12a system according to base sequence position 1–28, including total binding free energies, van der Waals forces, polar solvent energies, non‐polar solvent energies, electrostatic energies, hydrogen bond counts, and RMSF (**Figure** **3**a). For developing the IntermediateNN model, we constructed the dataset including 180 target sequences that were used for MD simulations above, and divided into a training dataset (80%) and a test dataset (20%). The required calculation time for the IntermediateNN model on an unknown target was under 3 min, that was much faster than ordinary simulations (22 h). By analyzing the R^2^ value (a coefficient representing prediction performances), the predicted values of most features were comparable with the true value at all base positions, especially the hydrogen bond counts (Figure 3b). The R^2^ value of the hydrogen bond count was greater than 0.901 at more than 23 base locations, the maximum value was 0.992 at position 3 (R^2^ value closer to 1 indicates higher prediction accuracy). Additionally, for the prediction of total binding free energies, the R^2^ value was greater than 0.726 at more than 14 base locations, and the maximum value was 0.836 at position 17. Moreover, the R^2^ maximum values of van der Waals forces, electrostatic energies, polar solvent energies, non‐polar solvent energies, and RMSF were 0.847, 0.834, 0.837, 0.809, and 0.730, respectively. Based on these, the IntermediateNN model may be deemed as an effective method to reduce the time and computer resources needed for MD simulations to predict molecular interaction features of DNA in the CRISPR‐Cas12a system.

To predict the Cas12a *trans*‐cleavage activity, we introduced the molecular interaction features of DNA into the Cas12a *trans*‐cleavage activity dataset. Specifically, in addition to 180 target sequences for MD simulations, it also included an additional 1268 target sequences from a recent study (namely, a total of 1448 target sequences).^[^ ^13^ ^]^ Given the strong predictive ability of the IntermediateNN model, we input these 1268 additional sequences into the IntermediateNN model to acquire the corresponding molecular interaction features. We then merged these features with the original 180 sequences and their molecular interaction features. The merged dataset was divided into a training dataset (80%) and a test dataset (20%). Then, we compared the prediction performances of one deep learning neural network model (ActivityNN) and four machine learning models (random forest (RF), support vector regression (SVR), gradient boosting (GB), and extreme gradient boosting (XGBoost)) (Figure 3c). Compared with conventional datasets based only on base sequence features, the improved dataset contained extra 196 feature items (namely, seven molecular interaction features (RMSF, hydrogen bonds, total binding free energies, van der Waals forces, polar solvent energies, non‐polar solvent energies, and electrostatic energies) at the base position 1–28). Using the test dataset, the prediction performance of ActivityNN (*R* ^2^ = 0.9455) was better than RF (R^2^ = 0.7207), SVR (R^2^ = 0.7876), GB (R^2^ = 0.7891), and XGBoost (R^2^ = 0.7777) (Figure 3d), indicating that the ActivityNN model had good generalization capabilities with potentials for predicting the Cas12a *trans*‐cleavage activity. Moreover, the ActivityNN model also outperformed other existing models reported in the literatures (e.g., ADAPT,^[^ ^12^ ^]^ EasyDesign,^[^ ^13^ ^]^ △G‐ssDNA,^[^ ^20^ ^]^ and △G‐dsDNA^[^ ^20^ ^]^) (Figure S15, Supporting Information). Consequently, the ActivityNN model could be regarded as a new way to further screen more effective crRNA from various targets to improve the Cas12a *trans*‐cleavage activity.

### 2.5 Key Sequence Features Related with the Cas12a Trans‐Cleavage Activity

**Figure 4:**
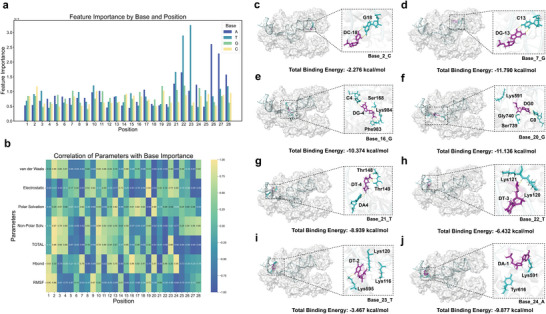
Key sequence features influencing Cas12a *trans*‐cleavage activity. a) PFI analysis for the ActivityNN model, showing the ranking of base importance across positions 1–28. Bases at positions 21, 22, and 23 on the PAM sequence and position 26, 27, and 28 on the PAM complementary sequence were found to have higher importance for Cas12a *trans*‐cleavage activity compared to bases on the target sequence. b) Correlation analysis between base importance and seven molecular interaction features (van der Waals forces, electrostatic energies, polar solvent energies, non‐polar solvent energies, total binding free energies, hydrogen bond counts, and RMSF) across positions 1–28 of the TS and PAM sequences. c–j) Schematic diagram of hydrogen bond interactions for the most important bases at specific positions.

We used permutation feature importance (PFI) analysis for the AcitivityNN model to investigate key sequence features related with the Cas12a *trans*‐cleavage activity. Identifying such features would facilitate crRNA screening and Cas12a‐based nucleic acid detection. Sorting by the base position and base type, we obtained the order of base importance for each position (**Figure** **4**a). Notably, the bases at position 21, 22, 23 on the PAM sequence and position 26, 27, 28 on the PAM complementary sequence showed higher importances on Cas12a *trans*‐cleavage activity than those on the target sequence. Across the base position (position 1–28), we constructed correlations between the base importance and seven molecular interaction features (van der Waals forces, electrostatic energies, polar solvent energies, non‐polar solvent energies, total binding free energies, hydrogen bonds, and RMSF) (Figure 4b). Results showed that at positions 2, 7, 16, 20 of the TS sequence, 21, 22, 23, 24 of the PAM sequence and 25, 26, 27, 28 of the PAM complementary sequence, most of the molecular interaction features showed extreme positive or negative correlation with the base importance, and studying their effects on these locations may help to understand their biological role on the Cas12a *trans*‐cleavage activity.

Specifically, at position 2 on the TS (PAM distal) sequence, the Pearson’ correlation coefficient (*r*) value between total binding free energies and the base importance was 0.962, while the *r*‐value between RMSF and the base importance was 0.858. According to the base importance result, the C base was identified as the most important at position 2. The average total binding energies of the C base (Base_2_C) was −2.276 kcal mol^−1^, and the average RMSF was 0.292 nm (Figure 2b,h), indicating that the structure around Base_2_C was fluctuated to conducive to Cas12a *trans*‐cleavage. The hydrogen bonds of Base_2_C were mainly formed by C18 (crRNA) (Figure 4c). Furthermore, at position 7, multiple molecular interaction features showed a significant negative correlation with base importance, especially with total binding energies (*r* = −0.894) and electrostatic energies (*r* = −0.906). The G base at position 7 had the highest importance. The average total binding energies of the G base (Base_7_G) was −11.790 kcal/mol, and the average electrostatic energies was 20.148 kcal mol^−1^ (Figure 2b,d), suggesting that the structural stability of Base_7_G by reducing binding energies and electrostatic energies would facilitate Cas12a *trans*‐cleavage. The hydrogen bonds of Base_7_G were mainly formed by the G13 (crRNA) (Figure 4d). Besides, the base importance at position 16 was strongly correlated with the corresponding hydrogen bonds (*r* = 0.977) and electrostatic energies (*r* = −0.906). The importance of G base (Base_16_G) at this location was the highest, the average hydrogen bond count was 599, and the average electrostatic energy was 10.480 kcal/mol (Figure 2d,g). Therefore, the Cas12a *trans*‐cleavage may be facilitated by enhancing hydrogen bonds and decreasing electrostatic energies of Base_16_G to keep structural stability. The hydrogen bonds of Base_16_G were mainly formed by the Ser168, Phe983, Lys984, and C4 (crRNA) with (Figure 4e). Additionally, at position 20 on the TS (PAM proximal) sequence, more than 5 molecular interaction features showed a significant correlation with base importance, especially with polar solvent energies (*r* = 0.884), electrostatic energies (*r* = −0.934), and total binding free energies (*r* = −0.915). The G base (Base_20_G) at position 20 was the most important. The average polar solvent energy was −5.897 kcal mol^−1^. In addition, the average electrostatic energy was 5.314 kcal mol^−1^ and the total binding energies was −11.136 kcal mol^−1^ (Figure 2b,d). These results demonstrated that Base_20_G could provide important stability to facilitate the location of crRNA by improving polar interaction. Hydrogen bonds of Base_20_G were mainly formed by Lys591, Ser739, Gly740, C0 (crRNA) (Figure 4f).

For the PAM region (position 21–24), the base importance at position 21 was negatively correlated with hydrogen bonds (*r* = ‐0.951). The T base (Base_21_T) at position 21 was the most important, the average hydrogen bond count was 386, and the average RMSF was 0.450 nm (Figure 2g,h). The hydrogen bonds of Base_21_T were mainly formed by Thr148, Thr149, and Gly146 (Figure 4g). Moreover, the base importance at position 22 had a strong negative correlation with the van der Waals forces (*r* = −0.819), and total binding energies (*r* = −0.951). The T base (Base_22_T) was the most important, the average van der Waals forces and binding energy were −4.788 and −6.432 kcal mol^−1^, respectively (Figure 2b,c). The hydrogen bonds of Base_22_T were mainly formed by Lys121 and Lys120 (Figure 4h). Additionally, the base importance at position 23 was positively correlated with the total binding free energy (*r* = 0.857). For the T base (Base_23_T) at this location, the average binding free energy was −3.467 kcal mol^−1^, the average hydrogen bond count was 123, and the average RMSF was 0.464 nm (Figure 2g,h). The hydrogen bonds of Base_23_T were mainly formed by Lys595, Lys120, and Lys116 (Figure 4i). For base at position 24, the base importance showed a strong correlation with more than 4 molecular interaction features, including the van der Waals force (*r* = −0.826), non‐polar solvent energy (*r* = −0.769), total binding free energy (*r* = −0.863), and hydrogen bond (*r* = 0.949). The average hydrogen bond count was 49 for the A base (Base_24_A) and 32 for the T base (Base_24_T). In addition, the average RMSF of the A base was 0.547, while the average RMSF of the T base was 1.023 nm (Figure 2h), indicating that the T base at this position may cause unfavorable structural fluctuations to reduce Cas12a *trans*‐cleavage activity. The hydrogen bonds of Base_24_A were mainly formed by Lys591 and Tyr616 (Figure 4j). Based on these, we further conducted the interaction analysis between the complementary strand of PAM (5’‐TTTA‐3’) and amino acids. Notably, the significantly low binding free energies were found between the complementary DNA bases (Base_21_T, Base_22_T, Base_23_T) and Lys595 (Figure S16a, Supporting Information). Specifically, the average binding energies of the complementary DNA bases (DA4, DA3, DA2) were −9.033, −10.499, and −12.146 kcal mol^−1^, that were much lower than the binding energies of surrounding bases (DG5, DG6, DA7, DC8). Meanwhile, the average binding energy of Lys595 was −6.888 kcal mol^−1^, that was also lower than that of other amino acids within the same domain (such as Pro594, Leu593, Val596, Phe597, Phe598). Additionally, the low electrostatic energies were also found between complementary DNA bases (Base_20_C, Base_21_T, and Base_22_T) and Lys601 (Figure S16b, Supporting Information). Specifically, the average electrostatic energies of the complementary DNA bases (DG5, DA4, DA3) were −1.065, −2.122, and −0.635 kcal mol^−1^, while the surrounding bases showed positive electrostatic energy values (e.g., DT1, DA2, DG6, DA7, DC8). Meanwhile, the average electrostatic energy of Lys601 was −31.287 kcal mol^−1^, that was also much lower than other amino acids in the same domain (such as Phe598, Ser599, and Trp602).

### 2.6 Application of Developed Models in Library Construction and Cas12a‐Based Nucleic Acid Detection

**Figure 5:**
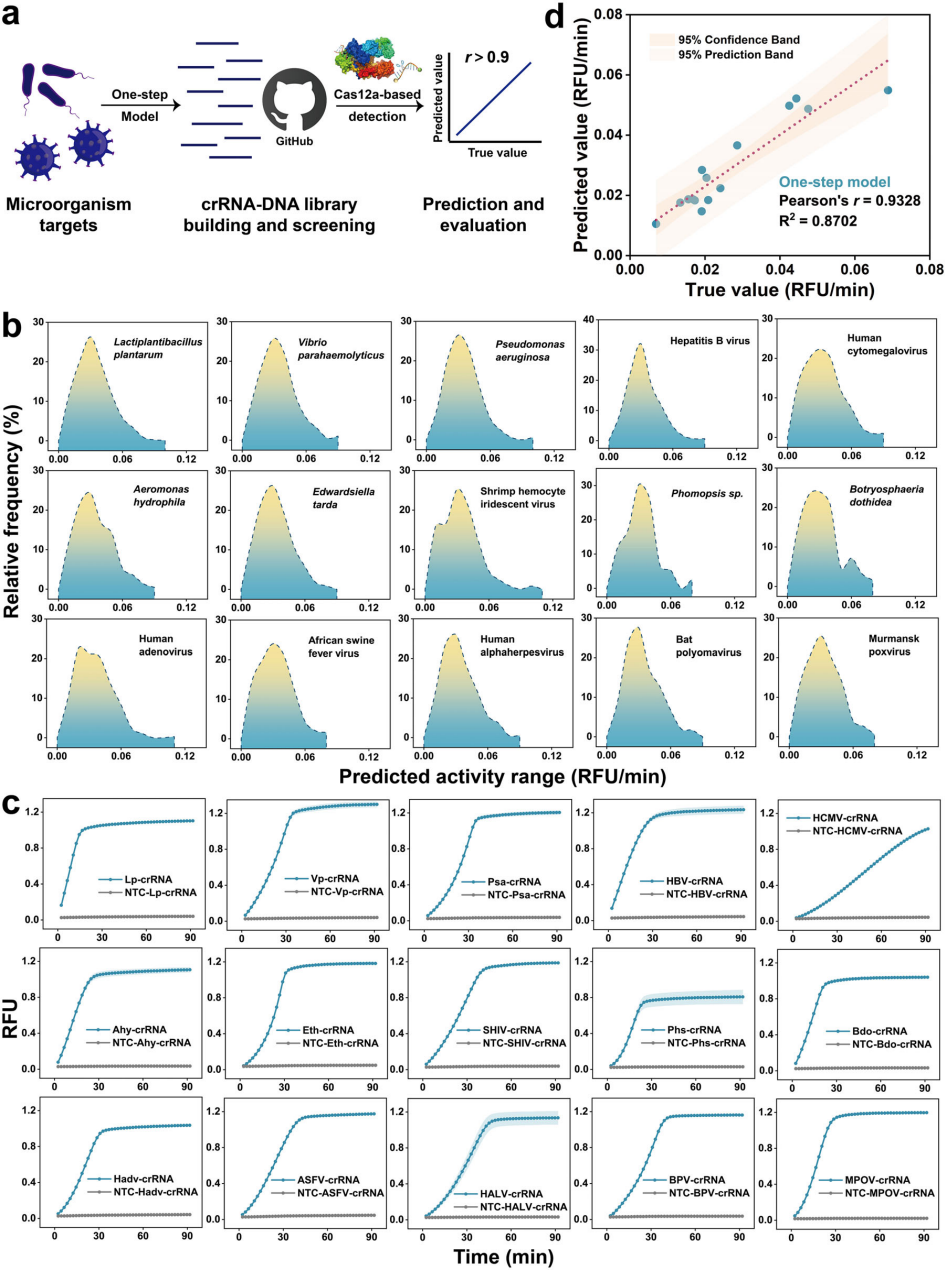
Application of developed models in Cas12a‐based nucleic acid detection. a) Schematic of the crRNA‐DNA library, built using the IntermediateNN and ActivityNN models, for screening crRNAs and predicting their Cas12a *trans*‐cleavage activity. The library includes feature sequences from various dsDNA viruses and reference targets, that is available online at https://github.com/zhyao‐JNU/crRNA‐DNA‐library. b) Distribution of Cas12a *trans*‐cleavage activity for crRNA counts screened from 15 reference targets, showing activity values ranging from 0 to 0.12 RFU/min. c) Experimental validation of 15 randomly synthesized crRNA‐DNA targets selected from the reference targets by using the real‐time fluorescence kinetics detection. All data were presented as mean ± SD (*n* = 3). d) Comparison between the predicted and experimentally verified Cas12a *trans*‐cleavage activity, indicating the accuracy of the models. RFU: relative fluorescence units.

To facilitate the crRNA availability and the practical application of developed models, we combined IntermediateNN with ActivityNN to construct the One‐step ActivityNN model. This is an end‐to‐end model starting from the input sequence features and directly outputting the Cas12a *trans*‐cleavage activity prediction value (Figure S17, Supporting Information). In this model, the molecular interaction features of DNA could be quickly calculated by IntermediateNN (in less than 3 min/structure) and automatically combined with the sequence features. Besides, we trained another end‐to‐end model to determine the contribution of the IntermediateNN to the ActivityNN by removing it and only retaining sequence features. Results showed that when only sequence features were used as input, the prediction performance was poor with R^2^ at 0.3537 (Figure S18, Supporting Information). These findings highlight the crucial contribution of the IntermediateNN model to the prediction accuracy. Then, we used the One‐step ActivityNN model to establish a crRNA‐DNA library containing abundant targets and the corresponding predicted Cas12a *trans*‐cleavage activity (**Figure** **5**a). The library could be freely available at an online website (https://github.com/zhyao‐JNU/crRNA‐DNA‐library), including 23 456 feature sequences of 436 representative dsDNA viruses at the family‐level (*Adenoviridae*, *Asfarviridae*, *Herpesvirales*, *Polyomaviridae*, and *Poxviridae*),^[^ ^28^ ^]^ and several reference targets (See Methods section, Figure S19, Supporting Information). Results showed that the activity range of screened crRNAs from 15 reference targets were widely distributed from 0 to 0.12 RFU/min (Figure 5b). We randomly synthesized 15 screened crRNA‐DNA targets for experimental testing to investigate the accuracy of prediction (Figure 5c). It can be found the predicted Cas12a *trans*‐cleavage activity from the One‐step ActivityNN model was in good agreement with that of the experimentally verified results (Pearson's *r* = 0.9328) (Figure 5d), suggesting that predicted results of the one‐step ActivityNN model could represent the Cas12a *trans*‐cleavage activity. Notably, the molecular interaction features were from the IntermediateNN model, that were not from MD simulations and may generate a gap when applying the One‐step ActivityNN model. Therefore, for 15 targets above, we compared the differences between the two methods (MD simulation and IntermediateNN model) to test whether the performance was weakened by this gap. Results indicated that the molecular interaction features derived from both methods were closely aligned, with Pearson's *r* ranging from 0.81 to 0.99 (Figure S20, Supporting Information). The correlation for polar solvation energy and electrostatic potential reached 0.99, demonstrating the strong predictive performance of the IntermediateNN model. Subsequently, we combined molecular interaction features from MD simulation with sequence features, and input them into the ActivityNN model for predicting the activity (Figure S17, Supporting Information). Results showed that the Pearson's *r* value for the method based on MD simulation (two‐step model) was 0.9429 (Figure S21, Supporting Information). Therefore, we believe that this gap had less impact on the predictive performance of the one‐step ActivityNN model.

Furthermore, the prediction models above were developed based on LbCas12a. To strengthen the generalizability across Cas12a subtypes, we performed MD simulations and experimental validation for AsCas12a (PDB ID: 5B43) using 15 representative targets above (Figure S22a, Supporting Information). Results showed that the DNA interaction features between LbCas12a and AsCas12a exhibited a strong correlation, with Pearson's *r* ranging from 0.76 to 0.89 (Figure S22b, Supporting Information). Additionally, the *trans*‐cleavage activity ranges of AsCas12a for these 15 targets were widely distributed from 0 to 0.08 RFU/min, that was comparable to the distribution of LbCas12a (Figure S22c,d, Supporting Information). By combining sequence features with interaction features of DNA from AsCas12a and inputting them into the ActivityNN model, we acquired the corresponding activity prediction value and compared them with the experimental value. The Pearson's *r* value between the prediction value and true value was 0.8812, indicating that the model maintained acceptable predictive power and adaptability across Cas12a subtypes (Figure S22e, Supporting Information).

## 3 Discussion

The CRISPR‐Cas12a system is emerging as a potential fast nucleic acid diagnostic tool due to its *trans*‐cleavage ability.^[^ ^29^ ^]^ The intramolecular and intermolecular interactions of the Cas12a/crRNA‐DNA complex can directly influence the CRISPR‐Cas12a *trans*‐cleavage activity. Here, we used the MD simulation technology to calculate the molecule interaction features of different Cas12a/crRNA‐DNA complexes from step 3 to step 4 (Figure S1, Supporting Information). In our comprehensive analysis of the molecular interaction features of DNA in the CRISPR‐Cas12a system, we observed significant differences of total binding free energies, van der Waals forces, non‐polar solvent energies, and hydrogen bond counts on the different base positions and types. G/C bases in the target sequence (positions 1–20) significantly contributed to structural stability through higher hydrogen bond counts compared to A/T bases. This was crucial for effective binding of Cas12a. In contrast, the PAM sequence (positions 21–24) and PAM complementary sequence (position 25–28) showed a balanced distribution of hydrogen bonds across all bases (A, T, G, C), suggesting that even PAM‐free sequences may maintain enzymatic activity of Cas12a in some ways.^[^ ^17^ ^]^ Notably, the G base at most positions demonstrated the lowest binding free energies and van der Waals forces, highlighting its critical role in stabilizing the complex. Conversely, the T base at position 24 (Base_24_T) was likely the least stable and least desirable, potentially compromising the integrity of the complex. This highlights the importance of base selection in specific positions for optimizing the binding stability and flexibility of the Cas12/crRNA‐DNA complex and suggests that certain bases may have enhanced structural roles depending on their location within the sequences.

Furthermore, the interaction features such as electrostatic energies, polar solvent energies, and RMSF highlight distinct stability and flexibility patterns at different base positions. Positions 7, 8, 9, and 10 on the TS may be crucial for effective target binding. In contrast, positions 21, 22, 23, and 24 located on PAM sequences may be more flexible regions. The difference of bases at certain key locations can significantly affect the stability and flexibility of the CRISPR‐Cas12a complex. The stable binding of target (position 1–20) can improve the recognition accuracy between Cas12a/crRNA and target DNA to reduce off‐target effects. Meanwhile, PAM region (position 21–24) may structurally provide better binding “scaffolding” or a more flexible structure, thereby improving the adaptability of the Cas12a complex to different target sequences.^[^ ^19^ ^]^ This knowledge would be a groundwork for understanding differences of Cas12a *trans*‐cleavage activity.

Moreover, the high R^2^ values of IntermediateNN model, particularly for hydrogen bonds and binding energies, suggested that it could effectively reduce the time and computational resources typically required for MD simulations. By combining molecular interaction features with base sequence features, the ActivityNN model was effectively developed to predict the Cas12a *trans*‐cleavage activity. The great Cas12a *trans*‐cleavage activity prediction performance of ActivityNN model (R^2^ = 0.9455) demonstrated its ability to screen suitable crRNAs on a wider range of targets. More importantly, the permutation feature importance (PFI) analysis of the ActivityNN model further provided an in‐depth analysis of base importance and molecular interactions related to the Cas12a *trans*‐cleavage activity. The PFI analysis highlighted the critical roles of specific bases at both the target and PAM sequences in affecting Cas12a *trans*‐cleavage activity. Notably, the bases at positions 21, 22, 23 on the PAM sequence and position 26, 27, 28 on the PAM complementary sequence were found to have a greater impact on *trans*‐cleavage activity than those on the target sequence. This finding was similar to previous reports that the PAM sequence can affect the on‐target *cis*‐cleavage activities.^[^ ^16^ ^]^ These results indicated that Cas12a *trans*‐cleavage activity was closely related to its on‐target cleavage activity. In addition, the G and T bases showed significant contributions through interactions like hydrogen bonding and electrostatic forces, especially in positions like 2, 7, 16, and 20 on the target sequence, as well as 21–23 on the PAM sequence, that provides key insights into designing optimal target and PAM sequences for enhanced the Cas12a *trans*‐cleavage activity. Also, the analysis of these key base‐related amino acid residues may serve as key residues for Cas12a protein modification engineering to improve enzyme activity, such as Thr148 and Thr149 at position 21, Lys121 at position 22, Lys595 at position 23. As reported in a recent study,^[^ ^17^ ^]^ the side chain of Lys595 was less ordered and showed weaker interactions with the TCTA, TCCA, and CCCA complexes than the TTTA complex. In this aspect, the significantly low binding free energies between the complementary DNA bases (Base_21_T, Base_22_T, Base_23_T) and Lys595 showed a strong binding affinity, that may significantly influence Cas12a *trans*‐cleavage activity. Besides, the negative values of the electrostatic energies indicated attractive interactions between the amino acid and the complementary DNA bases. Thus, Lys601 has significant electrostatic attraction to these complementary DNA bases (Base_20_C, Base_21_T, and Base_22_T), further reinforcing their binding affinity. Beyond the binding affinities, these results indicated that the interactions were likely contributing significantly to the overall stability of the Cas12a/crRNA‐DNA complex and may play an important role in affecting the *trans*‐cleavage activity and the specificity of the Cas12a enzyme. These findings can aid in the refinement of crRNA sequences for more efficient Cas12a‐based nucleic acid detection systems.

Additionally, the established crRNA‐DNA library including more than 23 456 feature sequences highlighted the practical application and effectiveness, that is freely available online, making it a useful tool for researchers working in diagnostics. By searching the library, the Cas12a *trans*‐cleavage activity prediction results of the corresponding viral or bacterial targets can be obtained directly, that can help in the development of effective CRISPR‐based diagnostic and therapeutic systems for both viral and bacterial infections.

Besides, in our current study, we developed the prediction model based on LbCas12a, as it is one of the most widely used Cas12a subtypes in CRISPR‐based in vitro diagnostic assays.^[^ ^4^, ^5^, ^6^, ^26^ ^]^ Another reason for selecting LbCas12a was its relatively low background signal compared to other Cas12a subtypes. For instance, a recent study demonstrated that when AsCas12a was used, all tested crRNAs exhibited higher background fluorescence, whereas LbCas12a resulted in much lower background signals, highlighting its superior suitability for diagnostic applications.^[^ ^20^ ^]^ As for other Cas12a subtypes, the sequence homology and similarity were found in several Cas12a subtypes, including LbCas12a, FnCas12a, AsCas12a, and FtCas12a.^[^ ^18^ ^]^ Specifically, the protein sequence identity and similarity of LbCas12a:FnCas12a was 98%:96%, FnCas12a:AsCas12a was 72%:84%, and FnCas12a:FtCas12a was 99%:100%. These data suggested that different Cas12a subtypes may be functionally similar. We fully acknowledge that while different Cas12a subtypes may show functional similarities, they could also present functional differences. Therefore, we investigated the performance of our model for other subtypes such as AsCas12a and made relevant comparisons. Given the high structural similarity of the two subtypes, the calculated molecular interaction features (energies, hydrogen bonds, RMSF) of DNA for LbCas12a and AsCas12a were highly correlated, with Pearson's *r* ranging from 0.76 to 0.89 (Figure S22). Based on this, the ActivityNN model still showed acceptable prediction performance when inputting interaction features of DNA from AsCas12a. These findings suggested that the model, originally developed for LbCas12a, retained its predictive power and provides valuable insights into the potential of other Cas12a subtypes. Nonetheless, for further improvement in the model's prediction accuracy, additional datasets related to *trans*‐cleavage activity for other subtypes, such as AsCas12a, will be necessary. This will enable more precise adjustments and refinements to the model, ensuring even broader applicability across different Cas12a subtypes.

Lastly, although it is of great significance to investigate the effect of molecule interaction features on the Cas12a *trans*‐cleavage activity, only 100 ns MD simulations of the Cas12a/crRNA‐DNA complex were performed in this study. Future work may also need to examine longer simulation times (us‐ level or even ms‐ level) to approximate the true Cas12a *trans*‐cleavage state.^[^ ^23^, ^27^ ^]^ In addition, the data size of this study was still limited, and more datasets and advanced neural network models could be used for training to further improve the accuracy of predictions.^[^ ^13^, ^30^, ^31^ ^]^ Finally, this study was mainly based on computer verification, supplemented by experimental verification. Subsequent large‐scale experimental verification of crRNAs of bacteria, fungi, and viruses in medical diagnosis, food safety, and environmental monitoring will bolster the credibility of the results,^[^ ^32^, ^33^ ^]^ that needs to be better applied in practice in the future.

## 4 Conclusion

This study presented a computational framework that enhanced the prediction of Cas12a *trans*‐cleavage activity by integrating molecular dynamics simulations with neural network models. Our approach improved prediction accuracy by considering molecular interaction features, identifying key sequence elements that influence Cas12a *trans*‐cleavage activity. Validation with reference targets from a crRNA‐DNA library demonstrated high predictive performance, offering a powerful tool for optimizing crRNA design and enhancing CRISPR/Cas12a applications in rapid nucleic acid diagnostics.

## 5 Experimental Section

### Construction of crRNA‐DNA Dataset and Determination of Cas12a Trans‐Cleavage Activity

Various microorganisms were chosen as research targets from food processing, food safety, and medical diagnostics, such as *L. acetotolerans*, *L. plantarum, P. kudriazevii*, *S. eriocheiris*, white spot syndrome virus, *S*. *typhimurium*, and SARS‐CoV‐2. First, 60 targets (Group 1) were randomly designed, including 20 PAM‐included (TTTN, N = A/T/C/G) crRNAs and 40 PAM‐free crRNAs based on several species‐specific genes of these microorganisms. Moreover, as previously reported,^[^ ^26^ ^]^ another 4 targets were also selected from spacers 4 and 5 of the Orf1ab gene, spacer 2 of Spike (S) gene of SARS‐CoV‐2 and spacer 1 of the human papillomavirus type 18 (HPV18) L1 gene. Then, the 4 spacers were further point‐mutated from TTTV (V = A/C/G) to VTTV, TVTV, or TTVV to produce 120 targets (Group 2). Furthermore, an additional 1268 target sequences were selected from a recent study (Group 3).^[^ ^13^ ^]^ These sequences were completely independent from the targets in Group 1&2 and had a uniform distribution of Cas12a *trans*‐cleavage activity values. Consequently, a total of 1448 targets were obtained to construct the crRNA‐DNA dataset (Tables S1–S3, Supporting Information). All oligonucleotide sequences were synthesized from Sangon Biotech Co., Ltd. (Shanghai, China), purified by HPLC, and quantified by a NanoDrop One spectrophotometer (Thermo Fisher).

The Cas12a *trans*‐cleavage activity of 60 targets (Group 1) and 4 targets (Group 2) was then determined. The 4 targets included Orf1ab spacer 4 (PAM: GTTG), Orf1ab spacer 5 (PAM: CTTA), S gene spacer 2 (PAM: TTCA), and HPV18 L1 gene spacer 1 (PAM: TTAC). The assay was conducted in reaction mixtures including 100 nm LbCas12a (Cat. No. 32108, Tolobio, Shanghai, China), 100 nm crRNA, 400 nm fluorophore and quencher labeled single strand DNA (ssDNA‐FQ) reporter, and 3.5 nm dsDNA targets. Then, as previously reported,^[^ ^26^ ^]^ the Cas12a *trans*‐cleavage activity of these 4 targets (Group 2) were determined in another reaction condition including 33 nm LbCas12a, 33 nm crRNA, 400 nm reporter (5’‐FAM‐TTATT‐BHQ1‐3’) and 3.5 nm dsDNA targets. For targets in Group 3, three target sequences with high, medium, and low Cas12a *trans*‐cleavage activity were synthesized and performed real‐time fluorescence kinetic assays at two reaction conditions, including 1) 200 ng LbCas12a, 200 ng crRNA, 400 nm reporter and 3.5 nm dsDNA targets, and 2) 100 nm LbCas12a, 100 nm crRNA, 400 nm reporter and 3.5 nm dsDNA targets. Subsequently, the *trans*‐cleavage activity of 1448 targets was acquired by normalizing the data reported in the previous study.^[^ ^13^, ^26^ ^]^ The dsDNA targets were synthesized by annealing two complementary oligonucleotides (Tables S2 and S3, Supporting Information). The *trans*‐cleavage assays were conducted in 1 × NEBuffer 2.1 buffer at 37 °C. The fluorescence signals were monitored through the Light Cycler 96 Instrument (Roche).

In this study, the ssDNA‐FQ reporter could be *trans*‐cleaved by Cas12a/crRNA‐dsDNA complex to release fluorescence signals over time and the cleavage follows first‐order kinetics, as previously reported:^[^ ^4^, ^12^, ^34^ ^]^

(1) $$-\frac{kcat}{KM}\left[E\right]\;\left[R\right]=\frac{d\left[R\right]}{dT}\;$$

(2)

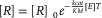

where [*R*] represents the not‐yet‐cleaved reporter, [*E*] represents the concentration of the Cas12a/crRNA‐dsDNA complex, kcat/KM represents the catalytic activity of the Cas12a complex and *T* represents time. The fluorescence signals (*F*) are proportional to the concentration of cleaved reporters at some time point:

(3) $$F\propto {\left[R\right]}_{0}-\left[R\right]$$

For each Cas12a/crRNA‐dsDNA complex, a curve of the form could be fitted:

(4) $$F=P\left(1-e^{-kt}\right)+N$$

where *P* represents the peak value of fluorescence, and *N* represents the background fluorescence. *k* is the Cas12a *trans*‐cleavage activity. It is proportional to the catalytic activity of the CRISPR complex:

(5) $$k=\frac{kcat}{KM}\;\left[E\right]$$

Therefore, when the concentration ([*E*]) of Cas12a/crRNA was kept constant, the Cas12a *trans*‐cleavage activity could be simply defined as follows:^[^ ^5^, ^35^ ^]^

(6) $$k=\frac{dF}{dT}\;$$

where *F* stands for relative fluorescence units (RFU), and *T* stands for reaction time. To obtain the Cas12a *trans*‐cleavage activity dataset, the increased fluorescence signals from the initial 10 min were collected and the corresponding *k* value of different crRNAs was calculated.

### Molecular Dynamics Simulations and Molecular Interaction Features Calculation

The crRNAs were used above to calculate the Cas12a/crRNA‐DNA interaction features. The original structure was from a high‐resolution crystal structure of the Cas12a/crRNA‐DNA ternary complex without PAM‐distal dsDNA (PDB ID: 5XUS) (Figures S1 and S2, Supporting Information). The web 3DNA platform (http://web.x3dna.org/) was used to mutate nucleic acid base to construct a total of 180 Cas12a/crRNA‐DNA complexes for MD simulations. The all‐atom Cas12a/crRNA‐DNA structures were solvated in TIP3P explicit water, and Na^+^ and Cl^−^ ions were added to neutralize the reaction systems. The amber14sb_OL15 force field was adopted for all structures. All MD simulations were performed with the GROMACS 2023.2 package. The systems were prepared by energy minimization, followed by 100 ps NVT and 100 ps NPT simulations with the Cas12a/crRNA‐DNA structures fixed. The production simulations were performed at 310 K for 100 ns. The simulation time step was set to 2 fs, and the simulation trajectories were collected every 10 ps. Electrostatic interactions were calculated using the PME algorithm, with a cutoff radius of 1.0 nm. The temperature coupling was calculated using a V‐rescale, with a coupling time of 0.1 ps. Lincs constraints were used to all bonds. The MD simulations were performed using RTX 4090 (GPU) supported by the high‐performance cluster platform of the School of Biotechnology at Jiangnan University. The computational time for a single production run was about 22 hours, and the total computational time for all simulation systems was about 11 880 h (180 systems × 3 replicates × 22 h).

Seven molecular interaction features were collected according to the 28 base locations from 180 Cas12a/crRNA‐DNA complexes by MD simulations, including RMSF, hydrogen bonds, total binding free energies, van der Waals force, polar solvent energies, non‐polar solvent energies, and electrostatic energies. A total of 196 (28×7) features were acquired. The trajectories corresponding to 90–100 ns in the MD simulations were chosen to calculate molecular interaction features of DNA in the CRISPR‐Cas12a system. Specifically, the GROMACS package was used to calculate hydrogen bonds and root mean square deviation (RMSF). To calculate the total binding free energies and free energy decompositions (van der Waals force, polar solvent energies, non‐polar solvent energies, and electrostatic energies) of the Cas12a/crRNA‐DNA structures, the Molecular mechanics/Generalized‐Born Surface Area (MM‐GBSA) method of gmx_MMPBSA program (a GROMACS tool based on AMBER's binding free energy calculation engine) was used for free energy calculation of GROMACS files.

### Modeling to Predict Molecular Interaction Features and Cas12a Trans‐Cleavage Activity

Seven molecular interaction features of DNA in the CRISPR‐Cas12a system were used above as prediction goals to construct a deep learning neural network model (IntermediateNN). The IntermediateNN was trained by using 28‐nt sequences including 20‐nt specific sequences on the TS (5’–3’), 4‐nt PAM sequence on the NTS (5’–3’), and 4‐nt PAM complementary sequence on the TS (5’–3’). The 4‐bit one‐hot encoding was used to encode each base (i.e., A, T, G, and C) of the 28‐nt sequences. Namely, the 4 types of bases were encoded as binary matrices (‘A’: [1, 0, 0, 0], ‘T’: [0, 1, 0, 0], ‘G’: [0, 0, 1, 0], ‘G’: [0, 0, 0, 1]). The dataset included 180 target sequences in Groups 1&2. The dataset was divided into a training dataset (80%) and a test dataset (20%). The polynomial features of bases (quadratic terms and product terms, a total of 6441 feature terms) were calculated to improve data diversity. Multiple fully connected layers and batch‐normalized layers were adopted to train the model. The dropout technology was used to prevent overfitting and nonlinearity was introduced through the Rectified Linear Unit (ReLU) activation function. The hyperparameters of the model were set, including hidden layer units (910), dropout rate (0.3087), learning rate (0.000733), and batch size (30). The normalized features and target variables were then converted to the tensors of PyTorch. Moreover, early stops were also used to prevent overfitting during the training process.

The molecular interaction features from MD simulations were combined with base features (one‐hot encoding) to form a new dataset, aiming to establish a model predicting the Cas12a *trans*‐cleavage activity. The dataset contained 225 features (including one GC‐content feature, 28 base features of 1448 target sequences in Group 1&2&3, and 196 molecular interaction features) and was split into a training dataset (80%) and a test dataset (20%). Four machine learning models (RF, SVR, GB, and XGBoost) and one PyTorch deep learning neural network model (ActivityNN) were established. The RF, SVR, GB, and XGBoost models were hyperparameter optimized. For the RF model, it is an integrated algorithm based on multiple decision trees with good anti‐overfitting ability. The hyperparameters of RF included the number of trees n_estimators (100, 200, 300) and the depth of the tree max_depth (0, 10, 20, 30). For the SVR model, it works well when dealing with small datasets or high‐dimensional data, different kernel functions (‘linear,’ ‘poly,’ ‘rbf’) and penalty coefficients C (0.1, 1, 10) were tested. For the GB model, it can efficiently capture complex nonlinear relationships by integrating multiple weak learners. The hyperparameters of GB included the number of trees n_estimators (100, 200, 300), the learning rate (0.01, 0.1, 0.2), and the depth of the tree max_depth (3, 4, 5). For the XGBoost model, it could further improve training efficiency by introducing regularization to control model complexity and support for GPU acceleration. The hyperparameters of XGBoost mainly included the n_estimators (100, 200, 300) and the depth of the tree max_depth (3, 4, 5). For the ActivityNN model, the number of neurons in the hidden layer was 256. The hidden layer passed the input data to a high‐dimensional space through the fully connected layer (Linear layer). The nonlinearity was then introduced by the ReLU activation function. The dropout layer was introduced to reduce the overfitted of the ActivityNN model, and the dropout rate was set to 0.3.

### Feature Importance Analysis

The feature importance of the ActivityNN model was calculated by the permutation feature importance (PFI) algorithm to quantify the contribution of each feature to the model performance. Permutation feature importance measures the contribution of a feature by randomly shuffling the value of that feature and observing how much the model's performance degrades. Each feature was randomly replaced ten times to ensure the stability of the results. The order of base importance for each position was calculated according to the base position and base type, and established correlations between the seven molecular interaction features to investigate the key sequence features affecting the Cas12a *trans*‐cleavage activity.

### Experimental Evaluation of Developed Models

A crRNA‐DNA library was first constructed by targeting 436 representative dsDNA viruses including *Adenoviridae*, *Asfarviridae*, *Herpesvirales*, *Polyomaviridae*, and *Poxviridae*.^[^ ^28^ ^]^ According to the length of feature sequences from 200 to 3500 bp, a total of 2526 sequences of *Adenoviridae*, 1866 sequences of *Asfarviridae*, 8486 sequences of *Herpesvirales*, 752 sequences of *Polyomaviridae*, and 9826 sequences of *Poxviridae* were collected to acquired 23 456 feature sequences. Among them, 5 reference targets were selected for experimental verification, including Human adenovirus, African swine fever virus, Human alphaherpesvirus, Bat polyomavirus, and Murmansk poxvirus (Table S3, Supporting Information). Besides, other ten reference specific targets were also contained in this library for experimental verification, including *Lactiplantibacillus plantarum*, *Pseudomonas aeruginosa*, *Vibrio parahaemolyticus*, Hepatitis B virus, Human cytomegalovirus, *Aeromonas hydrophila*, *Edwardsiella tarda*, Shrimp hemocyte iridescent virus, *Phomopsis* sp., and *Botryosphaeria dothidea* (Table S4, Supporting Information).^[^ ^36^, ^37^, ^38^, ^39^, ^40^ ^]^ To facilitate the use of developed models, the IntermediateNN were combined with ActivityNN to construct the One‐step ActivityNN model, that is an end‐to‐end model starting from the input sequence features, then the molecular interaction features could be quickly calculated and automatically combined with the sequence features to directly output the Cas12a *trans*‐cleavage activity prediction value. For all the targets in the library, the One‐step ActivityNN model was used to predict the Cas12a *trans*‐cleavage activity. The complete library was uploaded to an online website and could be accessed at https://github.com/zhyao‐JNU/crRNA‐DNA‐library. Then, 15 crRNAs were randomly selected from 15 reference‐specific targets, and synthesized their short dsDNA targets (Table S5, Supporting Information) to perform fluorescence kinetic detection and compared the true *trans*‐cleavage activity with predicted activity. The fluorescence kinetic detection was also conducted in 100 nm LbCas12a, 100 nm crRNA, 400 nm reporter, and 3.5 nm dsDNA targets. Besides, the AsCas12a (PDB ID: 5B43) was used to verify generalizability of models above. The AsCas12a (Cat. No. 32104) was purchased from Tolobio Co., Ltd. (Shanghai, China). The experimental details of MD simulations and fluorescence kinetic detection assays were the same as the sections above.

### Statistics Analysis

A total of 1448 targets were used to build and test the ActivityNN model in this study and a random split of 1159 samples for training, 289 samples for test. All algorithms were imported in Python. Data was pre‐processed according to the workflow described above.

## Conflict of Interest

The authors declare no conflict of interest.

## Supporting information



Supporting Information

Supplemental Table 3

## Data Availability

The data that support the findings of this study are available from the corresponding author upon reasonable request.

## References

[1] J. S. Chen , E. Ma , L. B. Harrington , M. Da Costa , X. Tian , J. M. Palefsky , J. A. Doudna , Science 2018, 360, 436. 29449511 10.1126/science.aar6245 PMC6628903

[2] O. O. Abudayyeh , J. S. Gootenberg , Science 2021, 372, 914. 34045344 10.1126/science.abi9335

[3] Z. Yao , W. Li , K. He , H. Wang , Y. Xu , X. Xu , Q. Wu , L. Wang , Crit. Rev. Microbiol. 2024, 10.1080/1040841X.2024.2404041. 39287550

[4] D. A. Huyke , A. Ramachandran , V. I. Bashkirov , E. K. Kotseroglou , T. Kotseroglou , J. G. Santiago , Anal. Chem. 2022, 94, 9826. 35759403 10.1021/acs.analchem.2c01670

[5] C. Wu , Z. Yao , Q. Wu , Y. Xu , Food Biosci. 2024, 62, 105290.

[6] Z. Yao , K. He , H. Wang , S. Feng , X. Ding , Y. Xu , Q. Wang , X. Xu , Q. Wu , L. Wang , ACS Sens. 2024, 9, 3511. 38651662 10.1021/acssensors.3c02485

[7] S. Mantena , P. P. Pillai , B. A. Petros , N. L. Welch , C. Myhrvold , P. C. Sabeti , H. C. Metsky , Nat. Biotechnol. 2024, 10.1038/s41587-024-02422-w. PMC12439118 39394482

[8] L. T. Nguyen , B. M. Smith , P. K. Jain , Nat. Commun. 2020, 11, 4906. 32999292 10.1038/s41467-020-18615-1 PMC7528031

[9] J. Moon , C. Liu , Nat. Commun. 2023, 14, 7504. 37980404 10.1038/s41467-023-43389-7 PMC10657364

[10] Y. J. Jang , Q.‐Q. Qin , S.‐Y. Huang , A. T. J. Peter , X.‐M. Ding , B. Kornmann , Nat. Commun. 2024, 15, 6601. 39097570 10.1038/s41467-024-50955-0 PMC11297950

[11] H. K. Kim , S. Min , M. Song , S. Jung , J. W. Choi , Y. Kim , S. Lee , S. Yoon , H. Kim , Nat. Biotechnol. 2018, 36, 239. 29431740 10.1038/nbt.4061

[12] H. C. Metsky , N. L. Welch , P. P. Pillai , N. J. Haradhvala , L. Rumker , S. Mantena , Y. B. Zhang , D. K. Yang , C. M. Ackerman , J. Weller , P. C. Blainey , C. Myhrvold , M. Mitzenmacher , P. C. Sabeti , Nat. Biotechnol. 2022, 40, 1123. 35241837 10.1038/s41587-022-01213-5 PMC9287178

[13] B. Huang , L. Guo , H. Yin , Y. Wu , Z. Zeng , S. Xu , Y. Lou , Z. Ai , W. Zhang , X. Kan , Q. Yu , S. Du , C. Li , L. Wu , X. Huang , S. Wang , X. Wang , Imeta 2024, 3, 214. 10.1002/imt2.214 PMC11316927 39135699

[14] S. Stella , P. Mesa , J. Thomsen , B. Paul , P. Alcón , S. B. Jensen , B. Saligram , M. E. Moses , N. S. Hatzakis , G. Montoya , Cell 2018, 175, 1856. 30503205 10.1016/j.cell.2018.10.045

[15] D. C. Swarts , J. van der Oost , M. Jinek , Mol. Cell 2017, 66, 221. 28431230 10.1016/j.molcel.2017.03.016 PMC6879319

[16] D. C. Swarts , M. Jinek , Mol. Cell 2019, 73, 589. 30639240 10.1016/j.molcel.2018.11.021 PMC6858279

[17] T. Yamano , B. Zetsche , R. Ishitani , F. Zhang , H. Nishimasu , O. Nureki , Mol. Cell 2017, 67, 633. 28781234 10.1016/j.molcel.2017.06.035 PMC5957536

[18] S. Stella , P. Alcón , G. Montoya , Nature 2017, 546, 559. 28562584 10.1038/nature22398

[19] H. Yang , P. Gao , K. R. Rajashankar , D. J. Patel , Cell 2016, 167, 1814. 27984729 10.1016/j.cell.2016.11.053 PMC5278635

[20] J. Zhang , X. Guan , J. Moon , S. Zhang , Z. Jia , R. Yang , C. Hou , C. Guo , M. Pei , C. Liu , Nucleic Acids Res. 2024, 52, gkae1124. 10.1093/nar/gkae1124 PMC11662684 39588774

[21] Q. Chen , G. Chuai , H. Zhang , J. Tang , L. Duan , H. Guan , W. Li , W. Li , J. Wen , E. Zuo , Q. Zhang , Q. Liu , Nat. Commun. 2023, 14, 7521. 37980345 10.1038/s41467-023-42695-4 PMC10657421

[22] A. Saha , P. R. Arantes , R. V. Hsu , Y. B. Narkhede , M. Jinek , G. Palermo , J. Chem. Inf. Model. 2020, 60, 6427. 33107304 10.1021/acs.jcim.0c00929 PMC7605327

[23] A. Saha , M. Ahsan , P. R. Arantes , M. Schmitz , C. Chanez , M. Jinek , G. Palermo , Nat. Commun. 2024, 15, 1473. 38368461 10.1038/s41467-024-45762-6 PMC10874386

[24] Y. Chen , X. Xu , J. Wang , Y. Zhang , W. Zeng , Y. Liu , X. Zhang , Anal. Chem. 2022, 94, 9724. 35762828 10.1021/acs.analchem.2c01193

[25] L. Ma , L. Peng , L. J. Yin , G. Z. Liu , S. L. Man , ACS Sens. 2021, 6, 2920. 34281340 10.1021/acssensors.1c00686

[26] S. Lu , X. Tong , Y. Han , K. Zhang , Y. Zhang , Q. Chen , J. Duan , X. Lei , M. Huang , Y. Qiu , D.‐Y. Zhang , X. Zhou , Y. Zhang , H. Yin , Nat. Biomed. Eng. 2022, 6, 286. 35314803 10.1038/s41551-022-00861-x

[27] X. Song , L. Bao , C. Feng , Q. Huang , F. Zhang , X. Gao , R. Han , Nat. Commun. 2024, 15, 5538. 38956032 10.1038/s41467-024-49858-x PMC11219796

[28] R. D. Olson , R. Assaf , T. Brettin , N. Conrad , C. Cucinell , J. J. Davis , D. M. Dempsey , A. Dickerman , E. M. Dietrich , R. W. Kenyon , M. Kuscuoglu , E. J. Lefkowitz , J. Lu , D. Machi , C. Macken , C. Mao , A. Niewiadomska , M. Nguyen , G. J. Olsen , J. C. Overbeek , B. Parrello , V. Parrello , J. S. Porter , G. D. Pusch , M. Shukla , I. Singh , L. Stewart , G. Tan , C. Thomas , M. VanOeffelen , et al., Nucleic Acids Res. 2023, 51, D678. 36350631 10.1093/nar/gkac1003 PMC9825582

[29] X. Wu , X. Lou , H. Zhou , J. J. Raymond , L. G. Kwang , F. Y. T. Ong , S. L. Springs , H. Yu , Trends Food Sci. Technol. 2024, 145, 104349.

[30] J. Lin , Z. Zhang , S. Zhang , J. Chen , K. C. Wong , Adv. Sci. 2020, 7, 1903562.

[31] H. Zhang , J. Yan , Z. Lu , Y. Zhou , Q. Zhang , T. Cui , Y. Li , H. Chen , L. Ma , Cell Discovery 2023, 9, 48. 37193681 10.1038/s41421-023-00549-9 PMC10188485

[32] Z. Yao , Y. Zhu , Q. Wu , Y. Xu , Crit. Rev. Food Sci. Nutr. 2022, 64, 4995. 36412251 10.1080/10408398.2022.2147899

[33] W. Li , Z. Yao , T. Ma , Z. Ye , K. He , L. Wang , H. Wang , Y. Fu , X. Xu , Adv. Colloid Interface Sci. 2024, 332, 103276. 39146580 10.1016/j.cis.2024.103276

[34] A. Ramachandran , J. G. Santiago , Anal. Chem. 2021, 93, 7456. 33979119 10.1021/acs.analchem.1c00525

[35] Y. Y. Shan , X. M. Zhou , R. Huang , D. Xing , Anal. Chem. 2019, 91, 5278. 30873832 10.1021/acs.analchem.9b00073

[36] R. Du , S. Wang , Q. Wu , Y. Xu , Syst. Microbiol. Biomanufacturing 2023, 3, 593.

[37] X. Qiu , X. Liu , R. Wang , X. Ma , L. Han , J. Yao , Z. Li , Microbiol. Spectr. 2023, 11, 03523. 10.1128/spectrum.03523-22 PMC9927138 36622174

[38] X. Chen , L. Wang , F. He , G. Chen , L. Bai , K. He , F. Zhang , X. Xu , Anal. Chem. 2021, 93, 14300. 34645259 10.1021/acs.analchem.1c03468

[39] X. Zhang , Y. Tian , L. Xu , Z. Fan , Y. Cao , Y. Ma , H. Li , F. Ren , Hepatol. Int. 2022, 16, 306. 35298777 10.1007/s12072-022-10311-0 PMC9013339

[40] M. H. Liu , X. C. Guo , M. L. Sun , J. L. Li , S. H. Liu , Y. Z. Chen , D. Y. Wang , L. Wang , Y. Z. Li , J. Yao , Y. Li , Y. Q. Pan , Front. Cell. Infect. Microbiol. 2024, 14, 1430302. 39099883 10.3389/fcimb.2024.1430302 PMC11294213

