# Supplementary material for: Facilitating crRNA Design by Integrating DNA Interaction Features of CRISPR‐Cas12a System
Source: Adv Sci (Weinh). 2025 May 8;12(25):2501269. doi: 10.1002/advs.202501269 (PMC12224974; doi:10.1002/advs.202501269)
Supplement: Supplementary file 1 — Supporting Information [file ADVS-12-2501269-s002.docx]

Supporting Information

Facilitating crRNA design by integrating DNA interaction features of CRISPR-Cas12a system

*Zhihao Yao ^a,b^, Wanglu Li ^b^, Kaiyu He ^b^, Hongmei Wang ^b^, Yan Xu ^a^, Qun Wu ^a^*, Liu Wang ^b,c^*, Yao Nie ^a^**

^a^ The Key Laboratory of Industrial Biotechnology, Ministry of Education; State Key Laboratory of Food Science and Resources; School of Biotechnology, Jiangnan University, Wuxi, Jiangsu 214122, China

^b^ State Key Laboratory for Managing Biotic and Chemical Threats to the Quality and Safety of Agro-products; Institute of Agro-product Safety and Nutrition, Zhejiang Academy of Agricultural Sciences, Hangzhou 310021, China

^c^ Key Laboratory of Information Traceability for Agricultural Products, Ministry of Agriculture and Rural Affairs, Hangzhou 310021, China

*Corresponding authors: Yao Nie, E-mail: [ynie@jiangnan.edu.cn](mailto:ynie@jiangnan.edu.cn); Liu Wang, E-mail: [wangliually@126.com](mailto:wangliually@126.com); Qun Wu, E-mail: [wuq@jiangnan.edu.cn](mailto:wuq@jiangnan.edu.cn)


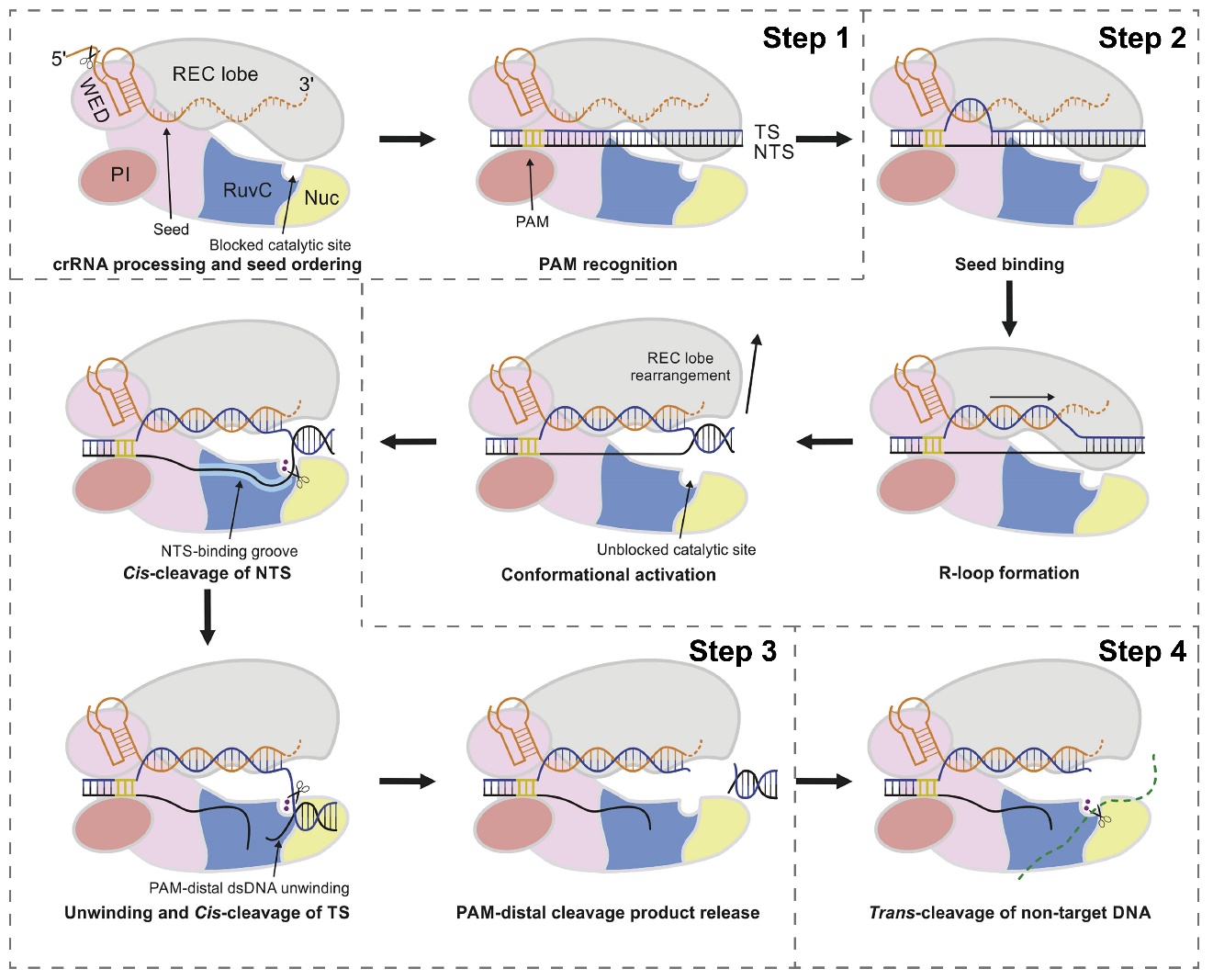


**Figure S1. Schematic illustration of the Cas12a *cis*- and *trans*-cleavage process, as previously reported^[1]^.** The whole reaction processes include four main steps: 1) recognition of the PAM by the WED and PI domains of Cas12a, facilitating the unwinding of the dsDNA target; 2) hybridization of the crRNA with the DNA target, triggering conformational changes in the REC lobe of Cas12a, that activates the RuvC domain; 3) *cis*-cleavage of the DNA target and release of the PAM-distal dsDNA; 4) *trans*-cleavage of non-specific ssDNA by the RuvC domain.


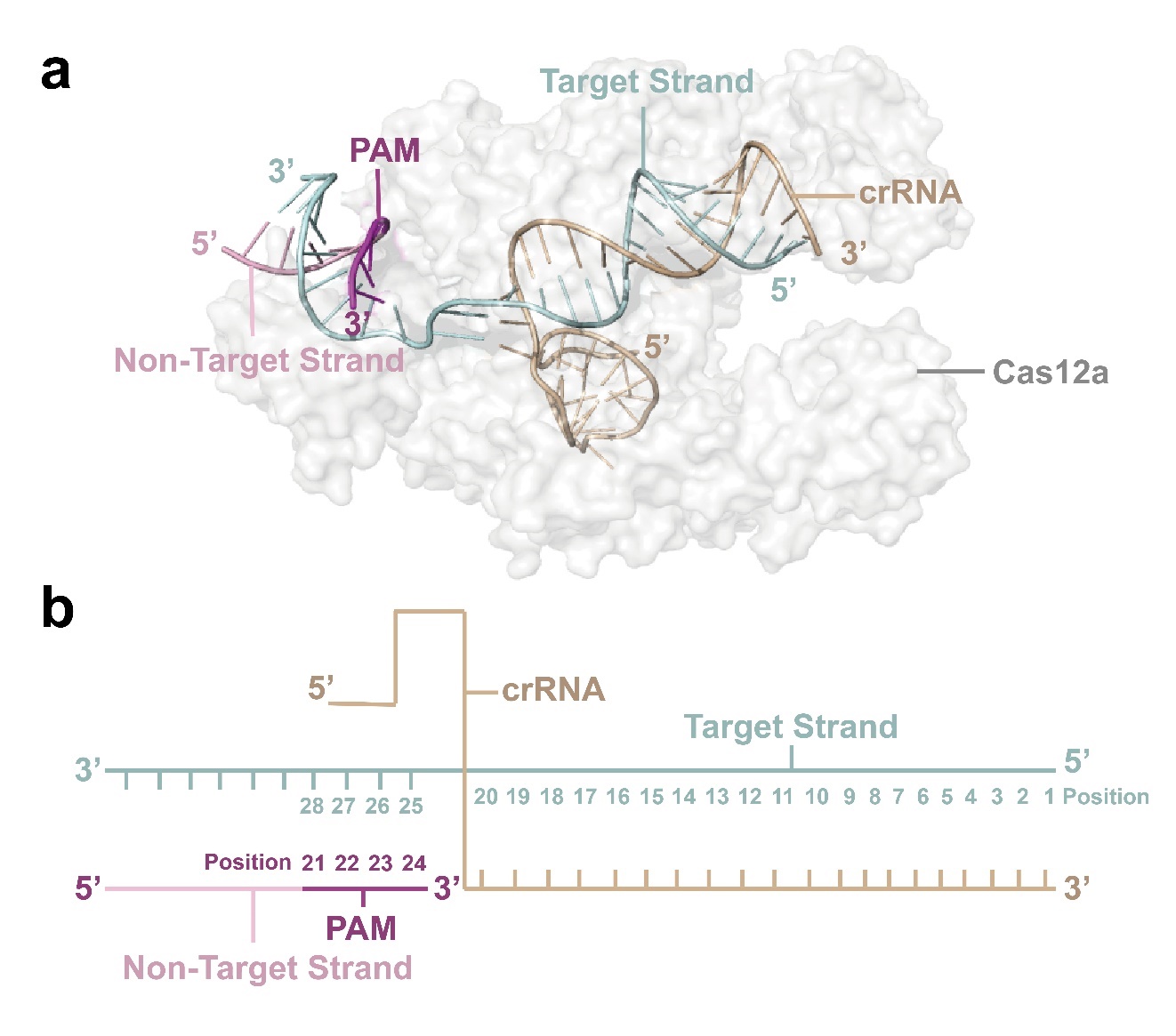


**Figure S2. Details of Cas12a/crRNA-DNA interactions.** (**a**) 3D structure diagram of Cas12a/crRNA-DNA (PDB ID: 5XUS)^[2]^. (**b**) Relative location map of crRNA-DNA. The analyzed sequences included the target strand located on position 1 to 20 (TS, 5’ - 3’), the PAM sequence located on position 21 to 24 (NTS, 5’ - 3’), and the PAM complementary sequence located on position 25 to 28 (TS, 5’ - 3’).


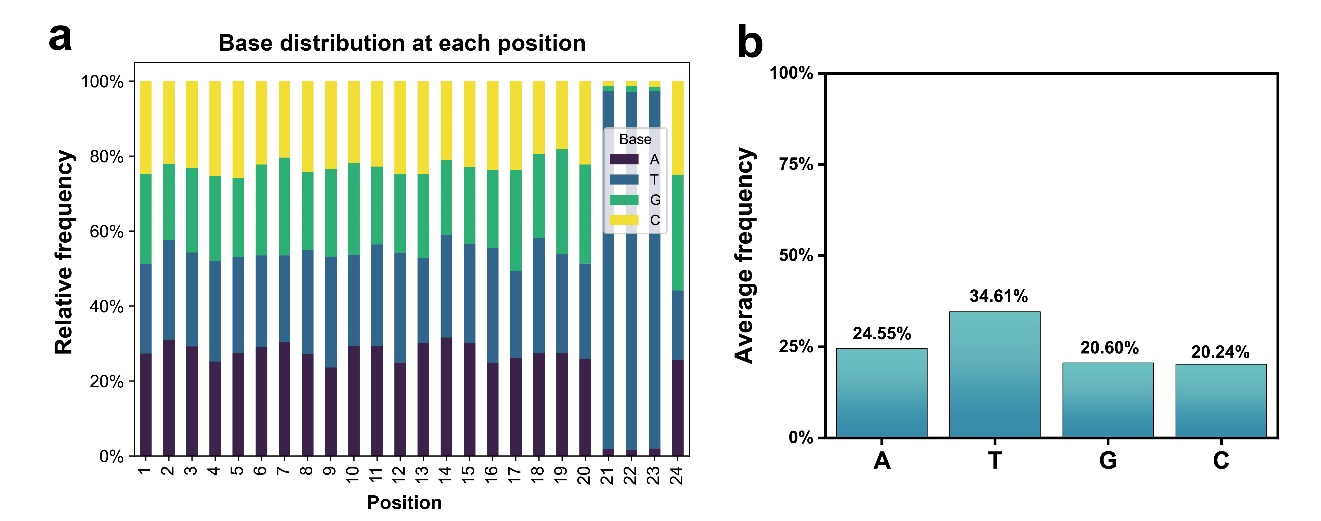


**Figure S3.** **Distribution of the prepared DNA target dataset**. (**a**) Relative frequency of base distribution at each position from position 1 to position 24. (**b**) Average frequency of four kinds of bases across all DNA targets.


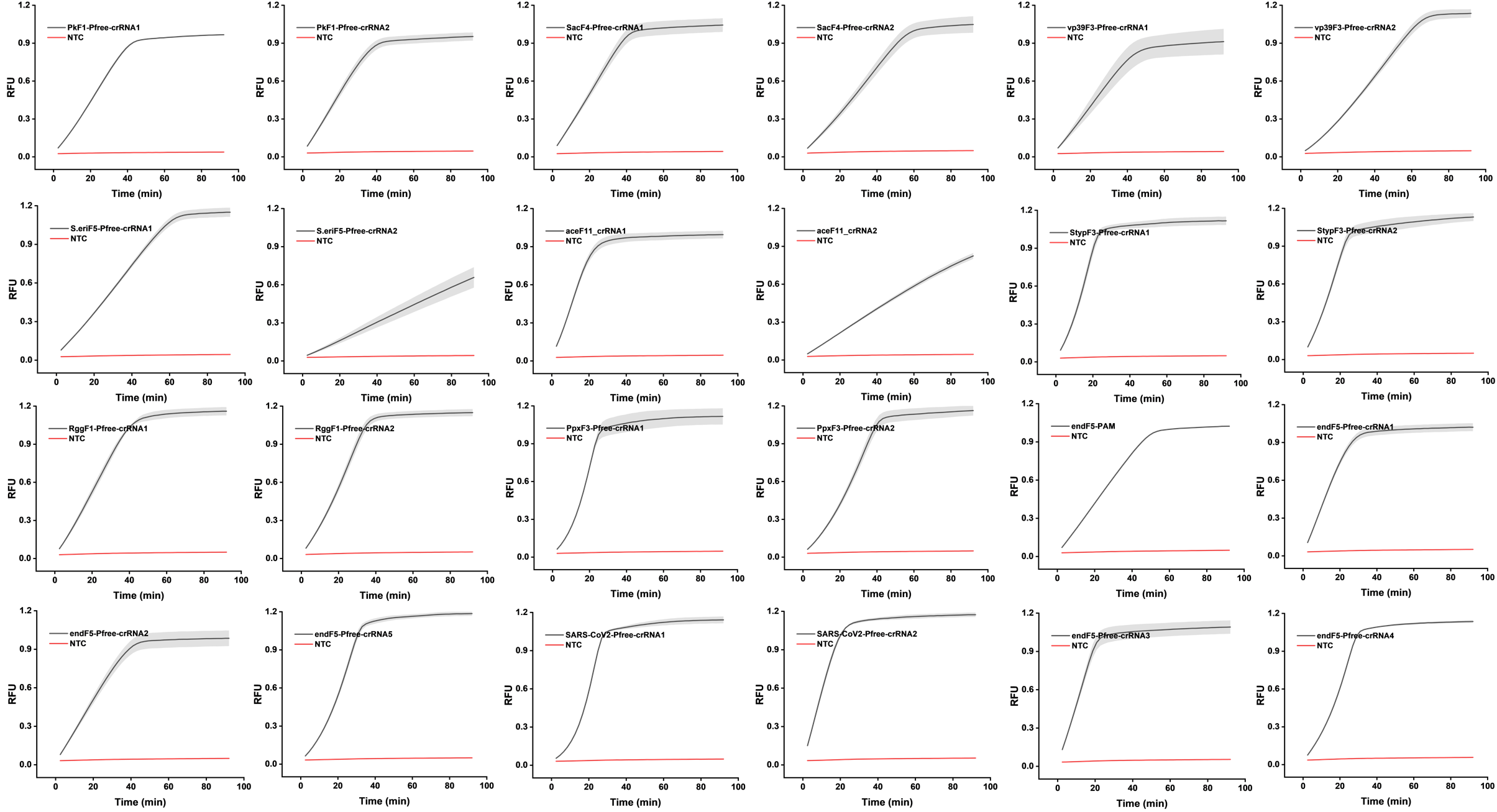


**Figure S4. Real-time fluorescence kinetics curves of 60 targets (Group 1).** The reaction was determined in 100 nM LbCas12a, 100 nM crRNA, 400 nM reporter and 3.5 nM dsDNA targets. The 60 targets included 20 PAM-included (TTTN, N = A/T/C/G) crRNAs and 40 PAM-free crRNAs. All data were presented as mean ± SD (n = 3).


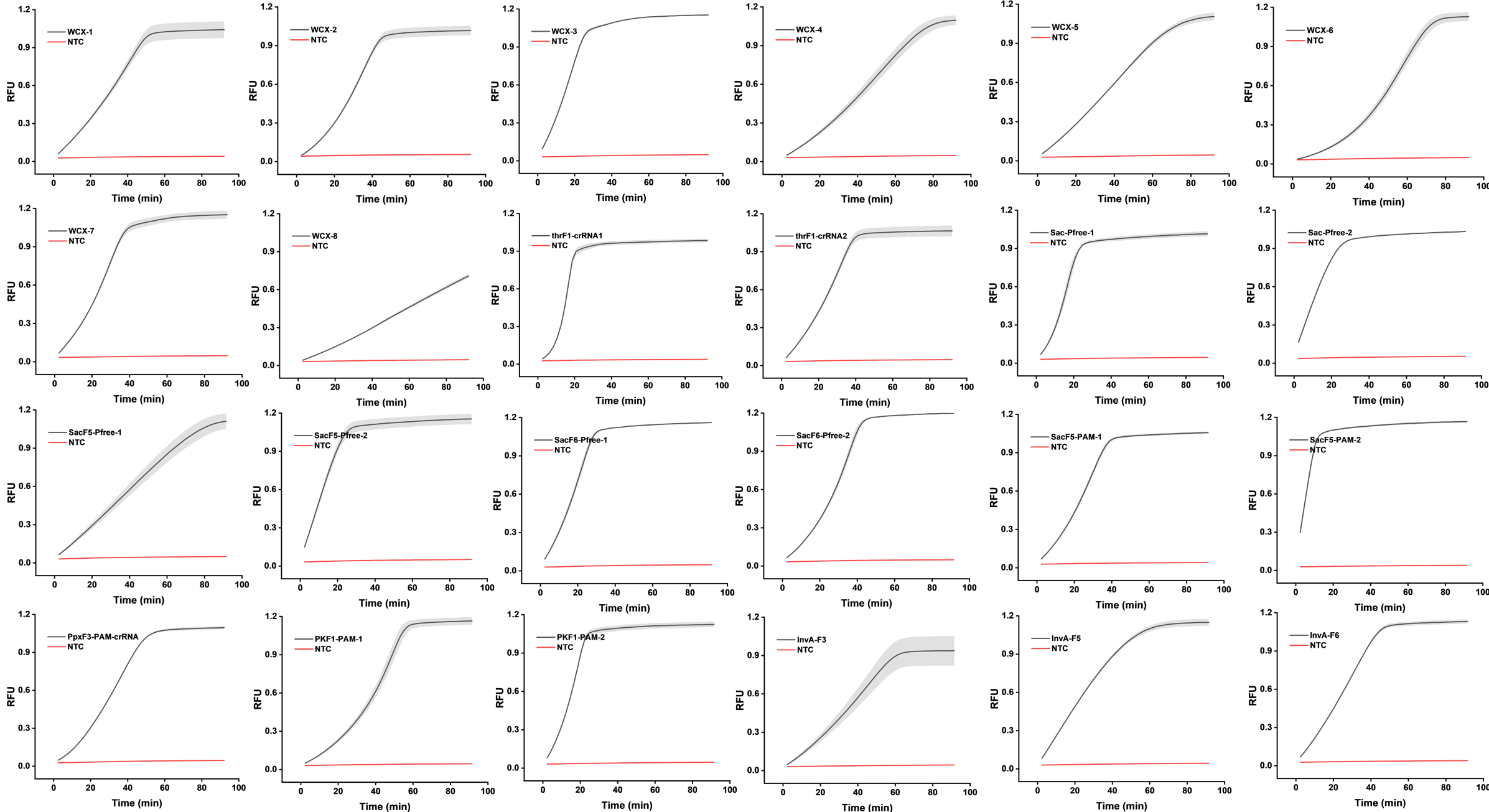


**Figure S4. Real-time fluorescence kinetics curves of 60 targets (Group 1).** The reaction was determined in 100 nM LbCas12a, 100 nM crRNA, 400 nM reporter and 3.5 nM dsDNA targets. The 60 targets included 20 PAM-included (TTTN, N = A/T/C/G) crRNAs and 40 PAM-free crRNAs. **(Continued)**


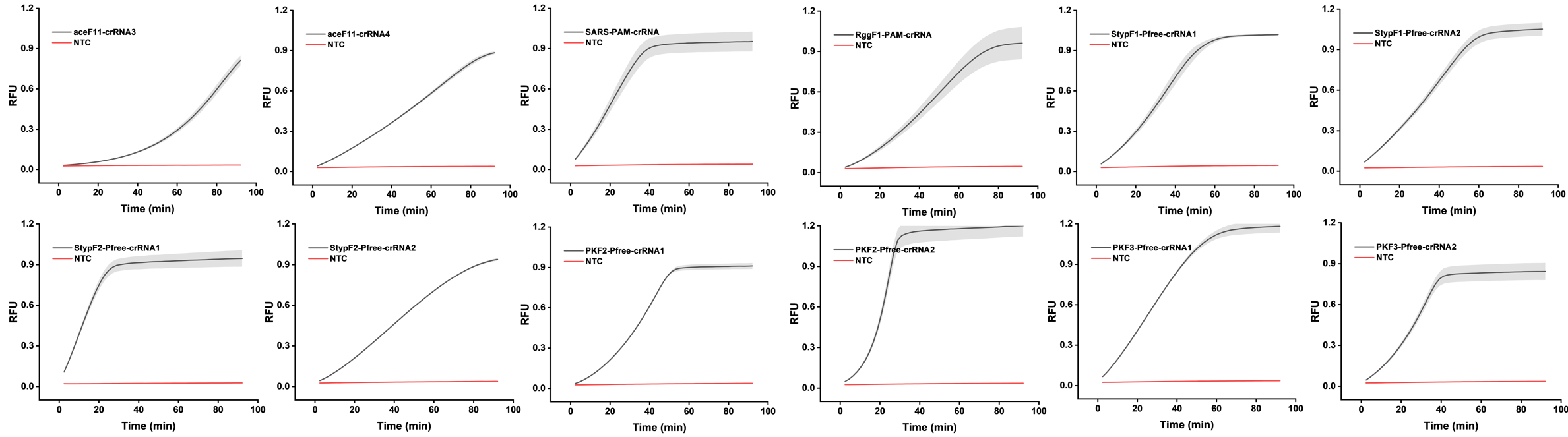


**Figure S4. Real-time fluorescence kinetics curves of 60 targets (Group 1).** The reaction was determined in 100 nM LbCas12a, 100 nM crRNA, 400 nM reporter and 3.5 nM dsDNA targets. The 60 targets included 20 PAM-included (TTTN, N = A/T/C/G) crRNAs and 40 PAM-free crRNAs. **(Continued)**


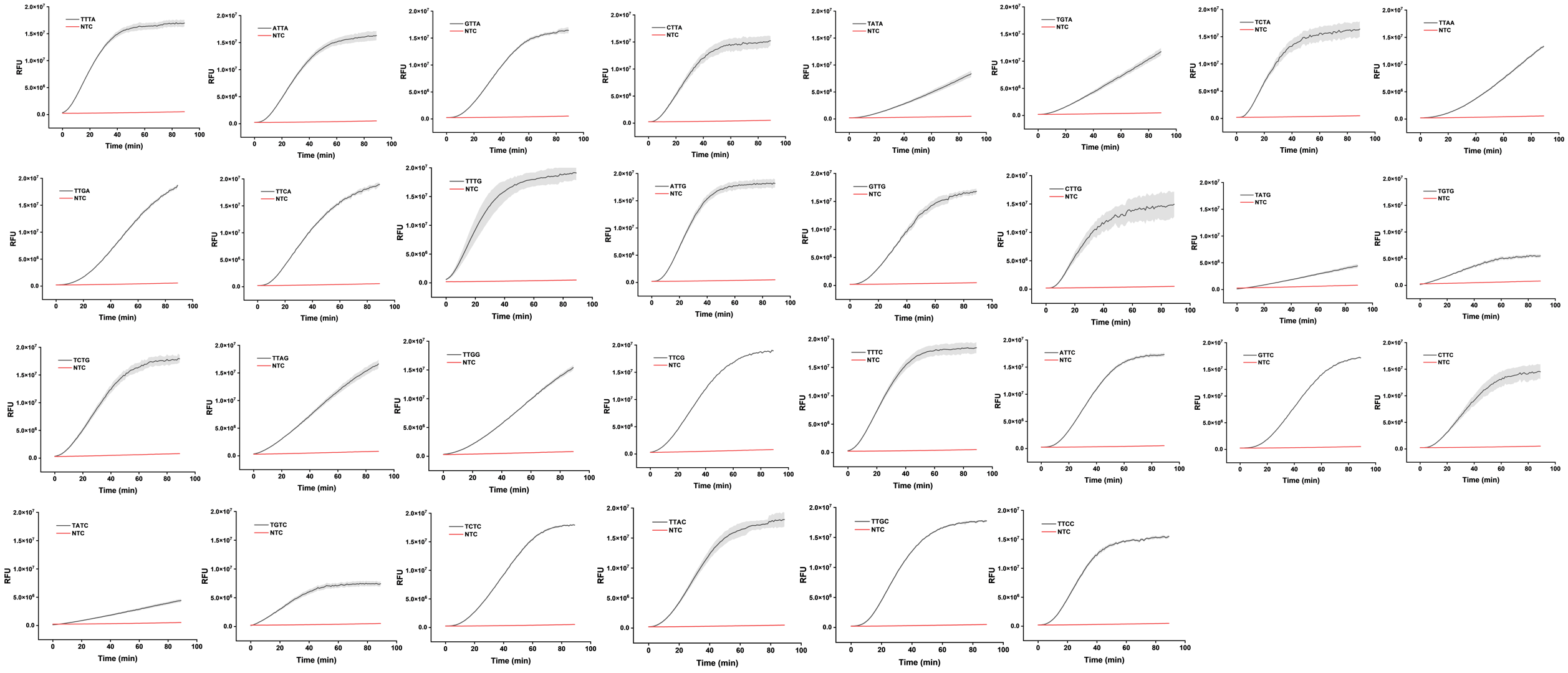


**Figure S5. Real-time fluorescence kinetics curves of 30 targets (Group 2) from Orf1ab** **gene spacers 4**. The targets were point-mutated from the TTTV (V = A/C/G) to VTTV, TVTV or TTVV. The fluorescence curves were drawn using raw data in the previous study^[3]^, that was determined in the reaction condition in 33 nM LbCas12a, 33 nM crRNA, 400 nM reporter and 3.5 nM dsDNA targets. All data were presented as mean ± SD (n = 3).


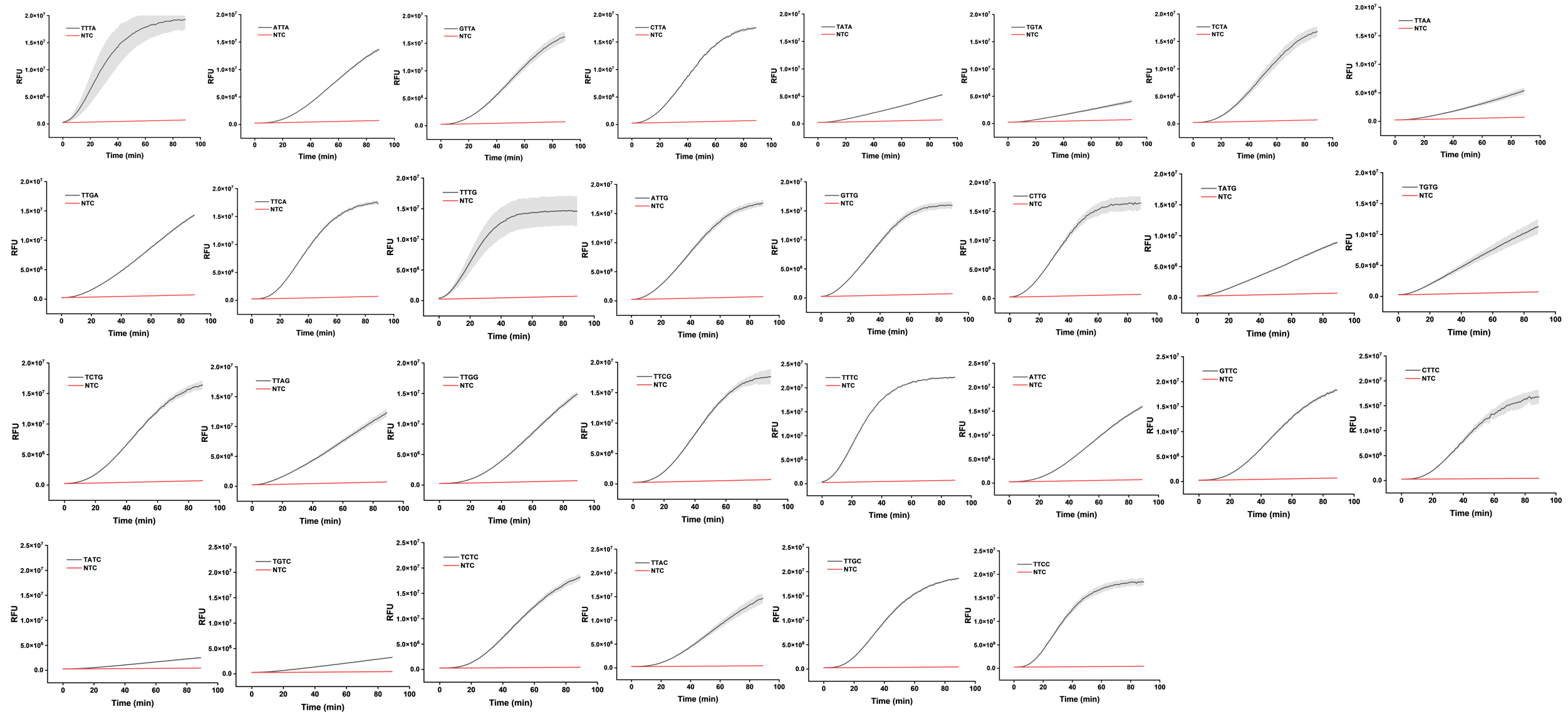


**Figure S6. Real-time fluorescence kinetics curves of 30 targets (Group 2) from Orf1ab** **gene spacers 5**. The targets were point-mutated from the TTTV (V = A/C/G) to VTTV, TVTV or TTVV. The fluorescence curves were drawn using raw data in the previous study^[3]^, that was determined in the reaction condition in 33 nM LbCas12a, 33 nM crRNA, 400 nM reporter and 3.5 nM dsDNA targets. All data were presented as mean ± SD (n = 3).


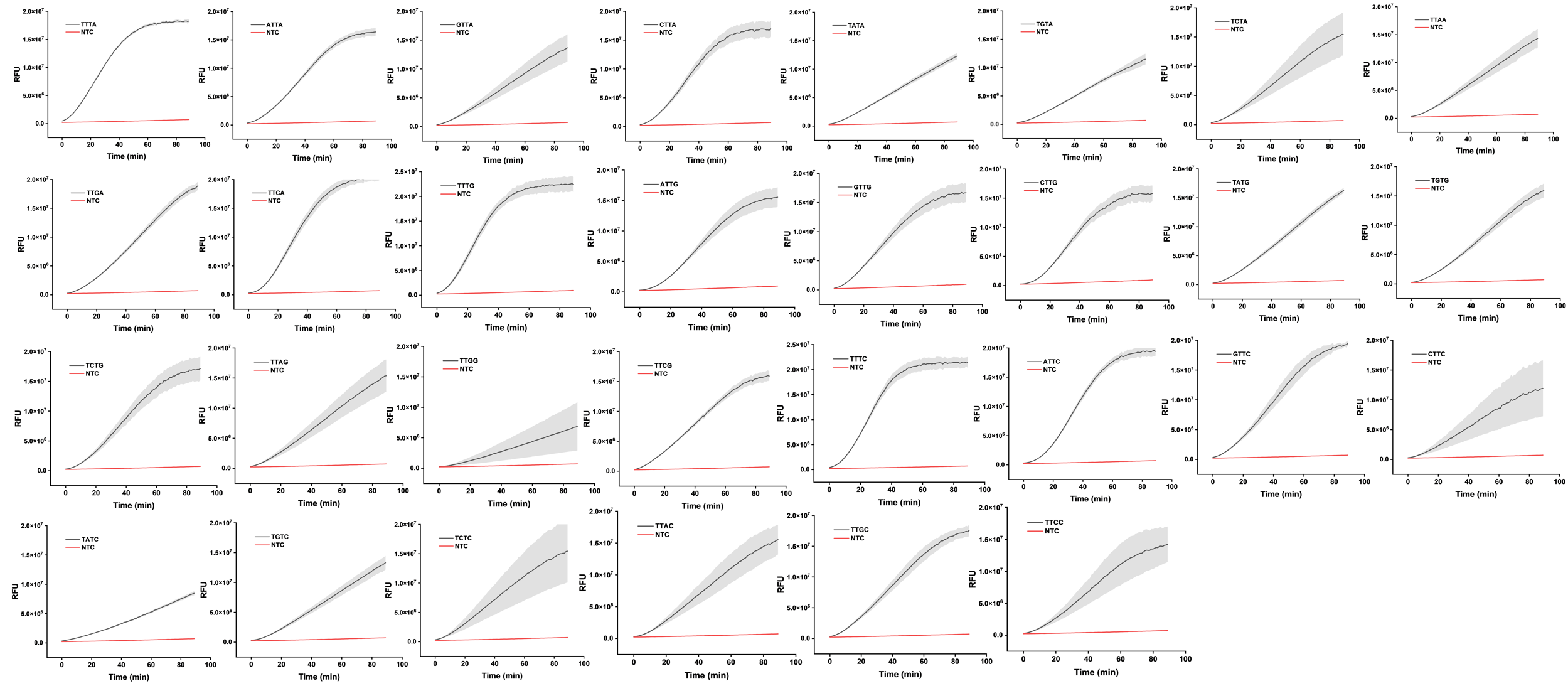


**Figure S7. Real-time fluorescence kinetics curves of 30 targets (Group 2) from S gene spacer 2**. The targets were point-mutated from the TTTV (V = A/C/G) to VTTV, TVTV or TTVV. The fluorescence curves were drawn using raw data in the previous study^[3]^, that was determined in the reaction condition in 33 nM LbCas12a, 33 nM crRNA, 400 nM reporter and 3.5 nM dsDNA targets. All data were presented as mean ± SD (n = 3).


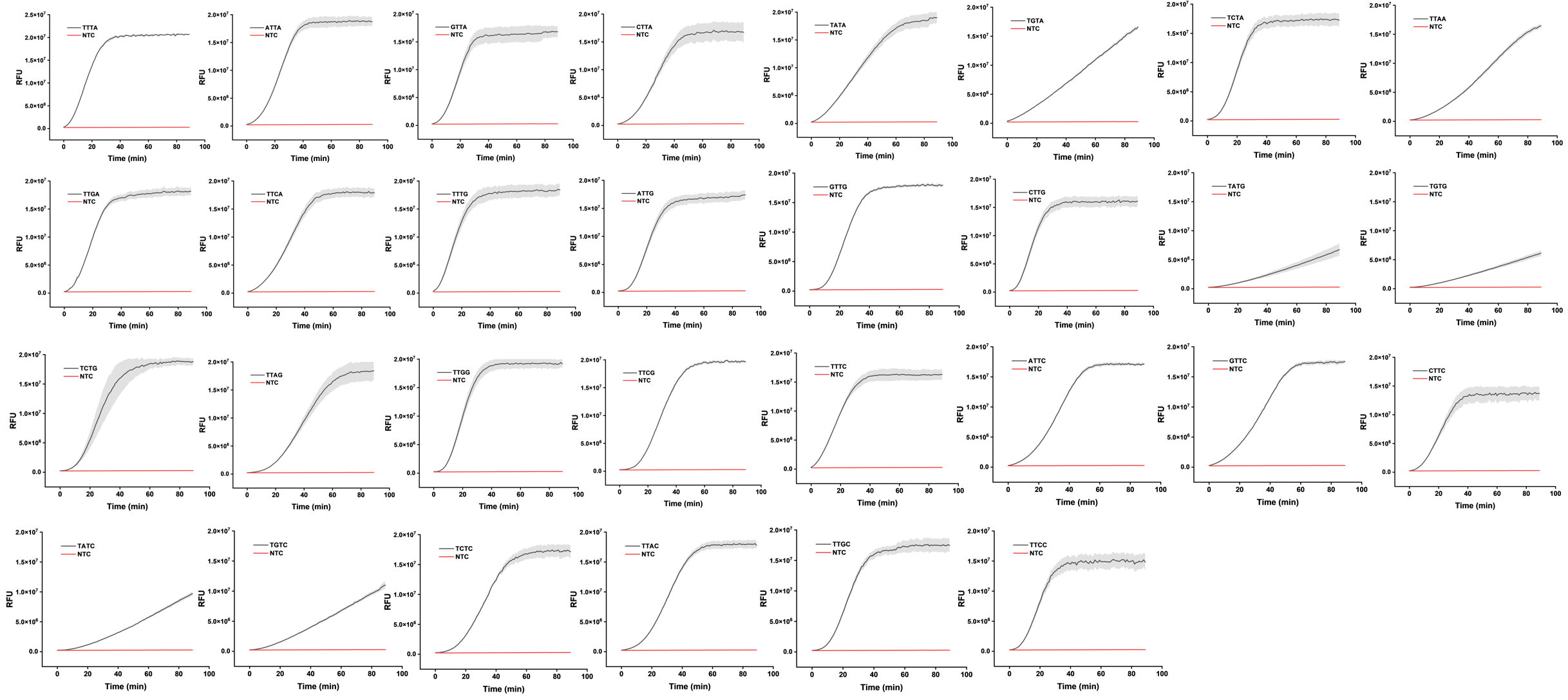


**Figure S8. Real-time fluorescence kinetics curves of 30 targets (Group 2) from HPV18 L1 gene spacer 1**. The targets were point-mutated from the TTTV (V = A/C/G) to VTTV, TVTV or TTVV. The fluorescence curves were drawn using raw data in the previous study^[3]^, that was determined in the reaction condition in 33 nM LbCas12a, 33 nM crRNA, 400 nM reporter and 3.5 nM dsDNA targets. All data were presented as mean ± SD (n = 3).


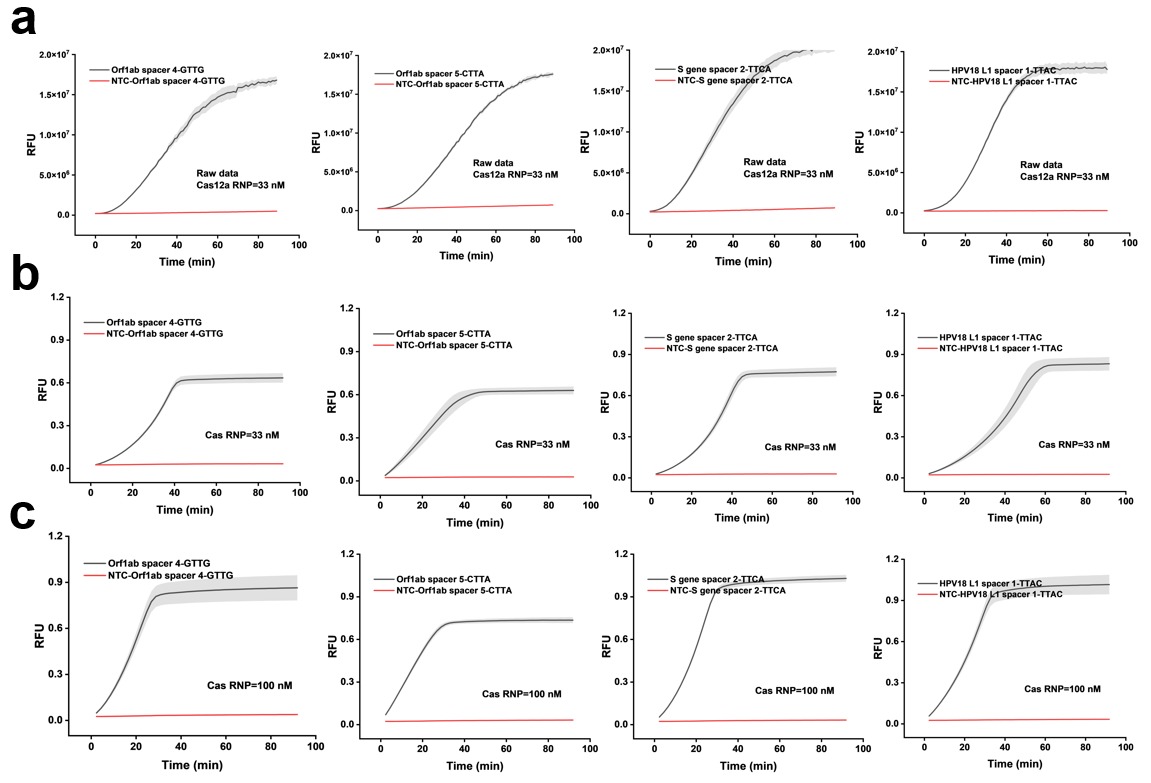


**Figure S9. Real-time fluorescence kinetics curves of 4 targets (Group 2) including Orf1ab spacer 4-GTTG, Orf1ab spacer 5-CTTA, S gene spacer 2- TTCA, and HPV18 L1 gene spacer 1-TTAC**. **(a)** The fluorescence curves were drawn using raw data in the previous study^[3]^, that was determined in the reaction condition in 33 nM LbCas12a, 33 nM crRNA, 400 nM reporter and 3.5 nM dsDNA targets. **(b)** The fluorescence curves were determined in the reaction condition in 33 nM LbCas12a, 33 nM crRNA, 400 nM reporter and 3.5 nM dsDNA targets in this study. **(c)** The fluorescence curves were determined in the reaction condition in 100 nM LbCas12a, 100 nM crRNA, 400 nM reporter and 3.5 nM dsDNA targets in this study. All data were presented as mean ± SD (n = 3).


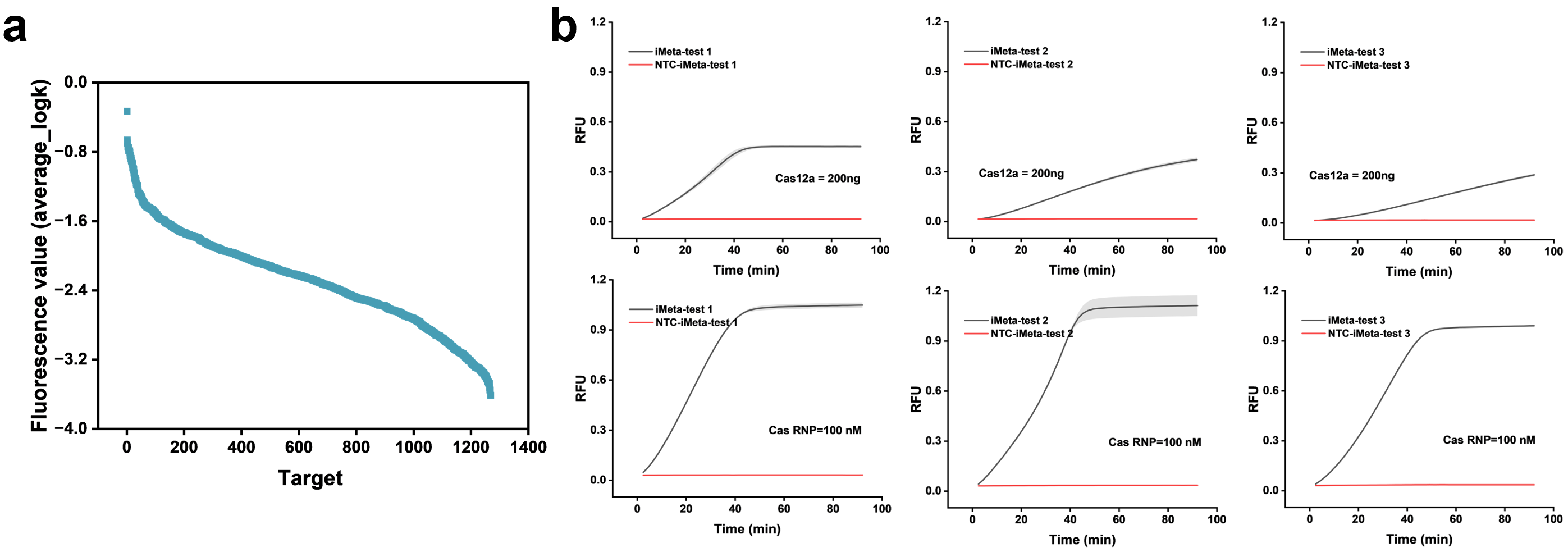


**Figure S10. The fluorescence value of 1,268 targets and real-time fluorescence kinetics curves of 3 test targets from Group 3**. **(a)** The curve was drawn using raw data in the previous study^[4]^, that was normalized fluorescence value determined at 30 min based on 20 min recorded data of 1,268 targets. **(b)** The fluorescence curves were determined in the two reaction conditions: 1) in 200 ng LbCas12a, 200 ng crRNA, 400 nM reporter and 3.5 nM dsDNA targets; 2) 100 nM LbCas12a, 100 nM crRNA, 400 nM reporter and 3.5 nM dsDNA targets in this study. All data were presented as mean ± SD (n = 3).


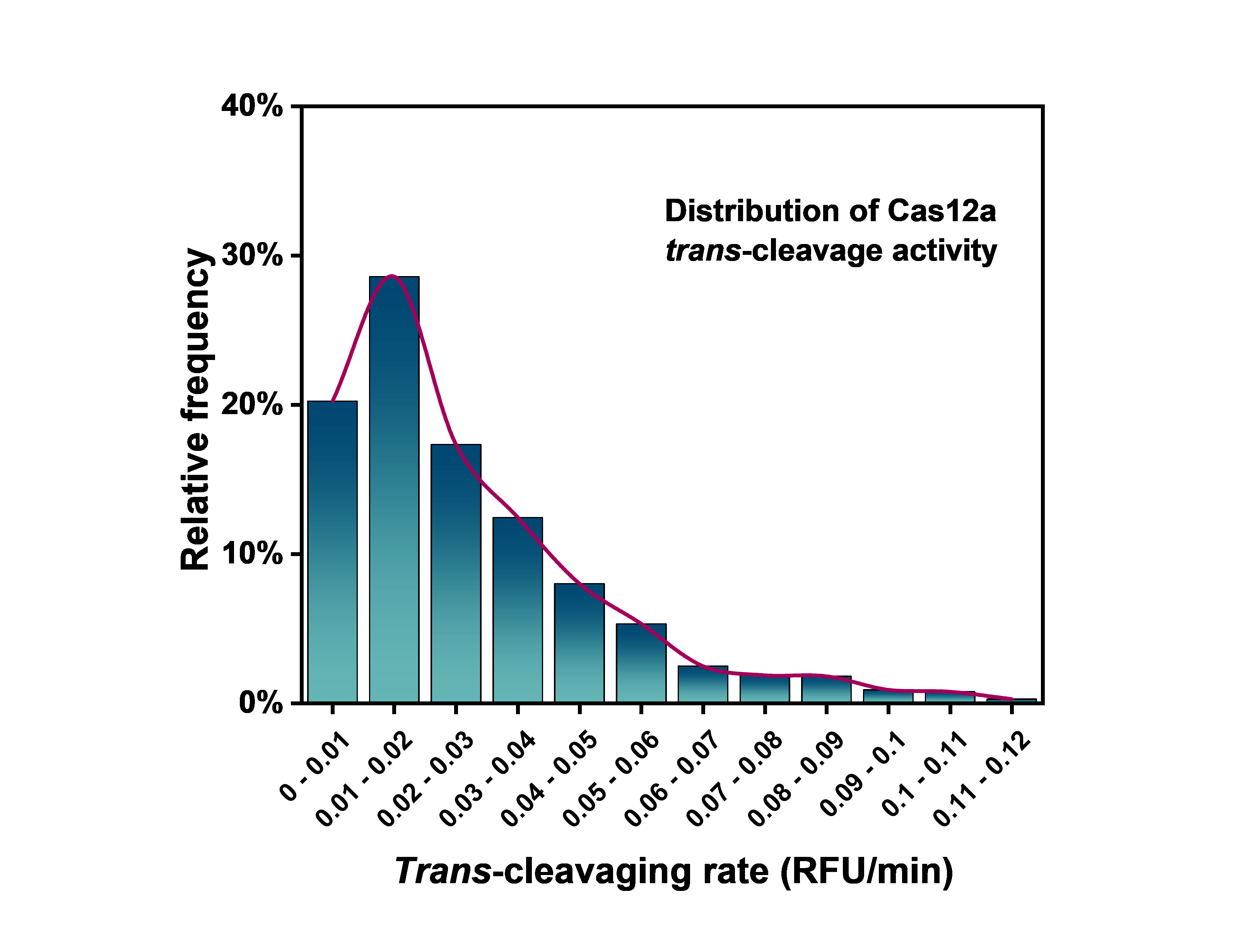


**Figure S11. Distribution of Cas12a *trans*-cleavage activity from the crRNA-DNA dataset including Group 1, Group 2, and Group 3 (1,448 targets in total)**. The *trans*-cleavage activity of 60 targets (Group 1) was determined in 100 nM LbCas12a, 100 nM crRNA, 400 nM reporter and 3.5 nM dsDNA targets. The *trans*-cleavage activity of 120 targets (Group 2) was acquired by normalizing the data of 4 targets (Orf1ab spacer 4-GTTG, Orf1ab spacer 5-CTTA, S gene spacer 2- TTCA, and HPV18 L1 gene spacer 1-TTAC) under the reaction condition of 33 nM and 100 nM LbCas12a/crRNA. The *trans*-cleavage activity of 1,268 targets (Group 3) was acquired by normalizing the data of 3 targets (iMeta-test 1, iMeta-test 2, and iMeta-test 3) under the reaction condition of 200 ng and 100 nM LbCas12a/crRNA.


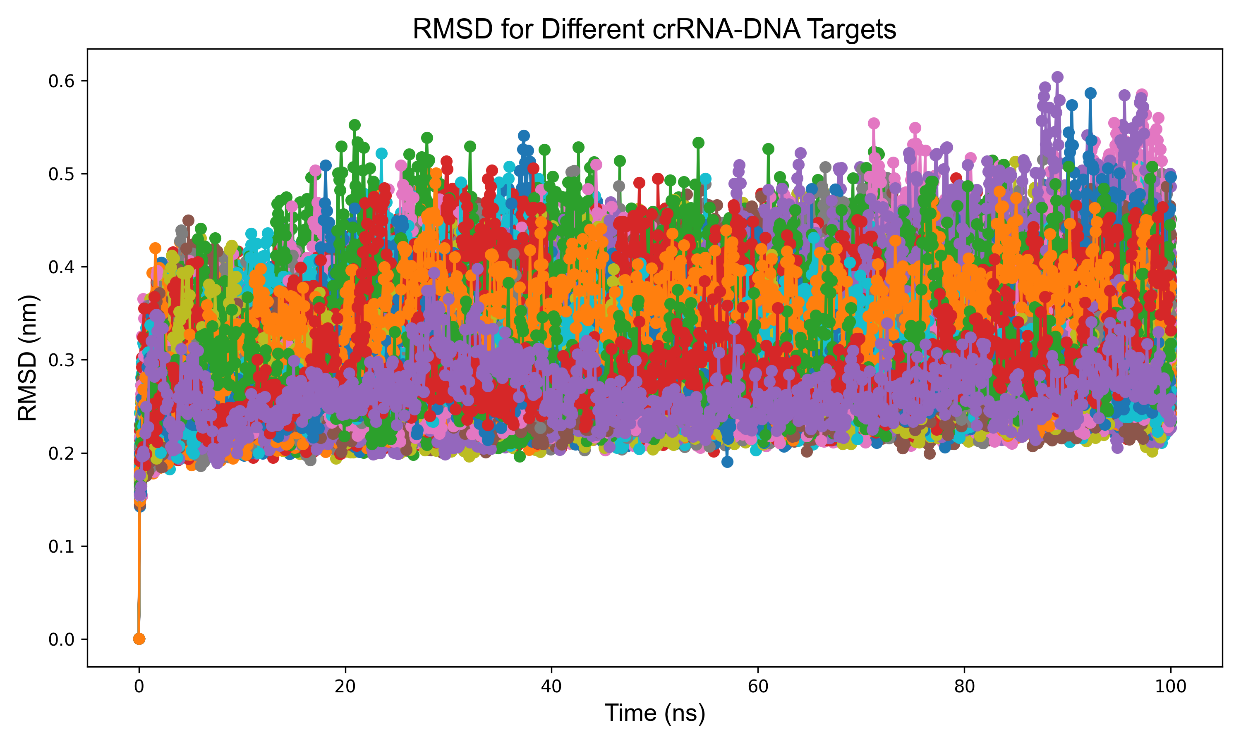


**Figure S12. RMSD values of 180 Cas12a/crRNA-DNA complexes throughout the 100 ns MD simulations.** Different colors represent different crRNA-DNA targets.


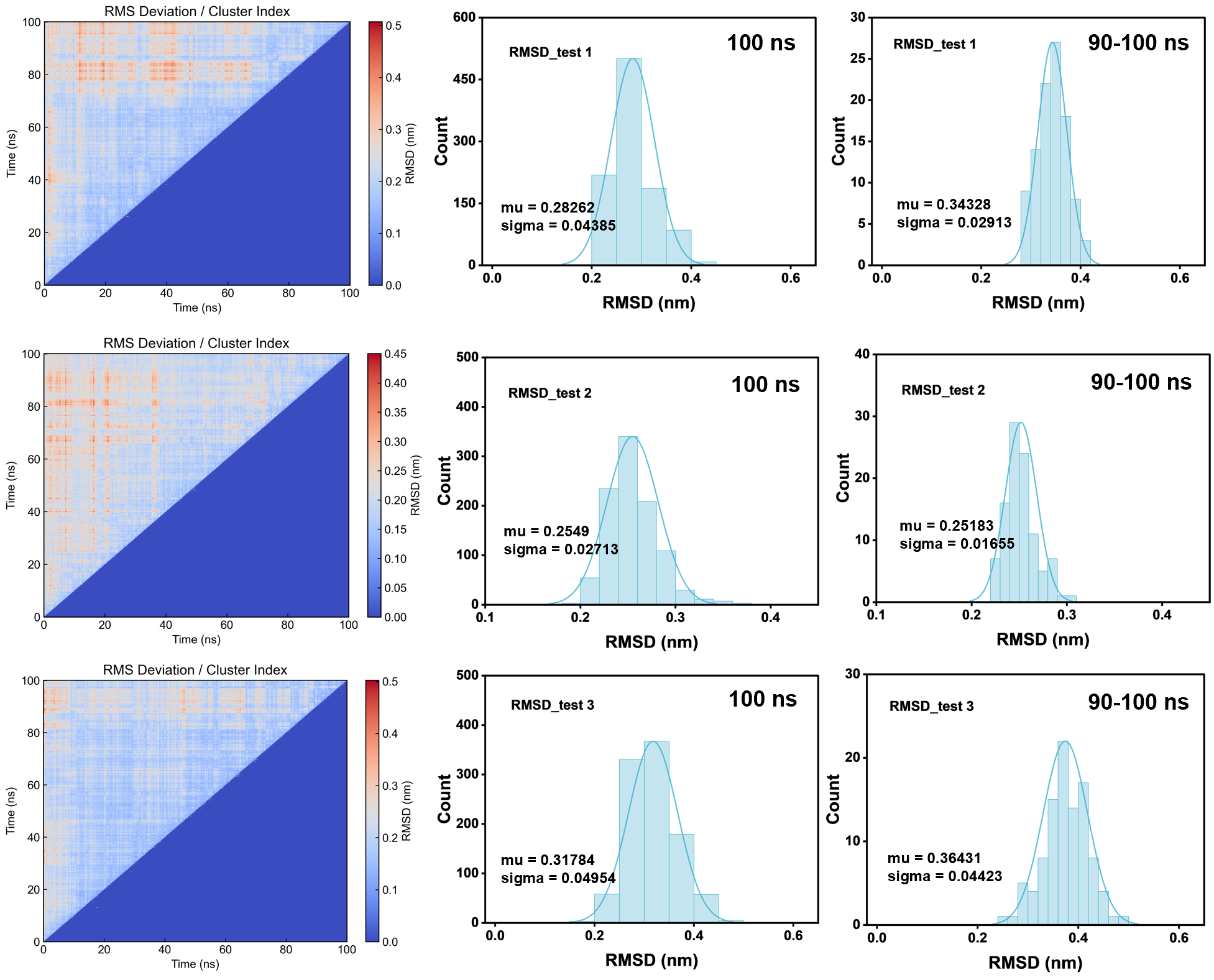


**Figure S13. RMSD distributions for three test targets with high, medium, and low Cas12a *trans*-cleavage activities in the 100 ns and 90-100 ns intervals.** mu: mean value, sigma: standard deviation.


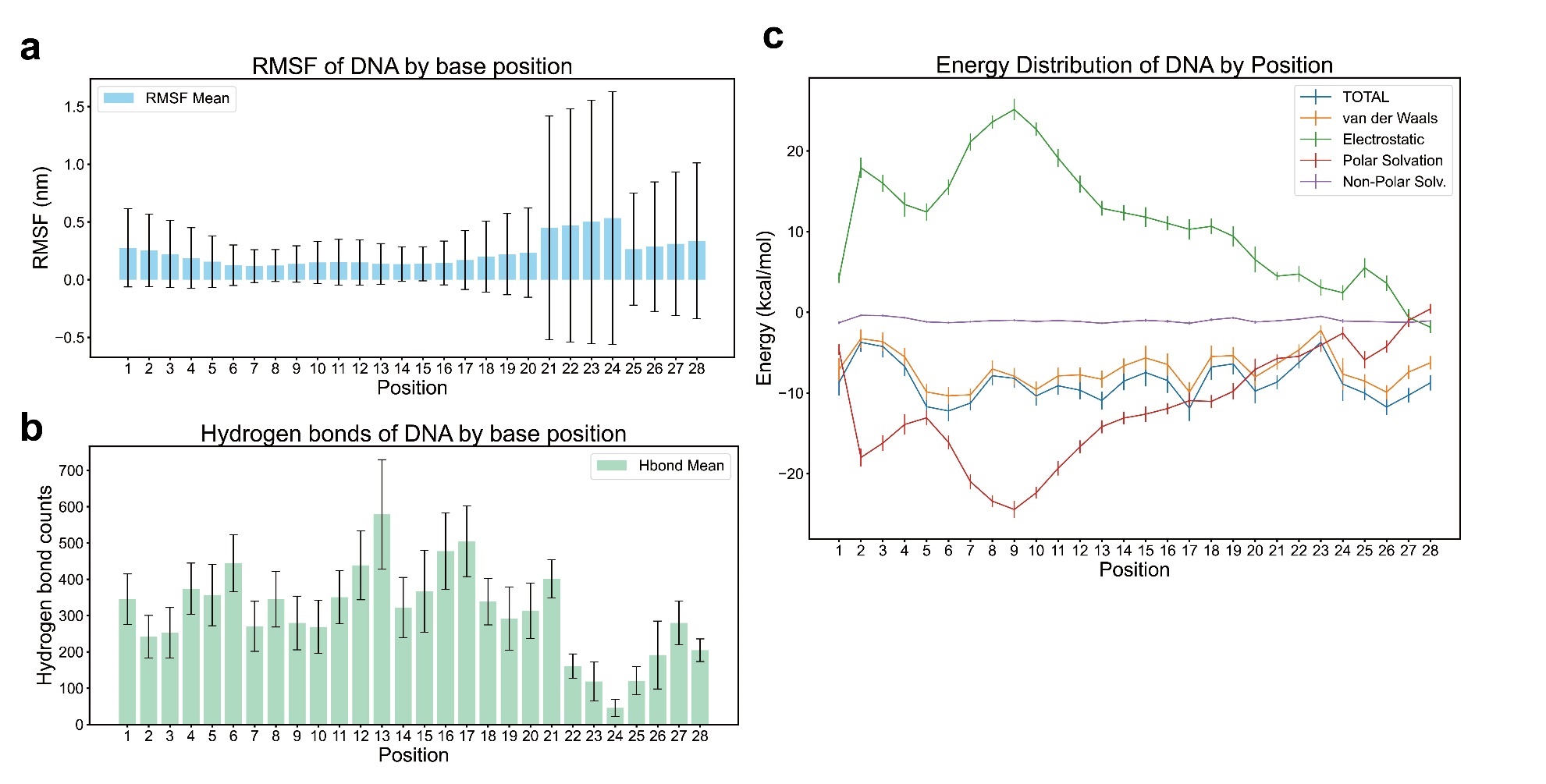


**Figure S14. Molecular interaction features of DNA in the CRISPR-Cas12a system across the base position.** The average RMSF distribution (**a**), hydrogen bond count distribution (**b**), and energy distribution (**c**) by the base position from 1 to 24. All data were presented as mean ± SD (n = 3).

**
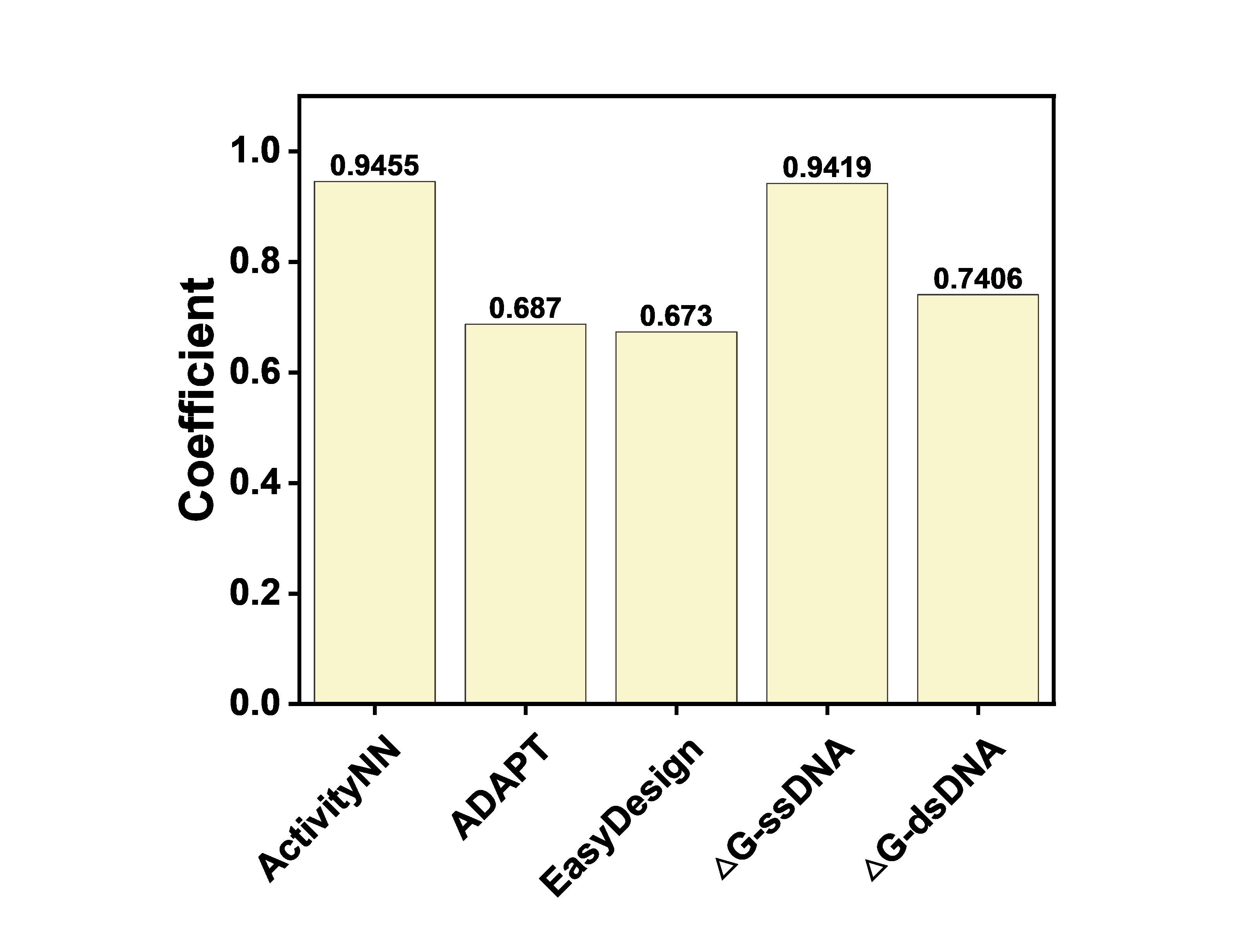
**

**Figure S15.** **Comparison of the deep learning model (ActivityNN) with existing methods reported in the literatures for predicting Cas12a *trans*-cleavage activity.** They included ADAPT^[5]^, EasyDesign^[4]^, △G-ssDNA^[6]^, and △G-dsDNA^[6]^.


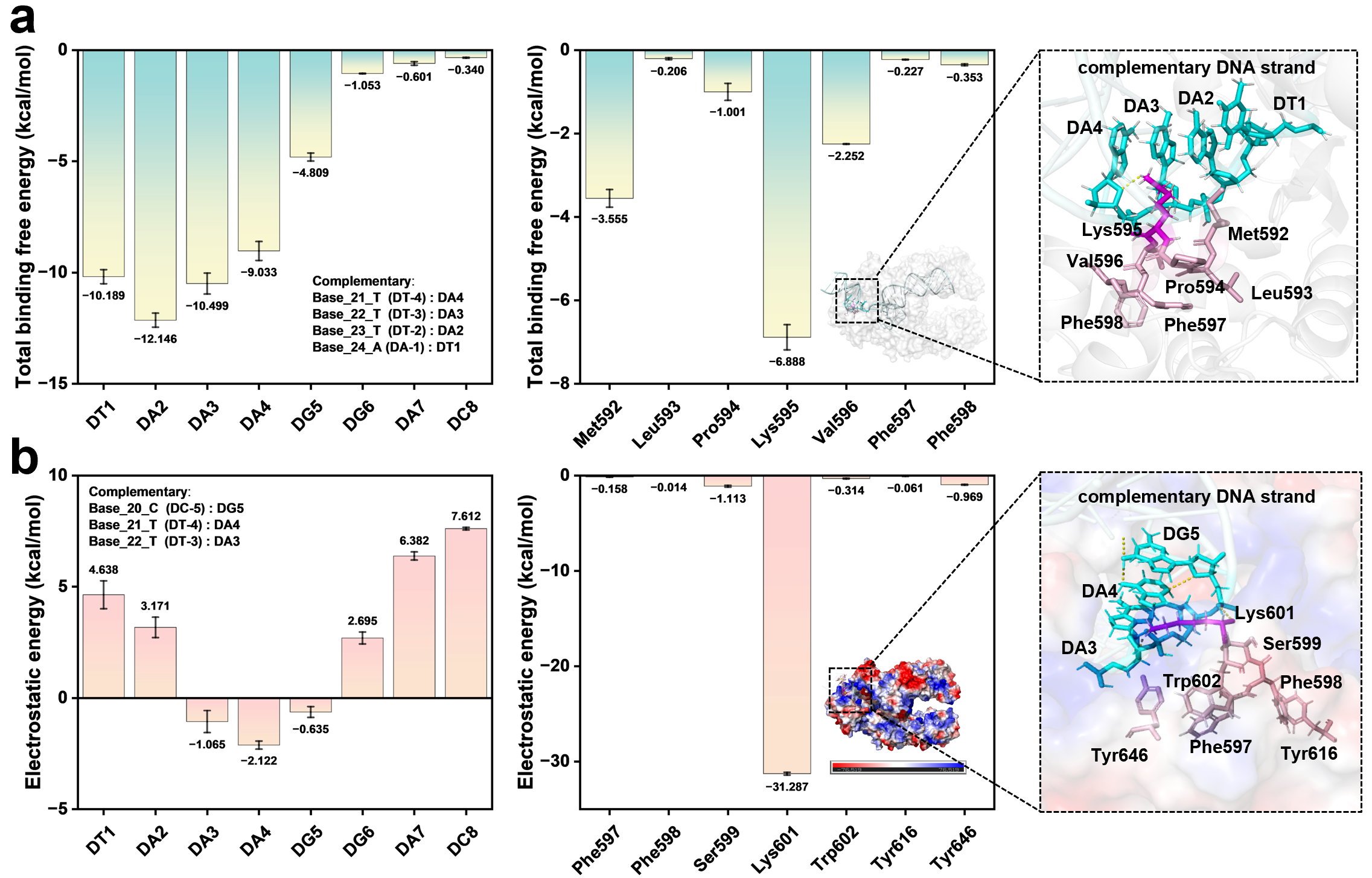


**Figure S16.** **Molecular interaction between complementary DNA bases and amino acids. (a)** Binding free energy calculations and interaction site diagram for complementary DNA bases and amino acids. The binding energies between complementary DNA bases (DA4, DA3, DA2, DT1) and the amino acids (e.g., Lys595, Met592) were shown. **(b)** Electrostatic energy calculations and interaction site diagram for complementary DNA bases and amino acids. The electrostatic interactions between complementary DNA bases (DG5, DA4, DA3) and the amino acids (e.g., Lys601) were depicted.


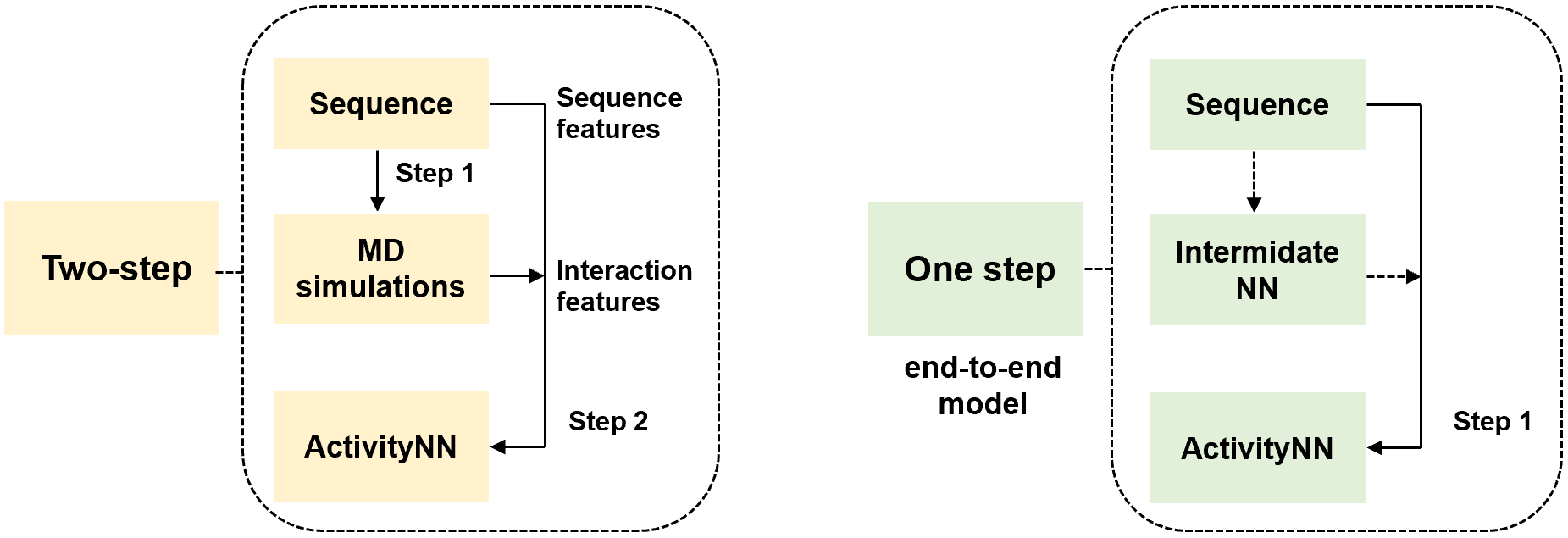


**Figure S17.** **Scheme of the Two-step model and One-step model.** The Two-step model was firstly conducted by performing MD simulations to acquire molecular interaction features, then combined with sequence features to input into the ActivityNN model for activity prediction. The One-step model was started from the input sequence features, and directly output the Cas12a *trans*-cleavage activity prediction value.

**
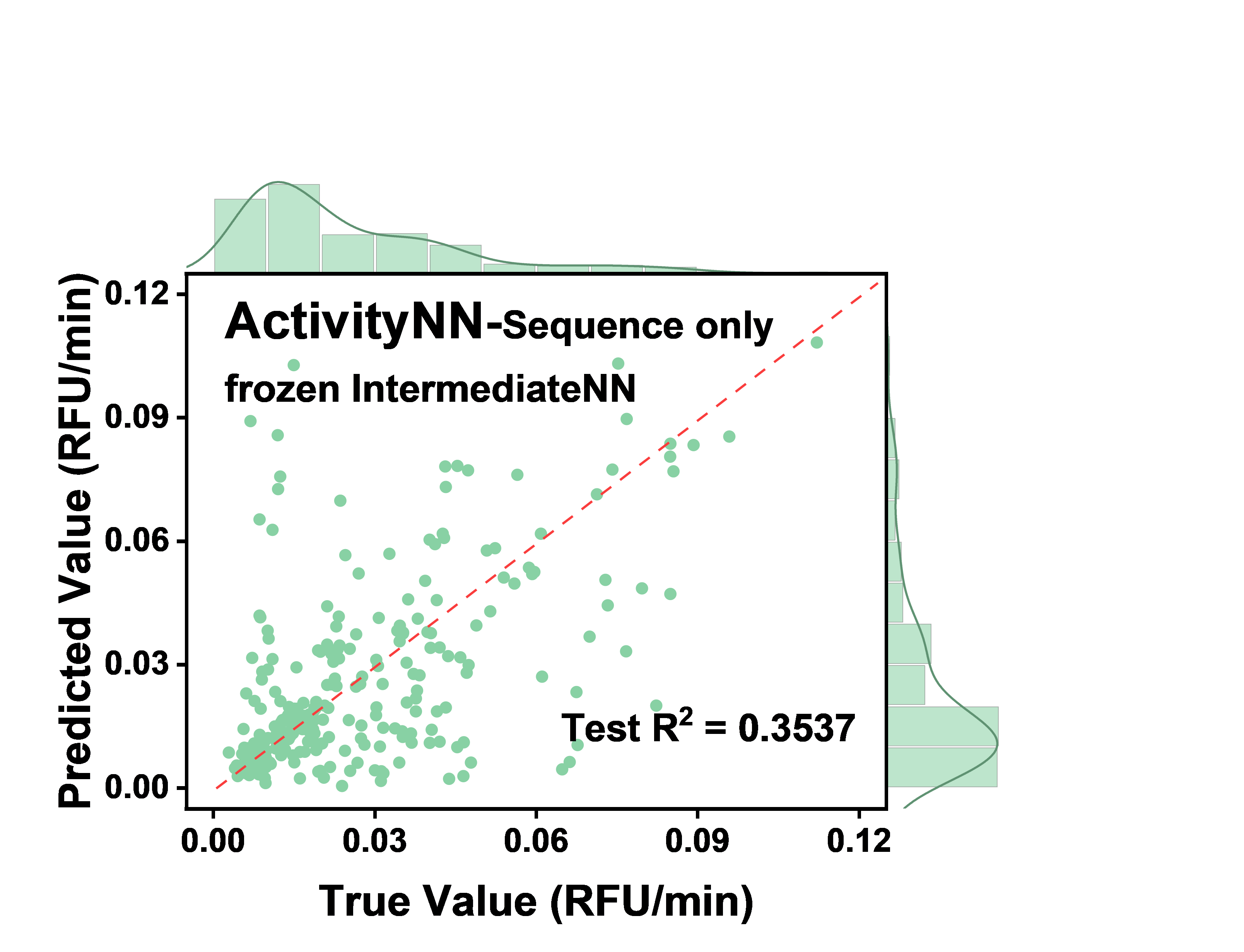
**

**Figure S18.** **Prediction performance using the deep learning model (ActivityNN) for predicting Cas12a *trans*-cleavage activity under sequence-only feature datasets.** RFU: relative fluorescence units.


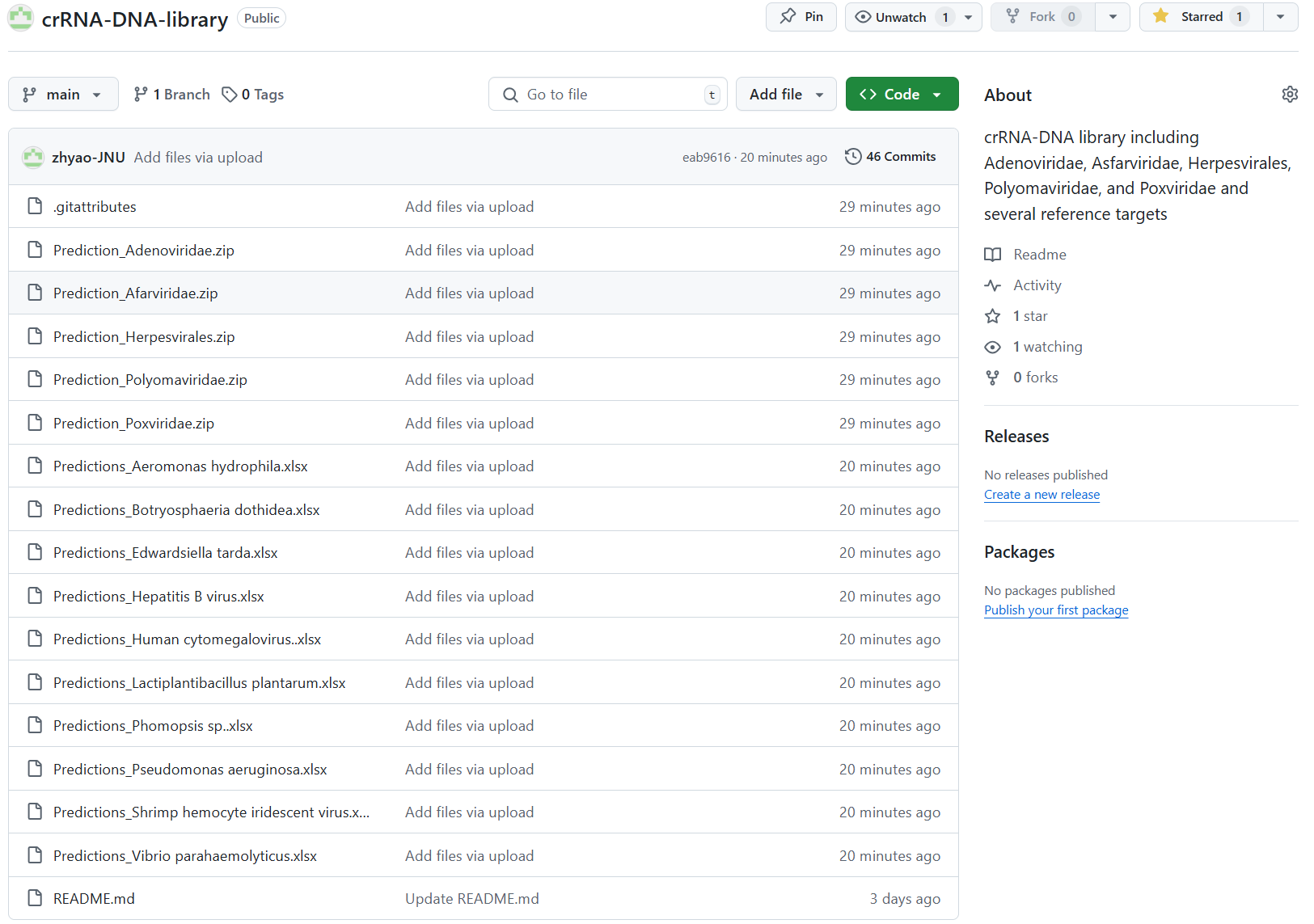


**Figure S19. The Web page of** **crRNA-DNA library including tremendous viral and bacterial targets and the corresponding predicted Cas12a *trans*-cleavage activity.** To facilitate convenient usage of crRNA-DNA library, we have uploaded it to a user-friendly web (<https://github.com/zhyao-JNU/crRNA-DNA-library>).

**
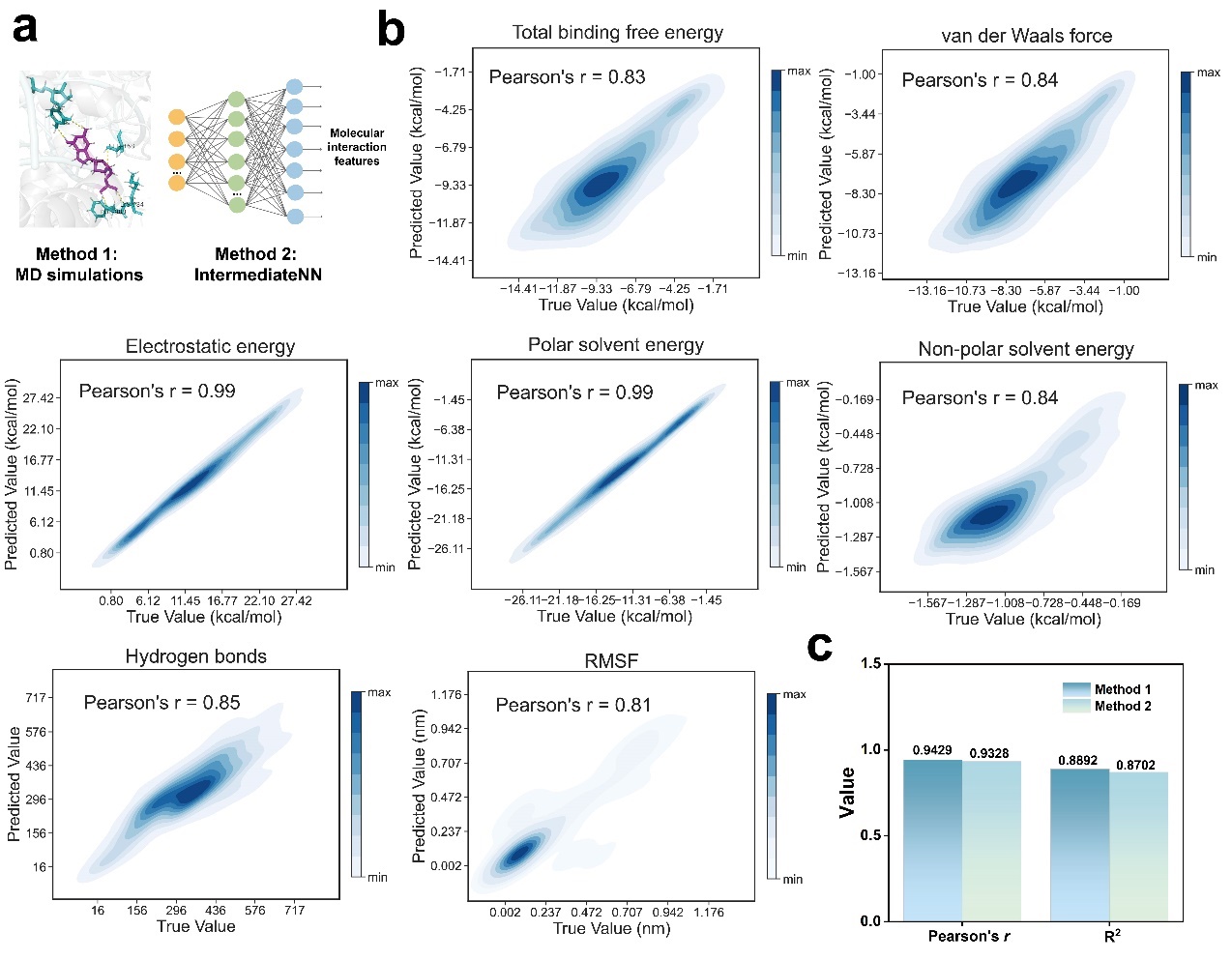
**

**Figure S20.** **Comparison of two methods for predicting seven molecular interaction features and activity. (a)** Method 1 was performed by long-time scale MD simulations, while method 2 was performed by the IntermediateNN model. **(b)** The Kernel density estimation of true value (x-axis) and predicted values (y-axis) between two calculation methods of molecular interaction features of DNA (e.g., binding energy, hydrogen bonds, RMSF). (**c**) Comparison between the predicted and experimentally verified Cas12a *trans*-cleavage activity by using the two methods.

**
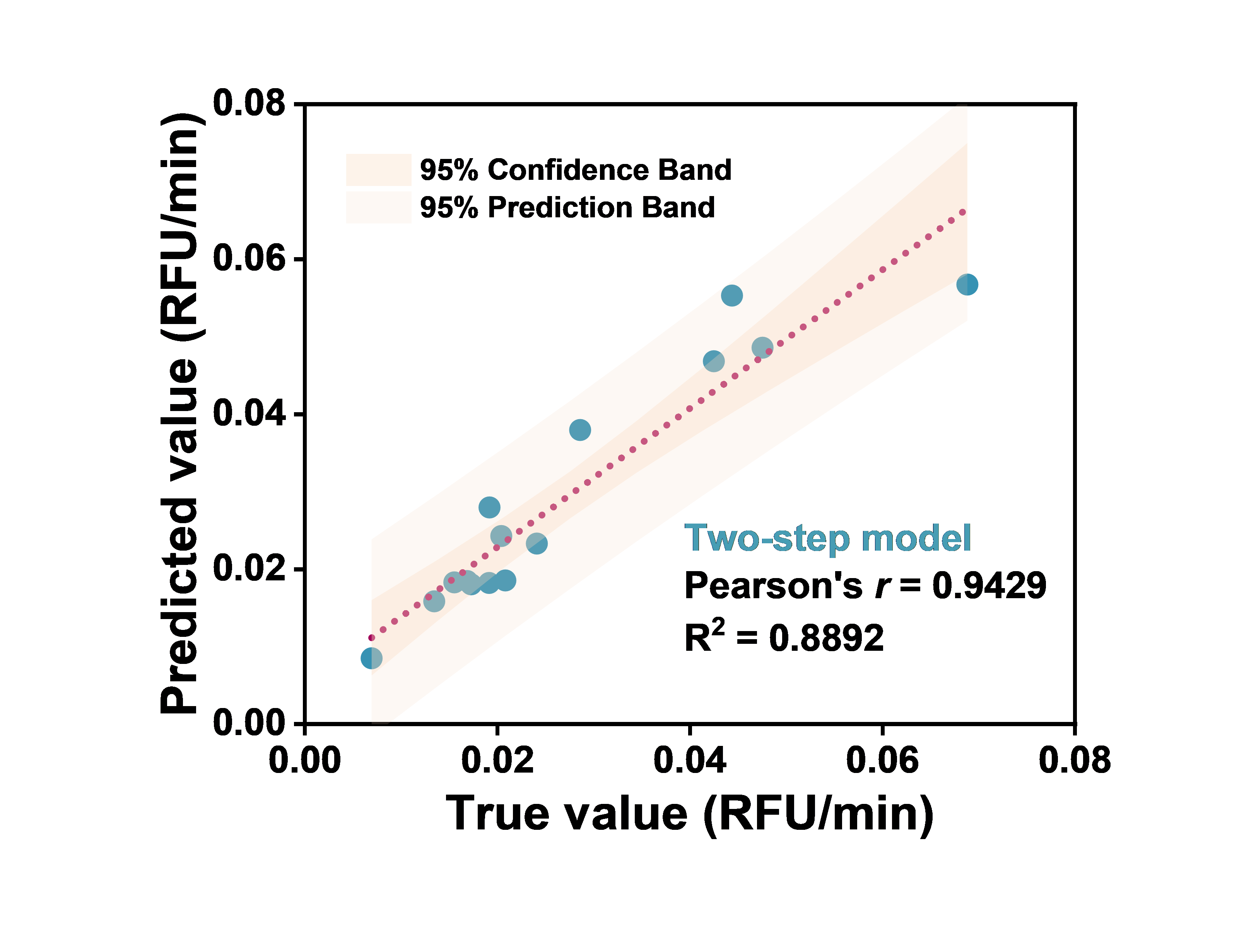
**

**Figure S21.** **Prediction performance of the Two-step model.** Liner fitting between the predicted and experimentally verified Cas12a *trans*-cleavage activity by using the Two-step model. RFU: relative fluorescence units.

**
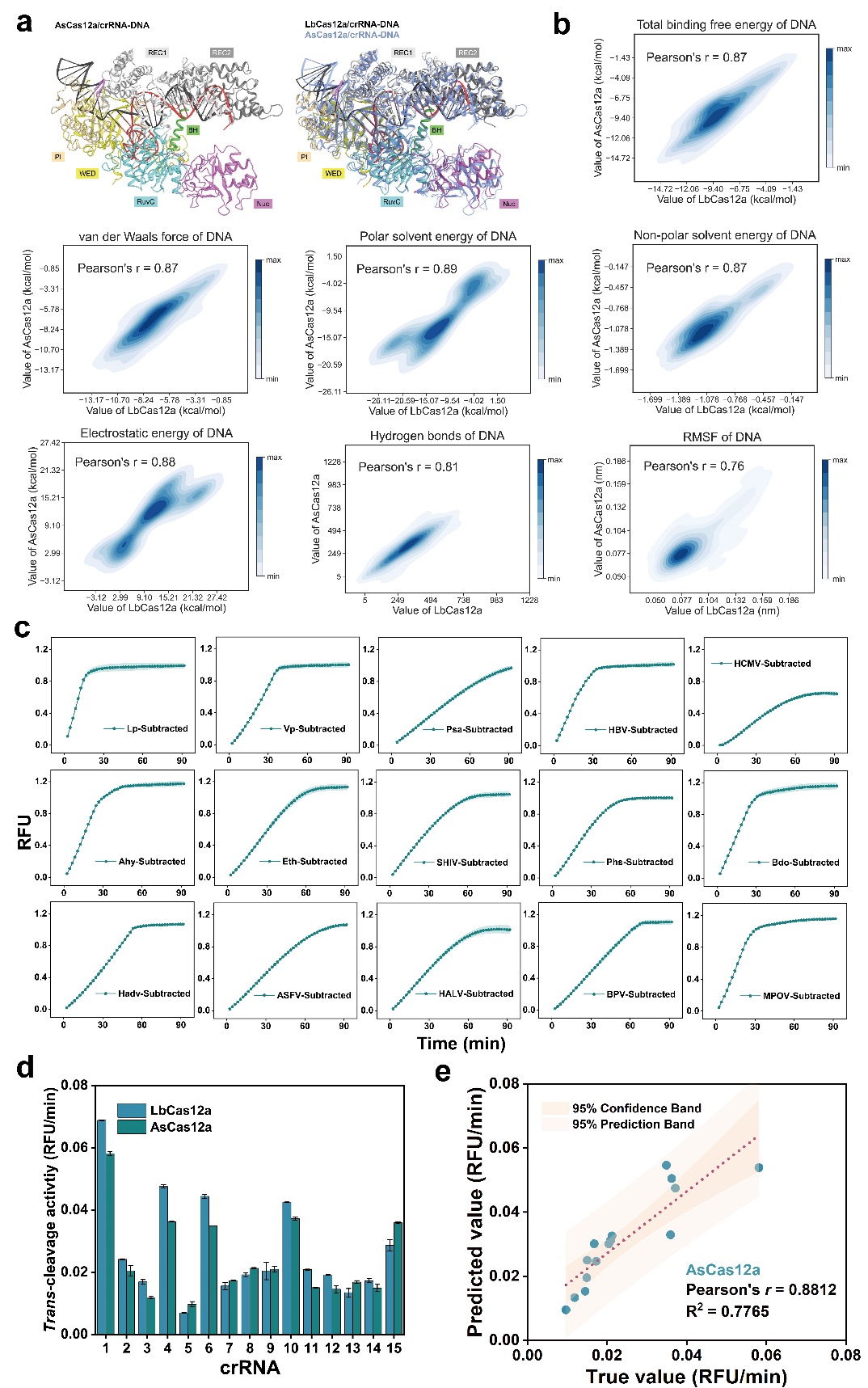
**

**Figure S22.** **Comparison of the interaction features and activity between AsCas12a and LbCas12a. (a)** Comparison of structures between AsCas12a (PDB ID: 5B43) and LbCas12a (PDB ID: 5XUS)^[2, 7]^. (**b**) Correlation analysis of molecular interaction features of DNA between LbCas12a and AsCas12a by the Kernel density map. (**c**) Experimental validation of 15 representative targets for AsCas12a by using the real-time fluorescence kinetics detection. (**d**) Comparison of the *trans*-cleavage activity distribution between AsCas12a and LbCas12a. (**e**) Comparison between the predicted and experimentally verified AsCas12a *trans*-cleavage activity. All data were presented as mean ± SD (n = 3). RFU: relative fluorescence units.

**Table S1. crRNA sequences for constructing crRNA-DNA dataset (Group 1&2)**

| **Name** | **Sequence (5’ to 3’)** |
| --- | --- |
| **crRNA sequences (Group 1)** | |
| L.ace_thrF1_crRNA1 | UAAUUUCUACUAAGUGUAGAUGUACAUGCCAAGCACAUUAC |
| L.ace_thrF1_crRNA2 | UAAUUUCUACUAAGUGUAGAUUCGGCAUAACUAGUUUUUCC |
| L.ace_aceF11_crRNA1 | UAAUUUCUACUAAGUGUAGAUGCCAAAACGGUGCGUAAUGU |
| L.ace_aceF11_crRNA2 | UAAUUUCUACUAAGUGUAGAUGCCGAGCGGUUAUUAUCACA |
| L.ace_aceF11_crRNA3 | UAAUUUCUACUAAGUGUAGAUGAAGUUUGGUACAUGCCAAG |
| L.ace_aceF11_crRNA4 | UAAUUUCUACUAAGUGUAGAUGCCAAGCACAUUACGCACCG |
| PAM crRNA-SARS-CoV2 | UAAUUUCUACUAAGUGUAGAUUGAUCUAUUUUAACCUUCCU |
| PAM-free crRNA1-SARS-CoV2 | UAAUUUCUACUAAGUGUAGAUGGCUUAGCUAAGAUAAGGGG |
| PAM-free crRNA2-SARS-CoV2 | UAAUUUCUACUAAGUGUAGAUGCCUACGAUAACGUCUCCCC |
| PAM-endF5-crRNA | UAAUUUCUACUAAGUGUAGAUACCAGUGUCAUAAGGGCACC |
| PAM-free-endF5-crRNA1 | UAAUUUCUACUAAGUGUAGAUUAACUGCCUUUGCCUUAGGC |
| PAM-free-endF5-crRNA2 | UAAUUUCUACUAAGUGUAGAUCCCCACGCUUUCGUGCCUAA |
| PAM-free-endF5-crRNA3 | UAAUUUCUACUAAGUGUAGAUGGAAUUCCAUGUGUAGCGGU |
| PAM-free-endF5-crRNA4 | UAAUUUCUACUAAGUGUAGAUCAUCACCGCCAUUGCCAGCC |
| PAM-free-endF5-crRNA5 | UAAUUUCUACUAAGUGUAGAUUGCUUUGCUGCUGCUUGACA |
| PAM-RggF1-crRNA | UAAUUUCUACUAAGUGUAGAUACGGUGUGAUCUUCCAGGGC |
| PAM-free-RggF1-crRNA1 | UAAUUUCUACUAAGUGUAGAUCAAGCCAGCUCUCCAAUAGG |
| PAM-free-RggF1-crRNA2 | UAAUUUCUACUAAGUGUAGAUUUCAGGUCGUAAACGAGCAC |
| PAM-PpxF3-crRNA | UAAUUUCUACUAAGUGUAGAUCUGAUUCUGGUACUAAUGGU |
| PAM-free-PpxF3-crRNA1 | UAAUUUCUACUAAGUGUAGAUACCAGUGUCAUAAGGGCACC |
| PAM-free-PpxF3-crRNA2 | UAAUUUCUACUAAGUGUAGAUUAACUGCCUUUGCCUUAGGC |
| PAM-free-vp39-F3-crRNA1 | UAAUUUCUACUAAGUGUAGAUUUACUUACGUGAUCACCCAC |
| PAM-free-vp39-F3-crRNA2 | UAAUUUCUACUAAGUGUAGAUAAGAAGUUUUGGUUAAAGAU |
| PAM-free-S.eri-F5-crRNA1 | UAAUUUCUACUAAGUGUAGAUCUUACGUGAUCACCCACCGA |
| PAM-free-S.eri-F5-crRNA2 | UAAUUUCUACUAAGUGUAGAUAAAGAAGUUUUGGUUAAAGA |
| S.typ-1-F-PAM-free-crRNA1 | UAAUUUCUACUAAGUGUAGAUCUGCUGCUUGACAGAUUGAA |
| S.typ-1-F-PAM-free-crRNA2 | UAAUUUCUACUAAGUGUAGAUCAUCACCGCCAUUGCCAGCC |
| S.typ-2-F-PAM-free-crRNA1 | UAAUUUCUACUAAGUGUAGAUUGCUUUGCUGCUGCUUGACA |
| S.typ-2-F-PAM-free-crRNA2 | UAAUUUCUACUAAGUGUAGAUGUACAUGCCAAGCACAUUAC |
| S.typ-3-F-PAM-free-crRNA1 | UAAUUUCUACUAAGUGUAGAUUCGGCAUAACUAGUUUUUCC |
| S.typ-3-F-PAM-free-crRNA2 | UAAUUUCUACUAAGUGUAGAUGCCAAAACGGUGCGUAAUGU |
| Sac-PAM-free-crRNA1 | UAAUUUCUACUAAGUGUAGAUGCCGAGCGGUUAUUAUCACA |
| Sac-PAM-free-crRNA2 | UAAUUUCUACUAAGUGUAGAUGAAGUUUGGUACAUGCCAAG |
| Pk-1-F-PAM-free-crRNA1 | UAAUUUCUACUAAGUGUAGAUGCCAAGCACAUUACGCACCG |
| Pk-1-F-PAM-free-crRNA2 | UAAUUUCUACUAAGUGUAGAUACUGGAUAAAGCGGCAAAAG |
| Pk-2-F-PAM-free-crRNA1 | UAAUUUCUACUAAGUGUAGAUCCUUUUGCCGCUUUAUCCAG |
| Pk-2-F-PAM-free-crRNA2 | UAAUUUCUACUAAGUGUAGAUUCACCUUCAACGCUAUCGCG |
| Pk-3-F-PAM-free-crRNA1 | UAAUUUCUACUAAGUGUAGAUUGAUCUAUUUUAACCUUCCU |
| Pk-3-F-PAM-free-crRNA2 | UAAUUUCUACUAAGUGUAGAUGGCUUAGCUAAGAUAAGGGG |
| Sac-F-4-PAM-free-crRNA1 | UAAUUUCUACUAAGUGUAGAUGCCUACGAUAACGUCUCCCC |
| Sac-F-4-PAM-free-crRNA2 | UAAUUUCUACUAAGUGUAGAUGCAAUUGCCUAUUCGGUAGC |
| Sac-F-5-PAM-free-crRNA1 | UAAUUUCUACUAAGUGUAGAUGCGUCUUCGUAAAGAUGUUC |
| Sac-F-5-PAM-free-crRNA2 | UAAUUUCUACUAAGUGUAGAUCCCCACGCUUUCGUGCCUAA |
| Sac-F-6-PAM-free-crRNA1 | UAAUUUCUACUAAGUGUAGAUGGAAUUCCAUGUGUAGCGGU |
| Sac-F-6-PAM-free-crRNA2 | UAAUUUCUACUAAGUGUAGAUCCAGUACGAUAUUCAGUGCG |
| Sac-F-5-PAM-crRNA1 | UAAUUUCUACUAAGUGUAGAUCCACGCUCUUUCGUCUGGCA |
| Sac-F-5-PAM-crRNA2 | UAAUUUCUACUAAGUGUAGAUAUCGACGGACAUCGACAGAC |
| Pk-1-F-PAM-crRNA1 | UAAUUUCUACUAAGUGUAGAUGGUCGUUUUCCGGAUCUGGA |
| Pk-1-F-PAM-crRNA2 | UAAUUUCUACUAAGUGUAGAUUUCAGGUCGUAAACGAGCAC |
| InvA-RPA-F3-crRNA-1 | UAAUUUCUACUAAGUGUAGAUCUGAUUCUGGUACUAAUGGU |
| InvA-RPA-F5-crRNA-1 | UAAUUUCUACUAAGUGUAGAUCCCUGGAAUACCAAGGGGCG |
| InvA-RPA-F6-crRNA-1 | UAAUUUCUACUAAGUGUAGAUGCUGGCCUUUUCAUUGGAUG |
| WCX-crRNA 1 | UAAUUUCUACUAAGUGUAGAUACGGUGUGAUCUUCCAGGGC |
| WCX-crRNA 2 | UAAUUUCUACUAAGUGUAGAUCAAGCCAGCUCUCCAAUAGG |
| WCX-crRNA 3 | UAAUUUCUACUAAGUGUAGAUUUUCCCCACAUCGACAUCCA |
| WCX-crRNA 4 | UAAUUUCUACUAAGUGUAGAUCCCUGGAAGAUCACACCGUG |
| WCX-crRNA 5 | UAAUUUCUACUAAGUGUAGAUGAUCUUCCAGGGCUUCUUAC |
| WCX-crRNA 6 | UAAUUUCUACUAAGUGUAGAUGCCAGCUCUCCAAUAGGCAA |
| WCX-crRNA 7 | UAAUUUCUACUAAGUGUAGAUGGACAAUUAAAACCGUUUCA |
| WCX-crRNA 8 | UAAUUUCUACUAAGUGUAGAUCUCUCACCGUUUGGAAUAGC |
|  |  |
| **Name** | **Sequence (5’ to 3’)** |
| **crRNA sequences (Group 2)** | |
| Orf1ab spacer 4-crRNA | UAAUUUCUACUAAGUGUAGAUCCACAUAGAUCAUCCAAAUC |
| Orf1ab spacer 5-crRNA | UAAUUUCUACUAAGUGUAGAUACACAAUUAGUGAUUGGUUG |
| S gene spacer 2-crRNA | UAAUUUCUACUAAGUGUAGAUUCAGGUUGGACAGCUGGUGC |
| HPV18 L1 gene spacer 1-crRNA | UAAUUUCUACUAAGUGUAGAUCAACAGUUAAUAAUCUAGAG |

**Table S2. Short target sequences for constructing crRNA-DNA dataset (Group 1&2)**

| **Name** | **Sequence (5’ to 3’)** |
| --- | --- |
| **Target sequence (Group 1)** | |
| thrF1-crRNA1-TS | GGTGCCCTTATGACACTGGTGCCT |
| thrF1-crRNA2-TS | GCCTAAGGCAAAGGCAGTTAACGC |
| aceF11-crRNA1-TS | GTGGGTGATCACGTAAGTAAAGGC |
| aceF11-crRNA2-TS | ATCTTTAACCAAAACTTCTTTGAT |
| aceF11-crRNA3-TS | TCGGTGGGTGATCACGTAAGTAAA |
| aceF11-crRNA4-TS | TCTTTAACCAAAACTTCTTTGATT |
| SARS-PAM-TS | TTCAATCTGTCAAGCAGCAGCAAA |
| SARS-Pfree-1-TS | GGCTGGCAATGGCGGTGATGCTGC |
| SARS-Pfree-2-TS | TGTCAAGCAGCAGCAAAGCAAGAG |
| endF5-PAM-TS | GTAATGTGCTTGGCATGTACCAAA |
| endF5-Pfree-1-TS | GGAAAAACTAGTTATGCCGAGCGG |
| endF5-Pfree-2-TS | ACATTACGCACCGTTTTGGCTTTA |
| endF5-Pfree-3-TS | TGTGATAATAACCGCTCGGCATAA |
| endF5-Pfree-4-TS | CTTGGCATGTACCAAACTTCTTTT |
| endF5-Pfree-5-TS | CGGTGCGTAATGTGCTTGGCATGT |
| RggF1-PAM-TS | CTTTTGCCGCTTTATCCAGTGAAA |
| RggF1-Pfree-1-TS | CTGGATAAAGCGGCAAAAGGAATT |
| RggF1-Pfree-2-TS | CGCGATAGCGTTGAAGGTGAAACA |
| PpxF3-PAM-TS | AGGAAGGTTAAAATAGATCAGAAA |
| PpxF3-Pfree-1-TS | CCCCTTATCTTAGCTAAGCCTGGA |
| PpxF3-Pfree-2-TS | GGGGAGACGTTATCGTAGGCGGCT |
| vp39-F3-Pfree-1-TS | GCTACCGAATAGGCAATTGCACCT |
| vp39-F3-Pfree-2-TS | GAACATCTTTACGAAGACGCTTTC |
| Seri-F5-Pfree-1-TS | TTAGGCACGAAAGCGTGGGGAGCA |
| Seri-F5-Pfree-2-TS | ACCGCTACACATGGAATTCCATCA |
| StypF1-Pfree-1-TS | CGCACTGAATATCGTACTGGCGAT |
| StypF1-Pfree-2-TS | TGCCAGACGAAAGAGCGTGGTAAT |
| StypF2-Pfree-1-TS | GTCTGTCGATGTCCGTCGATTTAT |
| StypF2-Pfree-2-TS | TCCAGATCCGGAAAACGACCTTCA |
| StypF3-Pfree-1-TS | GTGCTCGTTTACGACCTGAATTAC |
| StypF3-Pfree-2-TS | ACCATTAGTACCAGAATCAGTAAT |
| Sac-Pfree-1-TS | CGCCCCTTGGTATTCCAGGGGGCA |
| Sac-Pfree-2-TS | CATCCAATGAAAAGGCCAGCAATT |
| PkF1-Pfree-1-TS | GCCCTGGAAGATCACACCGTGGAC |
| PkF1-Pfree-2-TS | CCTATTGGAGAGCTGGCTTGGATA |
| PkF2-Pfree-1-TS | TGGATGTCGATGTGGGGAAAATCA |
| PkF2-Pfree-2-TS | CACGGTGTGATCTTCCAGGGCTTC |
| PkF3-Pfree-1-TS | GTAAGAAGCCCTGGAAGATCACAC |
| PkF3-Pfree-2-TS | TTGCCTATTGGAGAGCTGGCTTGG |
| SacF4-Pfree-1-TS | TGAAACGGTTTTAATTGTCCTATA |
| SacF4-Pfree-2-TS | GCTATTCCAAACGGTGAGAGATTT |
| SacF5-Pfree-1-TS | GAAAACGCTCTGAAGGGTAATTTG |
| SacF5-Pfree-2-TS | CCTACACAACCTAAAATCACTTAG |
| SacF6-Pfree-1-TS | CTGTACTTGTTCGCTATCGGTCTC |
| SacF6-Pfree-2-TS | CTAAGTGGGTGGTAAATTCCATCT |
| SacF5-PAM-1-TS | ACCCTTCAGAGCGTTTTCTCTAAA |
| SacF5-PAM-2-TS | GTGTAGGTATCAATTTAGAGAAAA |
| PkF1-PAM-1-TS | GAATTGGATGTCGATGTGGGGAAA |
| PkF1-PAM-2-TS | CAAGCCAGCTCTCCAATAGGCAAA |
| InvAF3-PAM-1-TS | TGATTGAAGCCGATGCCGGTGAAA |
| InvAF5-PAM-1-TS | GATGTCCGTCGATTTATTAAGAAA |
| InvAF6-PAM-1-TS | TGATTGAAGCCGATGCCGGTGAAA |
| WCX-1-TS | CAGAGCCAGCTCAACCGTTTCAAA |
| WCX-2-TS | ACGGTTGAGCTGGCTCTGAGTAAA |
| WCX-3-TS | TTCGGTATTACGCAGTTACGGAAA |
| WCX-4-TS | TTGGTGAGCGGACTAGGCAAGAAA |
| WCX-5-TS | AGTGGAAACGTCCGGCGTGTAAAA |
| WCX-6-TS | GTGGTTACGGGGGGCCTGTATAAA |
| WCX-7-TS | ATCAGGGGAACGGTCCGGTGAAAA |
| WCX-8-TS | TAGTGGAAACGTCCGGCGTGTAAA |
| thrF1-crRNA1-TS | GGTGCCCTTATGACACTGGTGCCT |
| thrF1-crRNA2-TS | GCCTAAGGCAAAGGCAGTTAACGC |
| aceF11-crRNA1-TS | GTGGGTGATCACGTAAGTAAAGGC |
| aceF11-crRNA2-TS | ATCTTTAACCAAAACTTCTTTGAT |
| aceF11-crRNA3-TS | TCGGTGGGTGATCACGTAAGTAAA |
| aceF11-crRNA4-TS | TCTTTAACCAAAACTTCTTTGATT |
| SARS-PAM-TS | TTCAATCTGTCAAGCAGCAGCAAA |
| SARS-Pfree-1-TS | GGCTGGCAATGGCGGTGATGCTGC |
| SARS-Pfree-2-TS | TGTCAAGCAGCAGCAAAGCAAGAG |
| endF5-PAM-TS | GTAATGTGCTTGGCATGTACCAAA |
| endF5-Pfree-1-TS | GGAAAAACTAGTTATGCCGAGCGG |
| endF5-Pfree-2-TS | ACATTACGCACCGTTTTGGCTTTA |
| endF5-Pfree-3-TS | TGTGATAATAACCGCTCGGCATAA |
| endF5-Pfree-4-TS | CTTGGCATGTACCAAACTTCTTTT |
| endF5-Pfree-5-TS | CGGTGCGTAATGTGCTTGGCATGT |
| RggF1-PAM-TS | CTTTTGCCGCTTTATCCAGTGAAA |
| RggF1-Pfree-1-TS | CTGGATAAAGCGGCAAAAGGAATT |
| RggF1-Pfree-2-TS | CGCGATAGCGTTGAAGGTGAAACA |
| PpxF3-PAM-TS | AGGAAGGTTAAAATAGATCAGAAA |
| PpxF3-Pfree-1-TS | CCCCTTATCTTAGCTAAGCCTGGA |
| PpxF3-Pfree-2-TS | GGGGAGACGTTATCGTAGGCGGCT |
| vp39-F3-Pfree-1-TS | GCTACCGAATAGGCAATTGCACCT |
| vp39-F3-Pfree-2-TS | GAACATCTTTACGAAGACGCTTTC |
| Seri-F5-Pfree-1-TS | TTAGGCACGAAAGCGTGGGGAGCA |
| Seri-F5-Pfree-2-TS | ACCGCTACACATGGAATTCCATCA |
| StypF1-Pfree-1-TS | CGCACTGAATATCGTACTGGCGAT |
| StypF1-Pfree-2-TS | TGCCAGACGAAAGAGCGTGGTAAT |
| StypF2-Pfree-1-TS | GTCTGTCGATGTCCGTCGATTTAT |
| StypF2-Pfree-2-TS | TCCAGATCCGGAAAACGACCTTCA |
| StypF3-Pfree-1-TS | GTGCTCGTTTACGACCTGAATTAC |
| StypF3-Pfree-2-TS | ACCATTAGTACCAGAATCAGTAAT |
| Sac-Pfree-1-TS | CGCCCCTTGGTATTCCAGGGGGCA |
| Sac-Pfree-2-TS | CATCCAATGAAAAGGCCAGCAATT |
| PkF1-Pfree-1-TS | GCCCTGGAAGATCACACCGTGGAC |
| PkF1-Pfree-2-TS | CCTATTGGAGAGCTGGCTTGGATA |
| PkF2-Pfree-1-TS | TGGATGTCGATGTGGGGAAAATCA |
| PkF2-Pfree-2-TS | CACGGTGTGATCTTCCAGGGCTTC |
| PkF3-Pfree-1-TS | GTAAGAAGCCCTGGAAGATCACAC |
| PkF3-Pfree-2-TS | TTGCCTATTGGAGAGCTGGCTTGG |
| SacF4-Pfree-1-TS | TGAAACGGTTTTAATTGTCCTATA |
| SacF4-Pfree-2-TS | GCTATTCCAAACGGTGAGAGATTT |
| SacF5-Pfree-1-TS | GAAAACGCTCTGAAGGGTAATTTG |
| SacF5-Pfree-2-TS | CCTACACAACCTAAAATCACTTAG |
| SacF6-Pfree-1-TS | CTGTACTTGTTCGCTATCGGTCTC |
| SacF6-Pfree-2-TS | CTAAGTGGGTGGTAAATTCCATCT |
| SacF5-PAM-1-TS | ACCCTTCAGAGCGTTTTCTCTAAA |
| SacF5-PAM-2-TS | GTGTAGGTATCAATTTAGAGAAAA |
| PkF1-PAM-1-TS | GAATTGGATGTCGATGTGGGGAAA |
| PkF1-PAM-2-TS | CAAGCCAGCTCTCCAATAGGCAAA |
| InvAF3-PAM-1-TS | TGATTGAAGCCGATGCCGGTGAAA |
| InvAF5-PAM-1-TS | GATGTCCGTCGATTTATTAAGAAA |
| InvAF6-PAM-1-TS | TGATTGAAGCCGATGCCGGTGAAA |
| WCX-1-TS | CAGAGCCAGCTCAACCGTTTCAAA |
| WCX-2-TS | ACGGTTGAGCTGGCTCTGAGTAAA |
| WCX-3-TS | TTCGGTATTACGCAGTTACGGAAA |
| WCX-4-TS | TTGGTGAGCGGACTAGGCAAGAAA |
| WCX-5-TS | AGTGGAAACGTCCGGCGTGTAAAA |
| WCX-6-TS | GTGGTTACGGGGGGCCTGTATAAA |
| WCX-7-TS | ATCAGGGGAACGGTCCGGTGAAAA |
| WCX-8-TS | TAGTGGAAACGTCCGGCGTGTAAA |
| thrF1-crRNA1-TS | GGTGCCCTTATGACACTGGTGCCT |
| thrF1-crRNA2-TS | GCCTAAGGCAAAGGCAGTTAACGC |
| aceF11-crRNA1-TS | GTGGGTGATCACGTAAGTAAAGGC |
| aceF11-crRNA2-TS | ATCTTTAACCAAAACTTCTTTGAT |
| aceF11-crRNA3-TS | TCGGTGGGTGATCACGTAAGTAAA |
| aceF11-crRNA4-TS | TCTTTAACCAAAACTTCTTTGATT |
| SARS-PAM-TS | TTCAATCTGTCAAGCAGCAGCAAA |
| SARS-Pfree-1-TS | GGCTGGCAATGGCGGTGATGCTGC |
| SARS-Pfree-2-TS | TGTCAAGCAGCAGCAAAGCAAGAG |
| endF5-PAM-TS | GTAATGTGCTTGGCATGTACCAAA |
| endF5-Pfree-1-TS | GGAAAAACTAGTTATGCCGAGCGG |
| endF5-Pfree-2-TS | ACATTACGCACCGTTTTGGCTTTA |
| endF5-Pfree-3-TS | TGTGATAATAACCGCTCGGCATAA |
| endF5-Pfree-4-TS | CTTGGCATGTACCAAACTTCTTTT |
| endF5-Pfree-5-TS | CGGTGCGTAATGTGCTTGGCATGT |
| RggF1-PAM-TS | CTTTTGCCGCTTTATCCAGTGAAA |
| RggF1-Pfree-1-TS | CTGGATAAAGCGGCAAAAGGAATT |
| RggF1-Pfree-2-TS | CGCGATAGCGTTGAAGGTGAAACA |
| PpxF3-PAM-TS | AGGAAGGTTAAAATAGATCAGAAA |
| PpxF3-Pfree-1-TS | CCCCTTATCTTAGCTAAGCCTGGA |
| PpxF3-Pfree-2-TS | GGGGAGACGTTATCGTAGGCGGCT |
| vp39-F3-Pfree-1-TS | GCTACCGAATAGGCAATTGCACCT |
| vp39-F3-Pfree-2-TS | GAACATCTTTACGAAGACGCTTTC |
| Seri-F5-Pfree-1-TS | TTAGGCACGAAAGCGTGGGGAGCA |
| Seri-F5-Pfree-2-TS | ACCGCTACACATGGAATTCCATCA |
| StypF1-Pfree-1-TS | CGCACTGAATATCGTACTGGCGAT |
| StypF1-Pfree-2-TS | TGCCAGACGAAAGAGCGTGGTAAT |
| StypF2-Pfree-1-TS | GTCTGTCGATGTCCGTCGATTTAT |
| StypF2-Pfree-2-TS | TCCAGATCCGGAAAACGACCTTCA |
| StypF3-Pfree-1-TS | GTGCTCGTTTACGACCTGAATTAC |
| StypF3-Pfree-2-TS | ACCATTAGTACCAGAATCAGTAAT |
| Sac-Pfree-1-TS | CGCCCCTTGGTATTCCAGGGGGCA |
| Sac-Pfree-2-TS | CATCCAATGAAAAGGCCAGCAATT |
| PkF1-Pfree-1-TS | GCCCTGGAAGATCACACCGTGGAC |
| PkF1-Pfree-2-TS | CCTATTGGAGAGCTGGCTTGGATA |
| PkF2-Pfree-1-TS | TGGATGTCGATGTGGGGAAAATCA |
| PkF2-Pfree-2-TS | CACGGTGTGATCTTCCAGGGCTTC |
| PkF3-Pfree-1-TS | GTAAGAAGCCCTGGAAGATCACAC |
| PkF3-Pfree-2-TS | TTGCCTATTGGAGAGCTGGCTTGG |
| SacF4-Pfree-1-TS | TGAAACGGTTTTAATTGTCCTATA |
| SacF4-Pfree-2-TS | GCTATTCCAAACGGTGAGAGATTT |
| SacF5-Pfree-1-TS | GAAAACGCTCTGAAGGGTAATTTG |
| SacF5-Pfree-2-TS | CCTACACAACCTAAAATCACTTAG |
| SacF6-Pfree-1-TS | CTGTACTTGTTCGCTATCGGTCTC |
| SacF6-Pfree-2-TS | CTAAGTGGGTGGTAAATTCCATCT |
| SacF5-PAM-1-TS | ACCCTTCAGAGCGTTTTCTCTAAA |
| SacF5-PAM-2-TS | GTGTAGGTATCAATTTAGAGAAAA |
| PkF1-PAM-1-TS | GAATTGGATGTCGATGTGGGGAAA |
| PkF1-PAM-2-TS | CAAGCCAGCTCTCCAATAGGCAAA |
| InvAF3-PAM-1-TS | TGATTGAAGCCGATGCCGGTGAAA |
| InvAF5-PAM-1-TS | GATGTCCGTCGATTTATTAAGAAA |
| InvAF6-PAM-1-TS | TGATTGAAGCCGATGCCGGTGAAA |
| WCX-1-TS | CAGAGCCAGCTCAACCGTTTCAAA |
| WCX-2-TS | ACGGTTGAGCTGGCTCTGAGTAAA |
| WCX-3-TS | TTCGGTATTACGCAGTTACGGAAA |
| WCX-4-TS | TTGGTGAGCGGACTAGGCAAGAAA |
| WCX-5-TS | AGTGGAAACGTCCGGCGTGTAAAA |
| WCX-6-TS | GTGGTTACGGGGGGCCTGTATAAA |
| WCX-7-TS | ATCAGGGGAACGGTCCGGTGAAAA |
| WCX-8-TS | TAGTGGAAACGTCCGGCGTGTAAA |
| thrF1-crRNA1-NTS | AGGCACCAGTGTCATAAGGGCACC |
| thrF1-crRNA2-NTS | GCGTTAACTGCCTTTGCCTTAGGC |
| aceF11-crRNA1-NTS | GCCTTTACTTACGTGATCACCCAC |
| aceF11-crRNA2-NTS | ATCAAAGAAGTTTTGGTTAAAGAT |
| aceF11-crRNA3-NTS | TTTACTTACGTGATCACCCACCGA |
| aceF11-crRNA4-NTS | AATCAAAGAAGTTTTGGTTAAAGA |
| SARS-PAM-NTS | TTTGCTGCTGCTTGACAGATTGAA |
| SARS-Pfree-1-NTS | GCAGCATCACCGCCATTGCCAGCC |
| SARS-Pfree-2-NTS | CTCTTGCTTTGCTGCTGCTTGACA |
| endF5-PAM-NTS | TTTGGTACATGCCAAGCACATTAC |
| endF5-Pfree-1-NTS | CCGCTCGGCATAACTAGTTTTTCC |
| endF5-Pfree-2-NTS | TAAAGCCAAAACGGTGCGTAATGT |
| endF5-Pfree-3-NTS | TTATGCCGAGCGGTTATTATCACA |
| endF5-Pfree-4-NTS | AAAAGAAGTTTGGTACATGCCAAG |
| endF5-Pfree-5-NTS | ACATGCCAAGCACATTACGCACCG |
| RggF1-PAM-NTS | TTTCACTGGATAAAGCGGCAAAAG |
| RggF1-Pfree-1-NTS | AATTCCTTTTGCCGCTTTATCCAG |
| RggF1-Pfree-2-NTS | TGTTTCACCTTCAACGCTATCGCG |
| PpxF3-PAM-NTS | TTTCTGATCTATTTTAACCTTCCT |
| PpxF3-Pfree-1-NTS | TCCAGGCTTAGCTAAGATAAGGGG |
| PpxF3-Pfree-2-NTS | AGCCGCCTACGATAACGTCTCCCC |
| vp39-F3-Pfree-1-NTS | AGGTGCAATTGCCTATTCGGTAGC |
| vp39-F3-Pfree-2-NTS | GAAAGCGTCTTCGTAAAGATGTTC |
| Seri-F5-Pfree-1-NTS | TGCTCCCCACGCTTTCGTGCCTAA |
| Seri-F5-Pfree-2-NTS | TGATGGAATTCCATGTGTAGCGGT |
| StypF1-Pfree-1-NTS | ATCGCCAGTACGATATTCAGTGCG |
| StypF1-Pfree-2-NTS | ATTACCACGCTCTTTCGTCTGGCA |
| StypF2-Pfree-1-NTS | ATAAATCGACGGACATCGACAGAC |
| StypF2-Pfree-2-NTS | TGAAGGTCGTTTTCCGGATCTGGA |
| StypF3-Pfree-1-NTS | GTAATTCAGGTCGTAAACGAGCAC |
| StypF3-Pfree-2-NTS | ATTACTGATTCTGGTACTAATGGT |
| Sac-Pfree-1-NTS | TGCCCCCTGGAATACCAAGGGGCG |
| Sac-Pfree-2-NTS | AATTGCTGGCCTTTTCATTGGATG |
| PkF1-Pfree-1-NTS | GTCCACGGTGTGATCTTCCAGGGC |
| PkF1-Pfree-2-NTS | TATCCAAGCCAGCTCTCCAATAGG |
| PkF2-Pfree-1-NTS | TGATTTTCCCCACATCGACATCCA |
| PkF2-Pfree-2-NTS | GAAGCCCTGGAAGATCACACCGTG |
| PkF3-Pfree-1-NTS | GTGTGATCTTCCAGGGCTTCTTAC |
| PkF3-Pfree-2-NTS | CCAAGCCAGCTCTCCAATAGGCAA |
| SacF4-Pfree-1-NTS | TATAGGACAATTAAAACCGTTTCA |
| SacF4-Pfree-2-NTS | AAATCTCTCACCGTTTGGAATAGC |
| SacF5-Pfree-1-NTS | CAAATTACCCTTCAGAGCGTTTTC |
| SacF5-Pfree-2-NTS | CTAAGTGATTTTAGGTTGTGTAGG |
| SacF6-Pfree-1-NTS | GAGACCGATAGCGAACAAGTACAG |
| SacF6-Pfree-2-NTS | AGATGGAATTTACCACCCACTTAG |
| SacF5-PAM-1-NTS | TTTAGAGAAAACGCTCTGAAGGGT |
| SacF5-PAM-2-NTS | TTTTCTCTAAATTGATACCTACAC |
| PkF1-PAM-1-NTS | TTTCCCCACATCGACATCCAATTC |
| PkF1-PAM-2-NTS | TTTGCCTATTGGAGAGCTGGCTTG |
| InvAF3-PAM-1-NTS | TTTCACCGGCATCGGCTTCAATCA |
| InvAF5-PAM-1-NTS | TTTCTTAATAAATCGACGGACATC |
| InvAF6-PAM-1-NTS | TTTCACCGGCATCGGCTTCAATCA |
| WCX-1-NTS | TTTGAAACGGTTGAGCTGGCTCTG |
| WCX-2-NTS | TTTACTCAGAGCCAGCTCAACCGT |
| WCX-3-NTS | TTTCCGTAACTGCGTAATACCGAA |
| WCX-4-NTS | TTTCTTGCCTAGTCCGCTCACCAA |
| WCX-5-NTS | TTTTACACGCCGGACGTTTCCACT |
| WCX-6-NTS | TTTATACAGGCCCCCCGTAACCAC |
| WCX-7-NTS | TTTTCACCGGACCGTTCCCCTGAT |
| WCX-8-NTS | TTTACACGCCGGACGTTTCCACTA |
| thrF1-crRNA1-NTS | AGGCACCAGTGTCATAAGGGCACC |
| thrF1-crRNA2-NTS | GCGTTAACTGCCTTTGCCTTAGGC |
| aceF11-crRNA1-NTS | GCCTTTACTTACGTGATCACCCAC |
| aceF11-crRNA2-NTS | ATCAAAGAAGTTTTGGTTAAAGAT |
| aceF11-crRNA3-NTS | TTTACTTACGTGATCACCCACCGA |
| aceF11-crRNA4-NTS | AATCAAAGAAGTTTTGGTTAAAGA |
| SARS-PAM-NTS | TTTGCTGCTGCTTGACAGATTGAA |
| SARS-Pfree-1-NTS | GCAGCATCACCGCCATTGCCAGCC |
| SARS-Pfree-2-NTS | CTCTTGCTTTGCTGCTGCTTGACA |
| endF5-PAM-NTS | TTTGGTACATGCCAAGCACATTAC |
| endF5-Pfree-1-NTS | CCGCTCGGCATAACTAGTTTTTCC |
| endF5-Pfree-2-NTS | TAAAGCCAAAACGGTGCGTAATGT |
| endF5-Pfree-3-NTS | TTATGCCGAGCGGTTATTATCACA |
| endF5-Pfree-4-NTS | AAAAGAAGTTTGGTACATGCCAAG |
| endF5-Pfree-5-NTS | ACATGCCAAGCACATTACGCACCG |
| RggF1-PAM-NTS | TTTCACTGGATAAAGCGGCAAAAG |
| RggF1-Pfree-1-NTS | AATTCCTTTTGCCGCTTTATCCAG |
| RggF1-Pfree-2-NTS | TGTTTCACCTTCAACGCTATCGCG |
| PpxF3-PAM-NTS | TTTCTGATCTATTTTAACCTTCCT |
| PpxF3-Pfree-1-NTS | TCCAGGCTTAGCTAAGATAAGGGG |
| PpxF3-Pfree-2-NTS | AGCCGCCTACGATAACGTCTCCCC |
| vp39-F3-Pfree-1-NTS | AGGTGCAATTGCCTATTCGGTAGC |
| vp39-F3-Pfree-2-NTS | GAAAGCGTCTTCGTAAAGATGTTC |
| Seri-F5-Pfree-1-NTS | TGCTCCCCACGCTTTCGTGCCTAA |
| Seri-F5-Pfree-2-NTS | TGATGGAATTCCATGTGTAGCGGT |
| StypF1-Pfree-1-NTS | ATCGCCAGTACGATATTCAGTGCG |
| StypF1-Pfree-2-NTS | ATTACCACGCTCTTTCGTCTGGCA |
| StypF2-Pfree-1-NTS | ATAAATCGACGGACATCGACAGAC |
| StypF2-Pfree-2-NTS | TGAAGGTCGTTTTCCGGATCTGGA |
| StypF3-Pfree-1-NTS | GTAATTCAGGTCGTAAACGAGCAC |
| StypF3-Pfree-2-NTS | ATTACTGATTCTGGTACTAATGGT |
| Sac-Pfree-1-NTS | TGCCCCCTGGAATACCAAGGGGCG |
| Sac-Pfree-2-NTS | AATTGCTGGCCTTTTCATTGGATG |
| PkF1-Pfree-1-NTS | GTCCACGGTGTGATCTTCCAGGGC |
| PkF1-Pfree-2-NTS | TATCCAAGCCAGCTCTCCAATAGG |
| PkF2-Pfree-1-NTS | TGATTTTCCCCACATCGACATCCA |
| PkF2-Pfree-2-NTS | GAAGCCCTGGAAGATCACACCGTG |
| PkF3-Pfree-1-NTS | GTGTGATCTTCCAGGGCTTCTTAC |
| PkF3-Pfree-2-NTS | CCAAGCCAGCTCTCCAATAGGCAA |
| SacF4-Pfree-1-NTS | TATAGGACAATTAAAACCGTTTCA |
| SacF4-Pfree-2-NTS | AAATCTCTCACCGTTTGGAATAGC |
| SacF5-Pfree-1-NTS | CAAATTACCCTTCAGAGCGTTTTC |
| SacF5-Pfree-2-NTS | CTAAGTGATTTTAGGTTGTGTAGG |
| SacF6-Pfree-1-NTS | GAGACCGATAGCGAACAAGTACAG |
| SacF6-Pfree-2-NTS | AGATGGAATTTACCACCCACTTAG |
| SacF5-PAM-1-NTS | TTTAGAGAAAACGCTCTGAAGGGT |
| SacF5-PAM-2-NTS | TTTTCTCTAAATTGATACCTACAC |
| PkF1-PAM-1-NTS | TTTCCCCACATCGACATCCAATTC |
| PkF1-PAM-2-NTS | TTTGCCTATTGGAGAGCTGGCTTG |
| InvAF3-PAM-1-NTS | TTTCACCGGCATCGGCTTCAATCA |
| InvAF5-PAM-1-NTS | TTTCTTAATAAATCGACGGACATC |
| InvAF6-PAM-1-NTS | TTTCACCGGCATCGGCTTCAATCA |
| WCX-1-NTS | TTTGAAACGGTTGAGCTGGCTCTG |
| WCX-2-NTS | TTTACTCAGAGCCAGCTCAACCGT |
| WCX-3-NTS | TTTCCGTAACTGCGTAATACCGAA |
| WCX-4-NTS | TTTCTTGCCTAGTCCGCTCACCAA |
| WCX-5-NTS | TTTTACACGCCGGACGTTTCCACT |
| WCX-6-NTS | TTTATACAGGCCCCCCGTAACCAC |
| WCX-7-NTS | TTTTCACCGGACCGTTCCCCTGAT |
| WCX-8-NTS | TTTACACGCCGGACGTTTCCACTA |
| thrF1-crRNA1-NTS | AGGCACCAGTGTCATAAGGGCACC |
| thrF1-crRNA2-NTS | GCGTTAACTGCCTTTGCCTTAGGC |
| aceF11-crRNA1-NTS | GCCTTTACTTACGTGATCACCCAC |
| aceF11-crRNA2-NTS | ATCAAAGAAGTTTTGGTTAAAGAT |
| aceF11-crRNA3-NTS | TTTACTTACGTGATCACCCACCGA |
| aceF11-crRNA4-NTS | AATCAAAGAAGTTTTGGTTAAAGA |
| SARS-PAM-NTS | TTTGCTGCTGCTTGACAGATTGAA |
| SARS-Pfree-1-NTS | GCAGCATCACCGCCATTGCCAGCC |
| SARS-Pfree-2-NTS | CTCTTGCTTTGCTGCTGCTTGACA |
| endF5-PAM-NTS | TTTGGTACATGCCAAGCACATTAC |
| endF5-Pfree-1-NTS | CCGCTCGGCATAACTAGTTTTTCC |
| endF5-Pfree-2-NTS | TAAAGCCAAAACGGTGCGTAATGT |
| endF5-Pfree-3-NTS | TTATGCCGAGCGGTTATTATCACA |
| endF5-Pfree-4-NTS | AAAAGAAGTTTGGTACATGCCAAG |
| endF5-Pfree-5-NTS | ACATGCCAAGCACATTACGCACCG |
| RggF1-PAM-NTS | TTTCACTGGATAAAGCGGCAAAAG |
| RggF1-Pfree-1-NTS | AATTCCTTTTGCCGCTTTATCCAG |
| RggF1-Pfree-2-NTS | TGTTTCACCTTCAACGCTATCGCG |
| PpxF3-PAM-NTS | TTTCTGATCTATTTTAACCTTCCT |
| PpxF3-Pfree-1-NTS | TCCAGGCTTAGCTAAGATAAGGGG |
| PpxF3-Pfree-2-NTS | AGCCGCCTACGATAACGTCTCCCC |
| vp39-F3-Pfree-1-NTS | AGGTGCAATTGCCTATTCGGTAGC |
| vp39-F3-Pfree-2-NTS | GAAAGCGTCTTCGTAAAGATGTTC |
| Seri-F5-Pfree-1-NTS | TGCTCCCCACGCTTTCGTGCCTAA |
| Seri-F5-Pfree-2-NTS | TGATGGAATTCCATGTGTAGCGGT |
| StypF1-Pfree-1-NTS | ATCGCCAGTACGATATTCAGTGCG |
| StypF1-Pfree-2-NTS | ATTACCACGCTCTTTCGTCTGGCA |
| StypF2-Pfree-1-NTS | ATAAATCGACGGACATCGACAGAC |
| StypF2-Pfree-2-NTS | TGAAGGTCGTTTTCCGGATCTGGA |
| StypF3-Pfree-1-NTS | GTAATTCAGGTCGTAAACGAGCAC |
| StypF3-Pfree-2-NTS | ATTACTGATTCTGGTACTAATGGT |
| Sac-Pfree-1-NTS | TGCCCCCTGGAATACCAAGGGGCG |
| Sac-Pfree-2-NTS | AATTGCTGGCCTTTTCATTGGATG |
| PkF1-Pfree-1-NTS | GTCCACGGTGTGATCTTCCAGGGC |
| PkF1-Pfree-2-NTS | TATCCAAGCCAGCTCTCCAATAGG |
| PkF2-Pfree-1-NTS | TGATTTTCCCCACATCGACATCCA |
| PkF2-Pfree-2-NTS | GAAGCCCTGGAAGATCACACCGTG |
| PkF3-Pfree-1-NTS | GTGTGATCTTCCAGGGCTTCTTAC |
| PkF3-Pfree-2-NTS | CCAAGCCAGCTCTCCAATAGGCAA |
| SacF4-Pfree-1-NTS | TATAGGACAATTAAAACCGTTTCA |
| SacF4-Pfree-2-NTS | AAATCTCTCACCGTTTGGAATAGC |
| SacF5-Pfree-1-NTS | CAAATTACCCTTCAGAGCGTTTTC |
| SacF5-Pfree-2-NTS | CTAAGTGATTTTAGGTTGTGTAGG |
| SacF6-Pfree-1-NTS | GAGACCGATAGCGAACAAGTACAG |
| SacF6-Pfree-2-NTS | AGATGGAATTTACCACCCACTTAG |
| SacF5-PAM-1-NTS | TTTAGAGAAAACGCTCTGAAGGGT |
| SacF5-PAM-2-NTS | TTTTCTCTAAATTGATACCTACAC |
| PkF1-PAM-1-NTS | TTTCCCCACATCGACATCCAATTC |
| PkF1-PAM-2-NTS | TTTGCCTATTGGAGAGCTGGCTTG |
| InvAF3-PAM-1-NTS | TTTCACCGGCATCGGCTTCAATCA |
| InvAF5-PAM-1-NTS | TTTCTTAATAAATCGACGGACATC |
| InvAF6-PAM-1-NTS | TTTCACCGGCATCGGCTTCAATCA |
| WCX-1-NTS | TTTGAAACGGTTGAGCTGGCTCTG |
| WCX-2-NTS | TTTACTCAGAGCCAGCTCAACCGT |
| WCX-3-NTS | TTTCCGTAACTGCGTAATACCGAA |
| WCX-4-NTS | TTTCTTGCCTAGTCCGCTCACCAA |
| WCX-5-NTS | TTTTACACGCCGGACGTTTCCACT |
| WCX-6-NTS | TTTATACAGGCCCCCCGTAACCAC |
| WCX-7-NTS | TTTTCACCGGACCGTTCCCCTGAT |
| WCX-8-NTS | TTTACACGCCGGACGTTTCCACTA |
| **Target sequence (Group 2)** | |
| Orf-4-TS-1 | GATTTGGATGATCTATGTGGTAAA |
| Orf-4-TS-2 | GATTTGGATGATCTATGTGGTAAT |
| Orf-4-TS-3 | GATTTGGATGATCTATGTGGTAAC |
| Orf-4-TS-4 | GATTTGGATGATCTATGTGGTAAG |
| Orf-4-TS-5 | GATTTGGATGATCTATGTGGTATA |
| Orf-4-TS-6 | GATTTGGATGATCTATGTGGTACA |
| Orf-4-TS-7 | GATTTGGATGATCTATGTGGTAGA |
| Orf-4-TS-8 | GATTTGGATGATCTATGTGGTTAA |
| Orf-4-TS-9 | GATTTGGATGATCTATGTGGTCAA |
| Orf-4-TS-10 | GATTTGGATGATCTATGTGGTGAA |
| Orf-4-TS-11 | GATTTGGATGATCTATGTGGCAAA |
| Orf-4-TS-12 | GATTTGGATGATCTATGTGGCAAT |
| Orf-4-TS-13 | GATTTGGATGATCTATGTGGCAAC |
| Orf-4-TS-14 | GATTTGGATGATCTATGTGGCAAG |
| Orf-4-TS-15 | GATTTGGATGATCTATGTGGCATA |
| Orf-4-TS-16 | GATTTGGATGATCTATGTGGCACA |
| Orf-4-TS-17 | GATTTGGATGATCTATGTGGCAGA |
| Orf-4-TS-18 | GATTTGGATGATCTATGTGGCTAA |
| Orf-4-TS-19 | GATTTGGATGATCTATGTGGCCAA |
| Orf-4-TS-20 | GATTTGGATGATCTATGTGGCGAA |
| Orf-4-TS-21 | GATTTGGATGATCTATGTGGGAAA |
| Orf-4-TS-22 | GATTTGGATGATCTATGTGGGAAT |
| Orf-4-TS-23 | GATTTGGATGATCTATGTGGGAAC |
| Orf-4-TS-24 | GATTTGGATGATCTATGTGGGAAG |
| Orf-4-TS-25 | GATTTGGATGATCTATGTGGGATA |
| Orf-4-TS-26 | GATTTGGATGATCTATGTGGGACA |
| Orf-4-TS-27 | GATTTGGATGATCTATGTGGGAGA |
| Orf-4-TS-28 | GATTTGGATGATCTATGTGGGTAA |
| Orf-4-TS-29 | GATTTGGATGATCTATGTGGGCAA |
| Orf-4-TS-30 | GATTTGGATGATCTATGTGGGGAA |
| Orf-5-TS-1 | CAACCAATCACTAATTGTGTTAAA |
| Orf-5-TS-2 | CAACCAATCACTAATTGTGTTAAT |
| Orf-5-TS-3 | CAACCAATCACTAATTGTGTTAAG |
| Orf-5-TS-4 | CAACCAATCACTAATTGTGTTAAG |
| Orf-5-TS-5 | CAACCAATCACTAATTGTGTTATA |
| Orf-5-TS-6 | CAACCAATCACTAATTGTGTTACA |
| Orf-5-TS-7 | CAACCAATCACTAATTGTGTTAGA |
| Orf-5-TS-8 | CAACCAATCACTAATTGTGTTTAA |
| Orf-5-TS-9 | CAACCAATCACTAATTGTGTTCAA |
| Orf-5-TS-10 | CAACCAATCACTAATTGTGTTGAA |
| Orf-5-TS-11 | CAACCAATCACTAATTGTGTCAAA |
| Orf-5-TS-12 | CAACCAATCACTAATTGTGTCAAT |
| Orf-5-TS-13 | CAACCAATCACTAATTGTGTCAAC |
| Orf-5-TS-14 | CAACCAATCACTAATTGTGTCAAG |
| Orf-5-TS-15 | CAACCAATCACTAATTGTGTCATA |
| Orf-5-TS-16 | CAACCAATCACTAATTGTGTCACA |
| Orf-5-TS-17 | CAACCAATCACTAATTGTGTCAGA |
| Orf-5-TS-18 | CAACCAATCACTAATTGTGTCTAA |
| Orf-5-TS-19 | CAACCAATCACTAATTGTGTCCAA |
| Orf-5-TS-20 | CAACCAATCACTAATTGTGTCGAA |
| Orf-5-TS-21 | CAACCAATCACTAATTGTGTGAAA |
| Orf-5-TS-22 | CAACCAATCACTAATTGTGTGAAT |
| Orf-5-TS-23 | CAACCAATCACTAATTGTGTGAAC |
| Orf-5-TS-24 | CAACCAATCACTAATTGTGTGAAG |
| Orf-5-TS-25 | CAACCAATCACTAATTGTGTGATA |
| Orf-5-TS-26 | CAACCAATCACTAATTGTGTGACA |
| Orf-5-TS-27 | CAACCAATCACTAATTGTGTGAGA |
| Orf-5-TS-28 | CAACCAATCACTAATTGTGTGTAA |
| Orf-5-TS-29 | CAACCAATCACTAATTGTGTGCAA |
| Orf-5-TS-30 | CAACCAATCACTAATTGTGTGGAA |
| Sg-2-TS-1 | GCACCAGCTGTCCAACCTGATAAA |
| Sg-2-TS-2 | GCACCAGCTGTCCAACCTGATAAT |
| Sg-2-TS-3 | GCACCAGCTGTCCAACCTGATAAC |
| Sg-2-TS-4 | GCACCAGCTGTCCAACCTGATAAG |
| Sg-2-TS-5 | GCACCAGCTGTCCAACCTGATATA |
| Sg-2-TS-6 | GCACCAGCTGTCCAACCTGATACA |
| Sg-2-TS-7 | GCACCAGCTGTCCAACCTGATAGA |
| Sg-2-TS-8 | GCACCAGCTGTCCAACCTGATTAA |
| Sg-2-TS-9 | GCACCAGCTGTCCAACCTGATCAA |
| Sg-2-TS-10 | GCACCAGCTGTCCAACCTGATGAA |
| Sg-2-TS-11 | GCACCAGCTGTCCAACCTGACAAA |
| Sg-2-TS-12 | GCACCAGCTGTCCAACCTGACAAT |
| Sg-2-TS-13 | GCACCAGCTGTCCAACCTGACAAC |
| Sg-2-TS-14 | GCACCAGCTGTCCAACCTGACAAG |
| Sg-2-TS-15 | GCACCAGCTGTCCAACCTGACATA |
| Sg-2-TS-16 | GCACCAGCTGTCCAACCTGACACA |
| Sg-2-TS-17 | GCACCAGCTGTCCAACCTGACAGA |
| Sg-2-TS-18 | GCACCAGCTGTCCAACCTGACTAA |
| Sg-2-TS-19 | GCACCAGCTGTCCAACCTGACCAA |
| Sg-2-TS-20 | GCACCAGCTGTCCAACCTGACGAA |
| Sg-2-TS-21 | GCACCAGCTGTCCAACCTGAGAAA |
| Sg-2-TS-22 | GCACCAGCTGTCCAACCTGAGAAT |
| Sg-2-TS-23 | GCACCAGCTGTCCAACCTGAGAAC |
| Sg-2-TS-24 | GCACCAGCTGTCCAACCTGAGAAG |
| Sg-2-TS-25 | GCACCAGCTGTCCAACCTGAGATA |
| Sg-2-TS-26 | GCACCAGCTGTCCAACCTGAGACA |
| Sg-2-TS-27 | GCACCAGCTGTCCAACCTGAGAGA |
| Sg-2-TS-28 | GCACCAGCTGTCCAACCTGAGTAA |
| Sg-2-TS-29 | GCACCAGCTGTCCAACCTGAGCAA |
| Sg-2-TS-30 | GCACCAGCTGTCCAACCTGAGGAA |
| HL-1-TS-1 | CTCTAGATTATTAACTGTTGTAAA |
| HL-1-TS-2 | CTCTAGATTATTAACTGTTGTAAT |
| HL-1-TS-3 | CTCTAGATTATTAACTGTTGTAAC |
| HL-1-TS-4 | CTCTAGATTATTAACTGTTGTAAG |
| HL-1-TS-5 | CTCTAGATTATTAACTGTTGTATA |
| HL-1-TS-6 | CTCTAGATTATTAACTGTTGTACA |
| HL-1-TS-7 | CTCTAGATTATTAACTGTTGTAGA |
| HL-1-TS-8 | CTCTAGATTATTAACTGTTGTTAA |
| HL-1-TS-9 | CTCTAGATTATTAACTGTTGTCAA |
| HL-1-TS-10 | CTCTAGATTATTAACTGTTGTGAA |
| HL-1-TS-11 | CTCTAGATTATTAACTGTTGCAAA |
| HL-1-TS-12 | CTCTAGATTATTAACTGTTGCAAT |
| HL-1-TS-13 | CTCTAGATTATTAACTGTTGCAAC |
| HL-1-TS-14 | CTCTAGATTATTAACTGTTGCAAG |
| HL-1-TS-15 | CTCTAGATTATTAACTGTTGCATA |
| HL-1-TS-16 | CTCTAGATTATTAACTGTTGCACA |
| HL-1-TS-17 | CTCTAGATTATTAACTGTTGCAGA |
| HL-1-TS-18 | CTCTAGATTATTAACTGTTGCTAA |
| HL-1-TS-19 | CTCTAGATTATTAACTGTTGCCAA |
| HL-1-TS-20 | CTCTAGATTATTAACTGTTGCGAA |
| HL-1-TS-21 | CTCTAGATTATTAACTGTTGGAAA |
| HL-1-TS-22 | CTCTAGATTATTAACTGTTGGAAT |
| HL-1-TS-23 | CTCTAGATTATTAACTGTTGGAAC |
| HL-1-TS-24 | CTCTAGATTATTAACTGTTGGAAG |
| HL-1-TS-25 | CTCTAGATTATTAACTGTTGGATA |
| HL-1-TS-26 | CTCTAGATTATTAACTGTTGGACA |
| HL-1-TS-27 | CTCTAGATTATTAACTGTTGGAGA |
| HL-1-TS-28 | CTCTAGATTATTAACTGTTGGTAA |
| HL-1-TS-29 | CTCTAGATTATTAACTGTTGGCAA |
| HL-1-TS-30 | CTCTAGATTATTAACTGTTGGGAA |
| Orf-4-NTS-1 | TTTACCACATAGATCATCCAAATC |
| Orf-4-NTS-2 | ATTACCACATAGATCATCCAAATC |
| Orf-4-NTS-3 | GTTACCACATAGATCATCCAAATC |
| Orf-4-NTS-4 | CTTACCACATAGATCATCCAAATC |
| Orf-4-NTS-5 | TATACCACATAGATCATCCAAATC |
| Orf-4-NTS-6 | TGTACCACATAGATCATCCAAATC |
| Orf-4-NTS-7 | TCTACCACATAGATCATCCAAATC |
| Orf-4-NTS-8 | TTAACCACATAGATCATCCAAATC |
| Orf-4-NTS-9 | TTGACCACATAGATCATCCAAATC |
| Orf-4-NTS-10 | TTCACCACATAGATCATCCAAATC |
| Orf-4-NTS-11 | TTTGCCACATAGATCATCCAAATC |
| Orf-4-NTS-12 | ATTGCCACATAGATCATCCAAATC |
| Orf-4-NTS-13 | GTTGCCACATAGATCATCCAAATC |
| Orf-4-NTS-14 | CTTGCCACATAGATCATCCAAATC |
| Orf-4-NTS-15 | TATGCCACATAGATCATCCAAATC |
| Orf-4-NTS-16 | TGTGCCACATAGATCATCCAAATC |
| Orf-4-NTS-17 | TCTGCCACATAGATCATCCAAATC |
| Orf-4-NTS-18 | TTAGCCACATAGATCATCCAAATC |
| Orf-4-NTS-19 | TTGGCCACATAGATCATCCAAATC |
| Orf-4-NTS-20 | TTCGCCACATAGATCATCCAAATC |
| Orf-4-NTS-21 | TTTCCCACATAGATCATCCAAATC |
| Orf-4-NTS-22 | ATTCCCACATAGATCATCCAAATC |
| Orf-4-NTS-23 | GTTCCCACATAGATCATCCAAATC |
| Orf-4-NTS-24 | CTTCCCACATAGATCATCCAAATC |
| Orf-4-NTS-25 | TATCCCACATAGATCATCCAAATC |
| Orf-4-NTS-26 | TGTCCCACATAGATCATCCAAATC |
| Orf-4-NTS-27 | TCTCCCACATAGATCATCCAAATC |
| Orf-4-NTS-28 | TTACCCACATAGATCATCCAAATC |
| Orf-4-NTS-29 | TTGCCCACATAGATCATCCAAATC |
| Orf-4-NTS-30 | TTCCCCACATAGATCATCCAAATC |
| Orf-5-NTS-1 | TTTAACACAATTAGTGATTGGTTG |
| Orf-5-NTS-2 | ATTAACACAATTAGTGATTGGTTG |
| Orf-5-NTS-3 | CTTAACACAATTAGTGATTGGTTG |
| Orf-5-NTS-4 | CTTAACACAATTAGTGATTGGTTG |
| Orf-5-NTS-5 | TATAACACAATTAGTGATTGGTTG |
| Orf-5-NTS-6 | TGTAACACAATTAGTGATTGGTTG |
| Orf-5-NTS-7 | TCTAACACAATTAGTGATTGGTTG |
| Orf-5-NTS-8 | TTAAACACAATTAGTGATTGGTTG |
| Orf-5-NTS-9 | TTGAACACAATTAGTGATTGGTTG |
| Orf-5-NTS-10 | TTCAACACAATTAGTGATTGGTTG |
| Orf-5-NTS-11 | TTTGACACAATTAGTGATTGGTTG |
| Orf-5-NTS-12 | ATTGACACAATTAGTGATTGGTTG |
| Orf-5-NTS-13 | GTTGACACAATTAGTGATTGGTTG |
| Orf-5-NTS-14 | CTTGACACAATTAGTGATTGGTTG |
| Orf-5-NTS-15 | TATGACACAATTAGTGATTGGTTG |
| Orf-5-NTS-16 | TGTGACACAATTAGTGATTGGTTG |
| Orf-5-NTS-17 | TCTGACACAATTAGTGATTGGTTG |
| Orf-5-NTS-18 | TTAGACACAATTAGTGATTGGTTG |
| Orf-5-NTS-19 | TTGGACACAATTAGTGATTGGTTG |
| Orf-5-NTS-20 | TTCGACACAATTAGTGATTGGTTG |
| Orf-5-NTS-21 | TTTCACACAATTAGTGATTGGTTG |
| Orf-5-NTS-22 | ATTCACACAATTAGTGATTGGTTG |
| Orf-5-NTS-23 | GTTCACACAATTAGTGATTGGTTG |
| Orf-5-NTS-24 | CTTCACACAATTAGTGATTGGTTG |
| Orf-5-NTS-25 | TATCACACAATTAGTGATTGGTTG |
| Orf-5-NTS-26 | TGTCACACAATTAGTGATTGGTTG |
| Orf-5-NTS-27 | TCTCACACAATTAGTGATTGGTTG |
| Orf-5-NTS-28 | TTACACACAATTAGTGATTGGTTG |
| Orf-5-NTS-29 | TTGCACACAATTAGTGATTGGTTG |
| Orf-5-NTS-30 | TTCCACACAATTAGTGATTGGTTG |
| Sg-2-NTS-1 | TTTATCAGGTTGGACAGCTGGTGC |
| Sg-2-NTS-2 | ATTATCAGGTTGGACAGCTGGTGC |
| Sg-2-NTS-3 | GTTATCAGGTTGGACAGCTGGTGC |
| Sg-2-NTS-4 | CTTATCAGGTTGGACAGCTGGTGC |
| Sg-2-NTS-5 | TATATCAGGTTGGACAGCTGGTGC |
| Sg-2-NTS-6 | TGTATCAGGTTGGACAGCTGGTGC |
| Sg-2-NTS-7 | TCTATCAGGTTGGACAGCTGGTGC |
| Sg-2-NTS-8 | TTAATCAGGTTGGACAGCTGGTGC |
| Sg-2-NTS-9 | TTGATCAGGTTGGACAGCTGGTGC |
| Sg-2-NTS-10 | TTCATCAGGTTGGACAGCTGGTGC |
| Sg-2-NTS-11 | TTTGTCAGGTTGGACAGCTGGTGC |
| Sg-2-NTS-12 | ATTGTCAGGTTGGACAGCTGGTGC |
| Sg-2-NTS-13 | GTTGTCAGGTTGGACAGCTGGTGC |
| Sg-2-NTS-14 | CTTGTCAGGTTGGACAGCTGGTGC |
| Sg-2-NTS-15 | TATGTCAGGTTGGACAGCTGGTGC |
| Sg-2-NTS-16 | TGTGTCAGGTTGGACAGCTGGTGC |
| Sg-2-NTS-17 | TCTGTCAGGTTGGACAGCTGGTGC |
| Sg-2-NTS-18 | TTAGTCAGGTTGGACAGCTGGTGC |
| Sg-2-NTS-19 | TTGGTCAGGTTGGACAGCTGGTGC |
| Sg-2-NTS-20 | TTCGTCAGGTTGGACAGCTGGTGC |
| Sg-2-NTS-21 | TTTCTCAGGTTGGACAGCTGGTGC |
| Sg-2-NTS-22 | ATTCTCAGGTTGGACAGCTGGTGC |
| Sg-2-NTS-23 | GTTCTCAGGTTGGACAGCTGGTGC |
| Sg-2-NTS-24 | CTTCTCAGGTTGGACAGCTGGTGC |
| Sg-2-NTS-25 | TATCTCAGGTTGGACAGCTGGTGC |
| Sg-2-NTS-26 | TGTCTCAGGTTGGACAGCTGGTGC |
| Sg-2-NTS-27 | TCTCTCAGGTTGGACAGCTGGTGC |
| Sg-2-NTS-28 | TTACTCAGGTTGGACAGCTGGTGC |
| Sg-2-NTS-29 | TTGCTCAGGTTGGACAGCTGGTGC |
| Sg-2-NTS-30 | TTCCTCAGGTTGGACAGCTGGTGC |
| HL-1-NTS-1 | TTTACAACAGTTAATAATCTAGAG |
| HL-1-NTS-2 | ATTACAACAGTTAATAATCTAGAG |
| HL-1-NTS-3 | GTTACAACAGTTAATAATCTAGAG |
| HL-1-NTS-4 | CTTACAACAGTTAATAATCTAGAG |
| HL-1-NTS-5 | TATACAACAGTTAATAATCTAGAG |
| HL-1-NTS-6 | TGTACAACAGTTAATAATCTAGAG |
| HL-1-NTS-7 | TCTACAACAGTTAATAATCTAGAG |
| HL-1-NTS-8 | TTAACAACAGTTAATAATCTAGAG |
| HL-1-NTS-9 | TTGACAACAGTTAATAATCTAGAG |
| HL-1-NTS-10 | TTCACAACAGTTAATAATCTAGAG |
| HL-1-NTS-11 | TTTGCAACAGTTAATAATCTAGAG |
| HL-1-NTS-12 | ATTGCAACAGTTAATAATCTAGAG |
| HL-1-NTS-13 | GTTGCAACAGTTAATAATCTAGAG |
| HL-1-NTS-14 | CTTGCAACAGTTAATAATCTAGAG |
| HL-1-NTS-15 | TATGCAACAGTTAATAATCTAGAG |
| HL-1-NTS-16 | TGTGCAACAGTTAATAATCTAGAG |
| HL-1-NTS-17 | TCTGCAACAGTTAATAATCTAGAG |
| HL-1-NTS-18 | TTAGCAACAGTTAATAATCTAGAG |
| HL-1-NTS-19 | TTGGCAACAGTTAATAATCTAGAG |
| HL-1-NTS-20 | TTCGCAACAGTTAATAATCTAGAG |
| HL-1-NTS-21 | TTTCCAACAGTTAATAATCTAGAG |
| HL-1-NTS-22 | ATTCCAACAGTTAATAATCTAGAG |
| HL-1-NTS-23 | GTTCCAACAGTTAATAATCTAGAG |
| HL-1-NTS-24 | CTTCCAACAGTTAATAATCTAGAG |
| HL-1-NTS-25 | TATCCAACAGTTAATAATCTAGAG |
| HL-1-NTS-26 | TGTCCAACAGTTAATAATCTAGAG |
| HL-1-NTS-27 | TCTCCAACAGTTAATAATCTAGAG |
| HL-1-NTS-28 | TTACCAACAGTTAATAATCTAGAG |
| HL-1-NTS-29 | TTGCCAACAGTTAATAATCTAGAG |
| HL-1-NTS-30 | TTCCCAACAGTTAATAATCTAGAG |

Note: In this table, TS represent the template strand and NTS represent the non-template strand. The first four bases (from 5’ to 3’) of NTS represent the location of PAM or PAM-free sites. These targets were selected from specific genes of *L. acetotolerans* selected, including the endonuclease III gene (GenBank: CP051649.1, genome nucleotide positions from 244421 to 245002), the Ppx/GppA family phosphatase gene (GenBank: CP051649.1, genome nucleotide positions from 868146 to 869090), and the threonine/serine exporter family protein gene (GenBank: CP044496.1, genome nucleotide positions from 1483843 to 1484616). Meanwhile, other specific genes were also selected, including hypothetical protein gene (GenBank: CP138486.1, genome nucleotide positions from 2875513 to 2875818) of *L. plantarum*, 16S rRNA gene (GenBank: CP001973.1, genome nucleotide positions from 762956 to 763226) of *S. eriocheiris*, VP39 gene (GenBank: AY884234.1, genome nucleotide positions from 165 to 441) of white spot syndrome virus, N gene (GenBank: MN985325, genome nucleotide positions from 28840 to 29020) of SARS-CoV-2, the specific gene (GenBank: CP028535.1, genome nucleotide positions from 1224658 to 1224811) of *P. kudriazevii* and *invA* gene (GenBank: OY754829.1, genome nucleotide positions from 1340993 to 1341192) of *S. typhimurium*. Besides, other targets were selected from previously reported^[3]^, including spacers 4 and 5 of the Orf1ab gene, spacer 2 of S gene of SARS-CoV-2, spacer 1 of the human papillomavirus type 18 (HPV18) L1 gene.

**Table S4. Reference specific target sequences for the model application**

| **Name** | **Gene name** | **Sequence (5’ to 3’)** |
| --- | --- | --- |
| *Lactiplantibacillus plantarum* | acetylesterase gene (GenBank: CP039121.1, genome nucleotide positions from 925703 to 926627)^[8]^ | ATGGCACTGATTCGTATTAATTTTATGGCGGCTAGTTTACACCGGACCGTTCCCCTGATGGTGTGTTTACCAACTGATAAATTGGTCCCAGATGAGCAGGGCGTGCCGCGCCCGATTCAGGGTCCGTTTGCCACCCTGTACTTATTGCACGGTATCTTAGGTAGTGAAGTCGACTGGATTAGTGGCACACGAATCCAGCGCTGGGCCGACGAACGGAACCTGGCTGTCGTGATGCCAGCGGGCGAGAATAGTTTCTATACTGATCATCCTTGGTCTGGGGAGACGTATAGTCAATTTATTGGTCAGGAATTGATTGATTTTACACGCCGGACGTTTCCACTATCACACCAGCGTGACCAAACTTTTATTGGCGGATTATCAATGGGTGGTTACGGGGCGCTGTATAACGGACTAAAATTTCACGATACTTTTGGGGCCATTGTGAGTTTATCAGCCGGACTGAATGTGCGACCCGGAATGGAAAAGTTACCGGCAAAGCCTCAGTGGTTTGCGGAAACAGTCGCATATCAGCATGGCGTTTTTGGCCCAGATCTAGCAGCTGCTGGTCATTCGGAATTAAACCTGCAAGTGCTCGTAACTAATTTACTGGCAGCTCACGTCGTTTTACCCGCGATTTTTATGGCGATTGGCGATCAGGACGGCTTAAAGTCAGCGAATGATGAATTTGATCATTTTCTAACGACCAAAAAAGTTTCGCATGAATATTTGGTCGGTTCTGGAGCGCACGAGTGGGATTTCTGGGACCGCTATCTATTAAAAGCACTGAACTGGTTGCCACTAACTAATCAGGCGCCGGTGTTAATTCTGGGCATATTAAAGCTAATTGATGGGCTGCTTCAATCATTCTGTGTACAAATTATTAAAAAATATATTAATCTAAAAGCAGGGTGTCCCAAGGGATGA |
| *Vibrio parahaemolyticus* | *tlh* gene (GenBank: CP130652.1, genome nucleotide positions from 224210 to 224438)^[9]^ | AGCTACTCGAAAGATGATCCAGCGACCGATTGGGAATGGGCAAAAAACGAAGATGGTAGCTACTTCACCATTGACGGCTACTGGTGGAGCTCCGTTTCATTTAAAAACATGTTCTACACCAACACGTCGCAAAACGTTATCCGTCAGCGTTGTGAAGCAACATTAGATTTGGCGAACGAGAACGCAGACATTACGTTCTTCGCCGCTGACAATCGCTTCTCATACAACC |
| *Pseudomonas aeruginosa* | *oprL* gene (GenBank: CP131788.1, genome nucleotide positions from 5004772 to 5004967)^[10]^ | CGCGTAGTGCTGGAAGGCCACACCGACGAACGCGGCACCCGCGAGTACAATATGGCTCTGGGCGAGCGTCGTGCCAAGGCCGTTCAGCGCTACCTGGTGCTGCAGGGTGTTTCGCCGGCCCAGCTGGAACTGGTTTCCTATGGTAAAGAGCGTCCGGTCGCTACCGGCCACGACGAGCAGTCCTGGGCTCAGAACC |
| Hepatitis B virus | covalently closed circular DNA gene (GenBank: PP921796.1, genome nucleotide positions from 1521 to 1884)^[11]^ | GGGGCGCACCTCTCTTTACGCGGTCTCCCCGTCTGTGCCTTCTCATCTGCCGGACCGTGTGCACTTCGCTTCACCTCTGCACGTCGCATGGAAACCACCATGAACGCCCACCAGGTCTTGCCCAAGGTCTTATATAAGAGGACTCTTGGACTCTCAGCAATGTCAACGACCGACCTTGAGGCGTACTTCAAAGACTGTGTGTTTAAAGACTGGGAGGAGTTGGGGGAGGAGACTAGGTTAATGATCTTTGTACTAGGAGGCTGTAGGCATAAATTGGTCTGCGTACCAGCACCATGCAACTTTTTCACCTCTGCCTAATCATCTCATGTTCATGTCCTACTGTTCAAGCCTCCAAGCTGTGCCT |
| Human cytomegalovirus | UL83 gene (GenBank: KJ426589.1, genome nucleotide positions from 121562 to 121797)^[12]^ | TGGCTGGTGAAGGTGGGGGGCTCGCTGTACTGAGGCCCGCGCTGCAGCAGCAAGTCGATATCGAAAAAGAAGAGCGCAGCCACGGGATCGTACTGACGCAGTTCCACGGTCTCGCGTATCGCTTGTACCTCCAGGAAGATCTGCTGCCCGTTCATCAACAGGTTACCTGAGATGCTCAGGCCCGGGATGCTCTTGGGACACAGCAGCCCAAAATGCTCGTGTGAGGTAAAAGCCAC |
| *Aeromonas hydrophila* | gyrase subunit B (gyrB) gene (GenBank: MG310178.1, genome nucleotide positions from 294 to 550) | CCGAAGGTGTTCCACTTCACCACCGAGCAGGATGGCATCGGTGTGGAAGTAGCGATGCAGTGGAACGACGCCTATCAGGAAGGGGTCTACTGCTTCACCAACAACATTCCGCAGCGCGATGGTGGTACCCACCTGGTGGGCTTCCGTACCGCGCTGACCCGTACTCTGAACTCCTACATGGACAAAGAGGACTACAGCAAGAAGGCCAAGTCTGCCGCCAGTGGCGACGACGTGCGTGAAGGTCTGATTGCCGTTAT |
| *Edwardsiella tarda* | outer membrane protein A (ompA) gene (GenBank: CP100766.1, genome nucleotide positions from 2286985 to 2287250) | TGCCTTGAACTTACCGTTCACGTCACCGGTGTAGCCCATGCGGCCCAGCCAGTCGTAGCCCATTTCGAAGCCCAGGTACGGATTAGCCTGGTAACCGAAGAAAGCACCGGCGCCCAGCTGATTCGGATGAGACTTCGTGGAGTCCATATTCTCAAAGCTGTTACCGATGTAGTGGGACCAACCCAGTTTACCACCTACGTACCAGGTATCGTCTTTCGGAGCTGCCTGAGCTACGGTCGCGAAACCTGCCAGTGCCACTGCTAATG |
| Shrimp hemocyte iridescent virus | major capsid protein (MCP) gene (GenBank: KY681039.1, genome nucleotide positions from 1049 to 1207) | CGTAAGAGAACATGTGGTATCCGGTGAGTTCGGGAATGGCCGGTGCCTTGTAGTAGGGTTCGATCAGTGAGAAGTAATCGGCAGTCATCACGGGAATACGATCTGAAGATTCGTATCTAATGTTTGCGGTGCCTATGGGATCGAATGCGCTCTGATCTG |
| *Phomopsis* sp. | Beta-tubulin gene (GenBank: MK522108.1, genome nucleotide positions from 88 to 189) | CATCGTTACTGACCTCGACTTTCAGGCAAACCATCTCTGGCGAGCACGGTCTCGACAGCAATGGCGTGTATGCACCTCCTATTCCCTGTCTTCAAATCTCGT |
| *Botryosphaeria dothidea* | Beta-tubulin gene (GenBank: OQ410537.1, genome nucleotide positions from 112 to 267) | ATCATTCTCAGCGTGGGAGAACATCAATGACTAAACTGTAGCAGCTACAATGGCACCTCGGACCTTCAGCTCGAGCGCATGAACGTCTATTTCAACGAGGTACTCTCTCACTAATTAGACAAACACGTAAAGTATGGCAATCTTCTGAACGCGCAG |
| Human adenovirus | pIX gene (GenBank: LC215427.1, genome nucleotide positions from 3454 to 3861) | ATGAACGGGACCGGCGGGGCCTTCGAAGGGGGGCTTTTTAGCCCTTATTTGACAACCCGCCTGCCGGGATGGGCCGGAGTTCGTCAGAATGTGATGGGATCAACGGTGGACGGGCGCCCAGTGCTTCCAGCAAATTCCTCGACCATGACCTACGCGACCGTGGGGACGAGCTCGTCGCTTGACAGCACCGCCGCAGCCGCGGCAGCCGCAGCCGCCATGACAGCGACGAGACTGGCCTCGAGCTACATGCCCAGCAGCGGTAGCAGCCCCTCCATCCCCAGTTCCATCATCGCCGAGGAGAAACTGCTGGCCCTGCTGGCTGAGCTGGAAGCCCTGAGCCGCCAGCTGACCGCCCTGACCCAGGAGGTGTCCGAGCTCCGCGAACAGCAGCAGCAGCAAAATAAATGA |
| African swine fever virus | p285L gene (GenBank: KM111294.1, genome nucleotide positions from 11228 to 11512) | ATGCTTCTTGAGGAGGGAATTTTGATTGCTTTAATCCTGATATGGGTTGCCATTATACTATATAGTACCTTTCAGATCGGGATTAAACCTATTGGTTCTTCAGTGCCTCATAAATGTGGGTGCGGCCATCATGCATCAAATGAATCTCCTAGACCAGGATTTCATGGATGTGAGTATGACCATCCTCAGTTTGAGAGAACTCATTTCTGTAACAGGCATTCTTGTATTCCATGTTGTTCTCCACATCAAGCCTGTCTAAGATTCCAGTCTTATCACAGAGTTTAA |
| Human alphaherpesvirus | protein V32 gene (GenBank: KF811485.1, genome nucleotide positions from 60107 to 60538) | ATGGAATCGTCTAACATTAACGCGCTACAACAACCGTCGTCTATCGCACATCATCCGTCCAAACAGTGCGCTTCAAGTCTCAATGAAACAGTAAAAGATTCTCCCCCCGCGATTTATGAAGATAGGTTAGAACACACGCCGGTACAATTACCCCGCGACGGTACACCCCGAGACGTATGTTCTGTGGGACAGCTAACCTGTCGAGCATGTGCAACGAAACCTTTTCGCCTTAACCGCGACAGCCAATACGACTACTTAAACACATGTCCAGGGGGCCGTCATATTTCACTGGCACTGGAGATTATAACGGGTCGATGGGTTTGCATCCCGCGTGTGTTTCCGGATACCCCAGAGGAAAAATGGATGGCGCCATATATTATTCCAGACCGAGAACAACCATCATCAGGGGATGAAGATTCTGACACCGATTAA |
| Bat polyomavirus | small t antigen gene (GenBank: NC_026768.1, genome nucleotide positions from 4665 to 5075) | ATGGATAAGTTCATGGATAGAGAGGAGCTTAAGGAGCTTTGTGAGCTTCTTAATATTCCTGCCCATTGCTATGGGAATCTACCCATGATGAAGATAAACTACAAGAAAATGTGCCTCATCTATCACCCAGATAAAGGTGGAGATGTTGCAAAAATGCAGAGAATGAATGAACTCTGGCAAAAACTGCAGGATGGAGTTATTAATGCAAGAGATGAAGGCCCTGTGAGTAGATGGTTTTGGGAATACCAGGGCCAGACTTTAAGAGAATTTTTAGGGCCTGATTTTAATAAGAGATTTTGCAAGGTTTTTCCTACCTGCTTATATGCTTCTAAAGAATTTTGCTTTTGTGTATGCTGCTTGCTAAACAAGCAACATAAAATATATAAAGTAAAGAGGGAAAAAAATGCCTAG |
| Murmansk poxvirus | virion protein gene (GenBank: MF001304.1, genome nucleotide positions from 148114 to 148950) | ATGAACAGTTTTCAAGAAAAACGATTTTCGAGAGAAACTCTATTGAAAATGCCTTTTAGAATGGTTTTAACTGGAGGATCTGGATCTGGGAAAACAATCTATTTACTATCCCTATTTTCAACACTTGTTAAAAAATATAAACATATATTTCTGTTTACACCAGTGTATAATCCAGACTATGATGGATACATTTGGCCTGATCATATTAACACAGTCGAGACACCAGAGGCTCTTGAGTATGCCCTAAGAGACACGAAGATAAAAATCTCTAAATATATTACAAAGAATAGTCATAAGAAAGCGGAACATTTCTTGATTATAGTAGATGATATGGGAGAGAAACTATCAAAGTCGGGTACCATAATAGATTTTCTAAATTTTGGACGACATTTGAACACTTCTCTCATAATGTTATGTCAAACATATAGACATGTTCCTGTAGCTGGAAGATCTAATATAACACATTTCTGTAGTTTTAATATTTCTATTTCAGATGCTGAGAATATGTTGAGATCAATGCCCGTTAAAGGAAAGCGTAAAGATATATTATACATGTTAAATATAATTCAAAATGGTAAATCTAATACACGACTAGCAATCATAATTGAAGACTCTGTATTTTGCGAAGGAGAGTTACGTATATGTACAGATATAGCAGATAAAGATGTTATTGAACAAAAATTGAACCCAGATATACTTTTGAAACAATTCTCACACATGAAAAAGAATCTCAACACTATAATTGAAGCAAATAATACACTCATTAATCACTCAAAATCATCATCATTATCTAAATCATCATCTAAATCATCATCATCATCATCTTCAGACGAATAA |

**Table S5. crRNA-DNA sequences for testing the prediction performance of models**

| **Name** | **Sequence (5’ to 3’)** |  |
| --- | --- | --- |
| **crRNA sequences** | |  |
| Lp-crRNA | UAAUUUCUACUAAGUGUAGAUGUCCGUUAUACAGCGCCCCG |  |
| Vp-crRNA | UAAUUUCUACUAAGUGUAGAUUCAGCGGCGAAGAACGUAAU |  |
| Psa-crRNA | UAAUUUCUACUAAGUGUAGAUUCGGUGUGGCCUUCCAGCAC |  |
| HBV-crRNA | UAAUUUCUACUAAGUGUAGAUUGCCUACAGCCUCCUAGUAC |  |
| HCMV-crRNA | UAAUUUCUACUAAGUGUAGAUUGCUGCUGCAGCGCGGGCCU |  |
| Ahy-crRNA | UAAUUUCUACUAAGUGUAGAUUUGGUGAAGCAGUAGACCCC |  |
| Eth-crRNA | UAAUUUCUACUAAGUGUAGAUCGACCGUAGCUCAGGCAGCU |  |
| SHIV-crRNA | UAAUUUCUACUAAGUGUAGAUAGAUCGUAUUCCCGUGAUGA |  |
| Phs-crRNA | UAAUUUCUACUAAGUGUAGAUGAGACCGUGCUCGCCAGAGA |  |
| Bdo-crRNA | UAAUUUCUACUAAGUGUAGAUCGUGUUUGUCUAAUUAGUGA |  |
| Hadv-crRNA | UAAUUUCUACUAAGUGUAGAUACGAACUCCGGCCCAUCCCG |  |
| ASFV-crRNA | UAAUUUCUACUAAGUGUAGAUAUGCAUGAUGGCCGCACCCA |  |
| HALV-crRNA | UAAUUUCUACUAAGUGUAGAUCUGUCCCACAGAACAUACGU |  |
| BPV-crRNA | UAAUUUCUACUAAGUGUAGAUCAUGGGUAGAUUCCCAUAGC |  |
| MPOV-crRNA | UAAUUUCUACUAAGUGUAGAUACGGGCAUUGAUCUCAACAU |  |
| **Name** | **Sequence (5’ to 3’)** |  |
| **Target DNA sequence** | | |
| Lp-crRNA-TS | CGGGGCGCTGTATAACGGACTAAA |  |
| Vp-crRNA-TS | ATTACGTTCTTCGCCGCTGACAAT |  |
| Psa-crRNA-TS | GTGCTGGAAGGCCACACCGACGAA |  |
| HBV-crRNA-TS | GTACTAGGAGGCTGTAGGCATAAA |  |
| HCMV-crRNA-TS | AGGCCCGCGCTGCAGCAGCAAGTC |  |
| Ahy-crRNA-TS | GGGGTCTACTGCTTCACCAACAAC |  |
| Eth-crRNA-TS | AGCTGCCTGAGCTACGGTCGCGAA |  |
| SHIV-crRNA-TS | TCATCACGGGAATACGATCTGAAG |  |
| Phs-crRNA-TS | TCTCTGGCGAGCACGGTCTCGACA |  |
| Bdo-crRNA-TS | TCACTAATTAGACAAACACGTAAA |  |
| Hadv-crRNA-TS | CGGGATGGGCCGGAGTTCGTCAGA |  |
| ASFV-crRNA-TS | TGGGTGCGGCCATCATGCATCAAA |  |
| HALV-crRNA-TS | ACGTATGTTCTGTGGGACAGCTAA |  |
| BPV-crRNA-TS | GCTATGGGAATCTACCCATGATGA |  |
| MPOV-crRNA-TS | ATGTTGAGATCAATGCCCGTTAAA |  |
| Lp-crRNA-NTS | TTTAGTCCGTTATACAGCGCCCCG |  |
| Vp-crRNA-NTS | ATTGTCAGCGGCGAAGAACGTAAT |  |
| Psa-crRNA-NTS | TTCGTCGGTGTGGCCTTCCAGCAC |  |
| HBV-crRNA-NTS | TTTATGCCTACAGCCTCCTAGTAC |  |
| HCMV-crRNA-NTS | GACTTGCTGCTGCAGCGCGGGCCT |  |
| Ahy-crRNA-NTS | GTTGTTGGTGAAGCAGTAGACCCC |  |
| Eth-crRNA-NTS | TTCGCGACCGTAGCTCAGGCAGCT |  |
| SHIV-crRNA-NTS | CTTCAGATCGTATTCCCGTGATGA |  |
| Phs-crRNA-NTS | TGTCGAGACCGTGCTCGCCAGAGA |  |
| Bdo-crRNA-NTS | TTTACGTGTTTGTCTAATTAGTGA |  |
| Hadv-crRNA-NTS | TCTGACGAACTCCGGCCCATCCCG |  |
| ASFV-crRNA-NTS | TTTGATGCATGATGGCCGCACCCA |  |
| HALV-crRNA-NTS | TTAGCTGTCCCACAGAACATACGT |  |
| BPV-crRNA-NTS | TCATCATGGGTAGATTCCCATAGC |  |
| MPOV-crRNA-NTS | TTTAACGGGCATTGATCTCAACAT |  |

**Reference**

[1] D. C. Swarts, M. Jinek, *Molecular Cell* **2019**, *73* (3), 589.

[2] T. Yamano, B. Zetsche, R. Ishitani, F. Zhang, H. Nishimasu, O. Nureki, *Molecular Cell* **2017**, *67* (4), 633.

[3] S. Lu, X. Tong, Y. Han, K. Zhang, Y. Zhang, Q. Chen, J. Duan, X. Lei, M. Huang, Y. Qiu, D.-Y. Zhang, X. Zhou, Y. Zhang, H. Yin, *Nature Biomedical Engineering* **2022**, *6* (3), 286.

[4] B. Huang, L. Guo, H. Yin, Y. Wu, Z. Zeng, S. Xu, Y. Lou, Z. Ai, W. Zhang, X. Kan, Q. Yu, S. Du, C. Li, L. Wu, X. Huang, S. Wang, X. Wang, *Imeta* **2024**, e214.

[5] H. C. Metsky, N. L. Welch, P. P. Pillai, N. J. Haradhvala, L. Rumker, S. Mantena, Y. B. Zhang, D. K. Yang, C. M. Ackerman, J. Weller, P. C. Blainey, C. Myhrvold, M. Mitzenmacher, P. C. Sabeti, *Nature Biotechnology* **2022**, *40* (7), 1123.

[6] J. Zhang, X. Guan, J. Moon, S. Zhang, Z. Jia, R. Yang, C. Hou, C. Guo, M. Pei, C. Liu, *Nucleic Acids Research* **2024**, gkae1124.

[7] T. Yamano, H. Nishimasu, B. Zetsche, H. Hirano, Ian M. Slaymaker, Y. Li, I. Fedorova, T. Nakane, Kira S. Makarova, Eugene V. Koonin, R. Ishitani, F. Zhang, O. Nureki, *Cell* **2016**, *165* (4), 949.

[8] R. Du, S. Wang, Q. Wu, Y. Xu, *Systems Microbiology and Biomanufacturing* **2023**, *3* (4), 593.

[9] X. Chen, L. Wang, F. He, G. Chen, L. Bai, K. He, F. Zhang, X. Xu, *Analytical Chemistry* **2021**, *93* (42), 14300.

[10] X. Qiu, X. Liu, R. Wang, X. Ma, L. Han, J. Yao, Z. Li, *Microbiology Spectrum* **2023**, *11* (1), e03523.

[11] X. Zhang, Y. Tian, L. Xu, Z. Fan, Y. Cao, Y. Ma, H. Li, F. Ren, *Hepatology International* **2022**, *16* (2), 306.

[12] M. H. Liu, X. C. Guo, M. L. Sun, J. L. Li, S. H. Liu, Y. Z. Chen, D. Y. Wang, L. Wang, Y. Z. Li, J. Yao, Y. Li, Y. Q. Pan, *Frontiers in Cellular and Infection Microbiology* **2024**, *14*, 1430302.
